# Identification of prediagnostic metabolites associated with prostate cancer risk by untargeted mass spectrometry‐based metabolomics: A case‐control study nested in the Northern Sweden Health and Disease Study

**DOI:** 10.1002/ijc.34223

**Published:** 2022-08-12

**Authors:** Johnny R. Östman, Rui C. Pinto, Timothy M. D. Ebbels, Elin Thysell, Göran Hallmans, Ali A. Moazzami

**Affiliations:** ^1^ Department of Molecular Sciences Swedish University of Agricultural Sciences Uppsala Sweden; ^2^ Department of Epidemiology and Biostatistics, MRC‐PHE Centre for Environment and Health School of Public Health, Imperial College London London UK; ^3^ UK Dementia Research Institute Imperial College London London UK; ^4^ Department of Metabolism, Digestion and Reproduction Imperial College London London UK; ^5^ Department of Medical Biosciences Umeå University Umeå Sweden; ^6^ Department of Public Health and Clinical Medicine, Nutritional Research Umeå University Umeå Sweden

**Keywords:** liquid chromatography‐mass spectrometry, nested case‐control study, prostate cancer, risk biomarkers, untargeted metabolomics

## Abstract

Prostate cancer (PCa) is the most common cancer form in males in many European and American countries, but there are still open questions regarding its etiology. Untargeted metabolomics can produce an unbiased global metabolic profile, with the opportunity for uncovering new plasma metabolites prospectively associated with risk of PCa, providing insights into disease etiology. We conducted a prospective untargeted liquid chromatography‐mass spectrometry (LC‐MS) metabolomics analysis using prediagnostic fasting plasma samples from 752 PCa case‐control pairs nested within the Northern Sweden Health and Disease Study (NSHDS). The pairs were matched by age, BMI, and sample storage time. Discriminating features were identified by a combination of orthogonal projection to latent structures‐effect projections (OPLS‐EP) and Wilcoxon signed‐rank tests. Their prospective associations with PCa risk were investigated by conditional logistic regression. Subgroup analyses based on stratification by disease aggressiveness and baseline age were also conducted. Various free fatty acids and phospholipids were positively associated with overall risk of PCa and in various stratification subgroups. Aromatic amino acids were positively associated with overall risk of PCa. Uric acid was positively, and glucose negatively, associated with risk of PCa in the older subgroup. This is the largest untargeted LC‐MS based metabolomics study to date on plasma metabolites prospectively associated with risk of developing PCa. Different subgroups of disease aggressiveness and baseline age showed different associations with metabolites. The findings suggest that shifts in plasma concentrations of metabolites in lipid, aromatic amino acid, and glucose metabolism are associated with risk of developing PCa during the following two decades.

## Graphical abstract

What's new?

The identification of novel metabolic risk factors is a critical step in the advance of prevention strategies for prostate cancer. In the present study, using untargeted liquid chromatography‐mass spectrometry metabolomics, the authors examined prospective associations between circulating metabolites and prostate cancer risk. The analyses uncover positive associations between prostate cancer risk and aromatic amino acids, free fatty acids, uric acid, and phospholipids. Meanwhile, glucose was negatively associated with risk among older participants. The findings cast light on potentially clinically relevant associations between prostate cancer risk and plasma concentrations of metabolites generated by aromatic amino acid, lipid, and glucose metabolism.

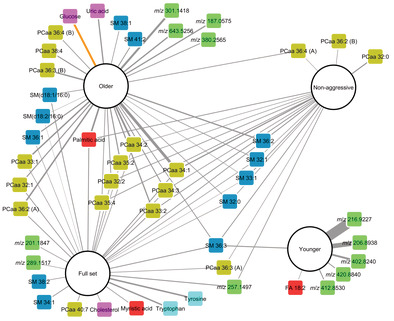

**Abbreviations**
ATBC: Alpha‐Tocopherol, Beta‐Carotene Cancer Prevention Study
CI: confidence interval
CLR: conditional logistic regression
CV‐ANOVA: cross‐validated analysis of variance
EPIC: European Prospective Investigation into Cancer and Nutrition Study
HMDB: Human Metabolome Database
LC‐MS: liquid chromatography‐mass spectrometry
MSI: metabolomics standards initiative
NSHDS: Northern Sweden Health and Disease Study
OPLS‐EP: orthogonal projection to latent structures‐effect projections
PCa: prostate cancer
PCs: phosphatidylcholines
PI3K: phosphoinositide 3‐kinase
PLCO: Prostate, Lung, Colorectal and Ovarian Cancer Screening Trial
PSA: prostate‐specific antigen
*PTEN*
: phosphatase and tensin homolog
QToF: quadrupole‐time‐of‐flight
SMs: sphingomyelins
T2D: type 2 diabetes
VIP: variable importance on projection
XIC: extracted ion chromatograms

## 1 INTRODUCTION

Prostate cancer (PCa) is the most common form of cancer in males in many countries, especially in Europe and the United States.^1^ PCa has large geographical variation in its reported incidence, which may be related to differences in the intensity of screening and/or exposure to etiologically relevant environmental and lifestyle factors.

PCa is a complex disease, and efforts have been made to identify genetic and adjustable risk factors in order to improve understanding of how PCa develops.^2^ The World Cancer Research Fund lists and evaluates over 50 potential PCa risk factors, related to diet, nutrition, and physical activity, in its updated 2018 report, but the body of evidence and number of identified risk factors are still meager.^3^ The human metabolome, composed of the pool of metabolites, is the final read‐out of gene‐environment interactions^4^ and can be analyzed to generate new insights into the etiology of PCa by identifying novel metabolic risk factors, which is an important step for establishing prevention strategies.

Attempts have been made to identify prospective associations between prediagnostic concentrations of plasma and serum metabolites and risk of PCa incidence^5^, ^6^, ^7^, ^8^, ^9^, ^10^, ^11^, ^12^, ^13^, ^14^ or death due to PCa.^15^, ^16^, ^17^ Glycerophospholipids have been associated with risk of PCa, although to varying degrees.^9^, ^10^, ^11^, ^12^ In addition, higher prediagnostic concentrations of metabolites from redox (inversely) and dipeptide (positively) pathways have been associated with risk of lethal PCa.^17^ Previous studies have differed with regard to metabolomics methodologies, disease subtype categorization, size, outcome, and handling of follow‐up time and time since last meal (fasting status at the time of sampling). Varying time since last meal^18^, ^19^ and presence of subclinical PCa at the time of sample collection (baseline)^12^ can induce variations in the concentrations of metabolites, which are not directly related to estimation of disease risk.

In a previous nested case‐control study,^10^ we attempted to reduce these unrelated variations by using only subjects sampled after overnight fasting and by using only cases with a follow‐up time (time between sample collection and diagnosis) of at least 5 years (follow‐up time ≥5 years). In that study (n = 777 pairs), which was nested in the Northern Sweden Health and Disease Study (NSHDS), we used targeted mass spectrometry and nuclear magnetic resonance (NMR) metabolomics to screen prospectively for prediagnostic associations between plasma metabolites and risk of developing PCa.^10^ We identified several glycerophospholipids that were positively associated with overall risk and risk of aggressive PCa, with a strong association for lysophosphatidylcholine 17:0.^10^

One limitation of the targeted metabolomics approach is the limited number of metabolites and limited range of metabolite classes that can be detected. In order to increase the metabolite coverage, in the present study we used an untargeted mass spectrometry‐based metabolomics approach for prospective investigation of associations between prediagnostic concentrations of plasma metabolites and PCa risk in the same nested case‐control study previously analyzed using a targeted metabolomics approach.^10^

To our knowledge, our study, with a follow‐up time of at least 5 years (maximum follow‐up time = 20 years), is the largest prospective study to date of prediagnostic plasma metabolites and PCa risk using untargeted mass spectrometry‐based metabolomics.

## 2 MATERIALS AND METHODS

### 2.1 Study cohort

We used blood plasma samples from the Northern Sweden Health and Disease Study (NSHDS).^20^ The present study was conducted as a nested case‐control study within the longitudinal population‐based NSHDS cohort, to investigate prospective associations between plasma metabolites and risk of developing PCa. In brief, all residents in Västerbotten County, Sweden, were asked to enroll in the cohort at 40, 50, and 60 years of age. Upon enrolment, the participants were asked to undergo a baseline examination after overnight fasting, fill in a food frequency questionnaire, and donate a blood sample for future research purposes. The blood samples were heparinized, centrifuged and stored at −80°C within 1 hour of sampling (94.5% of blood samples collected 07:30‐10:00 am).^20^

### 2.2 Study design

The study design is described in detail in our previous targeted metabolomics study.^10^ To summarize, of the heparinized blood samples available in the NSHDS, 777 male case‐control pairs were selected (follow‐up period until 2012) using the following four inclusion criteria for case selection: (a) overnight fasting, (b) no previous cancer diagnosis of any kind, (c) at least 5 years between enrolment and PCa diagnosis, and (d) no type 2 diabetes diagnosis at baseline. The cases and controls were matched by age, freezer storage time, and body mass index (BMI). The controls were also overnight fasting with no type 2 diabetes diagnosis at baseline. Due to insufficient sample volume in either the case or control sample, the final case‐control set in the study was reduced to 752 pairs. Baseline characteristics are compiled in Table 1.

**TABLE 1:** Baseline characteristics of individuals participating in the study

	All PCa (n = 752:752)		Nonaggressive PCa (587:587)		Aggressive PCa (n = 165:165)		Younger^a^ (n = 326:326)		Older^b^ (n = 426:426)	
	Controls	Cases	Controls	Cases	Controls	Cases	Controls	Cases	Controls	Cases
*Clinical characteristics* ^c^ ^,^ ^d^										
Age (years)^e^	59.8 (40.3‐60.3)	59.8 (40.4‐60.3)	59.5 (40.3‐60.3)	59.4 (40.3‐60.3)	59.9 (49.8‐60.3)	59.9 (49.8‐60.4)	50 (40.1‐50.3)	50 (40.1‐50.4)	60 (59.7‐60.3)	60 (59.5‐60.4)
Height (cm)	177 (167‐187)	177 (167‐186)	178 (167‐187)	177 (167‐186)	177 (167‐187)	176 (167‐186)	179 (167‐188)	178 (168‐187)	176 (167‐186)	176 (166‐186)
Weight (kg)	81 (66‐100)	81 (66‐100)	81 (66‐101)	81 (66‐101)	82 (64‐98)	81 (64‐100)	81 (67‐101)	81 (66‐100)	82 (65‐99)	81 (65‐100)
BMI (kg/m^2^)^e^	25.8 (21.9‐31.3)	25.7 (21.8‐31.2)	25.7 (21.9‐31.3)	25.7 (21.8‐31.4)	25.9 (21.8‐31.0)	25.9 (21.6‐31.1)	25.5 (22.0‐31.2)	25.5 (22.0‐31.1)	26.0 (21.6‐31.5)	26.0 (21.4‐31.4)
SBP (mmHg)	130 (110‐165)	130 (110‐165)	130 (110‐165)	130 (110‐160)	137 (110‐170)	135 (105‐171)	125 (106‐155)	125 (105‐152)	137 (110‐170)	137 (110‐170)
DBP (mmHg)	82 (65‐100)	83 (65‐100)	81 (65‐100)	82 (67‐100)	84 (67‐100)	84 (65‐100)	80 (64‐98)	80 (65‐98)	84 (68‐100)	85 (70‐100)
Total cholesterol (mmol/L)	5.8 (4.1‐7.9)	5.8 (4.2‐7.8)	5.8 (4.1‐7.8)	5.8 (4.2‐7.8)	5.8 (4.5‐7.8)	5.9 (4.2‐7.9)	5.8 (4.2‐7.7)	5.8 (4.1‐7.7)	5.8 (4.1‐7.9)	5.9 (4.2‐7.8)

Abbreviations: BMI, body mass index; DBP, diastolic blood pressure; PCa, prostate cancer; SBP, systolic blood pressure.

^a^ Data available for >95% of the case‐controls.

^b^ Variables are listed as median (5th‐95th percentile).

^c^ Factors used for sample matching. Sample storage time is an additional matching factor.

^d^ Baseline age of 40 and 50 years.

^e^ Baseline age of 60 years.

The cases were also stratified into subgroups that were analyzed independently (statistically), based on baseline age (at enrolment) (40‐ and 50‐year‐olds, and 60‐year‐olds) and by aggressive and nonaggressive PCa at diagnosis.^21^ Cases fulfilling at least one of the following criteria were considered as being aggressive PCa: Tumor poorly differentiated (Gleason score 8‐10 or grade 3 in the former three‐level World Health Organization grading system, in which grade 3 indicates the lowest level of differentiation),^21^, ^22^, ^23^ tumor nonlocalized (T3‐4), bone metastases present (M1), lymph node metastases present (N1), serum prostate‐specific antigen (PSA) level >50 ng/mL at time of diagnosis or fatal PCa (ICD‐10 code C61) by January 2017 regardless of tumor state at diagnosis. All other cases were classified as being nonaggressive PCa. In total, 587 cases were classified as nonaggressive PCa, while 165 were considered aggressive (Table 2). Due to the low numbers of PCa cases enrolled and sampled at age 40, this subgroup (45 pairs) was merged with the subgroup of 50‐year‐olds (281 pairs). The younger subgroup (40‐ and 50‐year‐olds) thus consisted of 326 pairs, while the older subgroup of subjects (aged 60 years at baseline) contained 426 case‐control pairs.

**TABLE 2:** Characteristics of prostate cancer (PCa) cases at the time of diagnosis

	All PCa (n = 752)	Nonaggressive PCa (n = 587)	Aggressive PCa (n = 165)
Tumor grade^a^			
High	111	0	111
Low/intermediate	635	583	52
Missing	6	4	2
Primary tumor			
Nonassessed (TX)	14	13	1
Nonpalpable (T1)	422	391	31
Localized (T2)	236	178	58
Nonlocalized (T3, T4)	74	0	74
Missing	6	5	1
Lymph node metastases			
Nonassessed (NX)	657	520	137
Not present (N0)	84	63	21
Lymph node (N1)	6	0	6
Missing	5	4	1
Bone metastases			
Nonassessed (MX)	394	360	34
Not present (M0)	309	223	86
Bone metastases (M1)	44	0	44
Missing	5	4	1
Serum PSA			
≤50 ng/mL	681	582	99
>50 ng/mL	66	0	66
Missing	5	5	0
Fatal PCa outcome by January 2017			
Fatal outcome	39	0	39
Disease aggressiveness^b^			
Nonaggressive	587	587	0
Aggressive	165	0	165

^a^ Tumors were considered high grade if they were given Gleason sum score ≥8, or G3 according to the former three‐level WHO grading system. Tumors graded low/intermediate had Gleason sum score ≤7, or G1‐G2 according to the former WHO grading system.

^b^ Prostate cancer cases were considered aggressive if they fulfilled at least one of the following criteria: High tumor grade, nonlocalized tumor (T3‐4), bone metastasis (M1), lymph node metastasis (N1), serum prostate‐specific antigen (PSA) >50 ng/mL or fatal prostate cancer outcome by January 2017.

### 2.3 Sample preparation and liquid chromatography‐mass spectrometry

The plasma samples were prepared according to the method described by Evans et al^24^ with minor modifications. Each plasma sample was measured with both positive and negative ionization mode. The two‐paired samples were analyzed immediately after each other, with case‐control injection order randomized in order to minimize any impact of instrument drift.^25^ Chromatographic separation was carried out on Waters BEH C18 (1.7 μm, 2.1 × 100 mm) columns (Waters, Milford, Massachusetts). For positive mode separation, (A) 0.1% formic acid in water and (B) 0.1% formic acid in methanol were used as eluents. For negative mode separation, (A) 6.5 mM ammonium bicarbonate in water and (B) 6.5 mM ammonium bicarbonate in methanol (ammonium bicarbonate soluble upon sonication) were used as eluents. The following gradient profile was used in both modes: 2% B to 70% B in 4 minutes, 70% to 98% B in 1.5 minutes, 98% B for 2.9 minutes, 2% B in 1 minutes, and 2% B for 2.6 minutes.

Mass spectrometry analyses were carried out on an electrospray quadrupole‐time‐of‐flight (QToF) mass spectrometer (Bruker maXis impact, Bruker Daltonics, Bremen, Germany). Positive and negative ionization mode profile sample spectra in the range 50 to 1200 *m/z* were collected. The samples were analyzed in four batches in both positive and negative mode. MS^2^ spectra were also collected, in order to assist in annotation of metabolite features found to be significantly associated with PCa risk. Positive and negative ionization mode collisional‐induced dissociation (CID) fragment ion spectra (MS^2^) were collected for all filtered features, irrespective of the chromatographic conditions in which the feature was first detected. The [M+H]^+^ and [M−H]^−^‐adducts of all filtered features were individually isolated (isolation width 0.5 *m/z*) and fragmented. For additional details of sample preparation, reversed phase chromatography and high‐resolution mass spectrometry conditions, see Supporting Information Materials and Methods.

### 2.4 Data processing

The liquid chromatography‐mass spectrometry (LC‐MS) profile spectra raw data were converted to centroided mzML format using Bruker CompassXport (v. 3.0.9.2) and processed using the R (https://www.r-project.org/, v. 3.5.1) package *XCMS* (v. 3.4.2).^26^ Data processing included peak picking, grouping, and filling of zero intensity features (for XCMS parameters, see Supporting Information Materials and Methods). The processing resulted in a peak table in which the spectral features in each sample were assigned an *m/z*‐value, a retention time value, and an intensity value. Data processing was applied separately on the four batches for each polarity.

An in‐house algorithm^27^ was used to find correspondence between (metabolite) features in the different batches. The procedure was defined as follows: (a) All possible matches within adequately defined median retention time (RT) and *m/z*‐thresholds were identified batch‐wise. This resulted in clusters, including features from the four batches, and in interbatch alignment of both RT and *m/z*. (b) In each cluster, only features that matched features in all other batches were allowed (ie, cliques), while the rest were deleted. (c) In cases of match multiplicity, a penalization score was created to decide the best match. This score was defined after batch‐to‐batch alignment as the Euclidean distance of RT and *m/z* difference (both normalized) between each of the features in the match. Processing was performed separately on the positive and negative mode datasets and allowed only features present in all four batches. The two resulting datasets (one for the four batches analyzed in positive mode and one for the four batches analyzed in negative mode) were then merged, resulting in a single dataset containing the 1100 metabolite features in total detected in all four positive‐mode and four negative‐mode batches. The data were not adjusted for intrabatch instrument drift, since all statistical comparisons were made pair‐wise, with the two paired samples being analyzed consecutively in randomized order.^25^

### 2.5 Identification of discriminating features

As an initial step to identify features discriminating between cases and controls, conservative multivariate and univariate statistical analyses were applied on the full dataset of 752 case‐control pairs, and separately on the individual subgroups of disease aggressiveness (587 nonaggressive/165 aggressive pairs) and baseline age (326 younger/426 older pairs). Prospective associations between discriminating features (identified in previous steps) and PCa risk were evaluated by conditional logistic regression (CLR) (for details of CLR, see Section 2.6).

An extension of the multivariate orthogonal projection to latent structures model (OPLS) optimized for analysis of paired or matched samples, named OPLS‐effect projections (OPLS‐EP),^25^ was used to identify features that discriminated the cases from their respective controls (paired). Intensity differences between cases and their respective controls for each feature (*x*‐variables) were unit variance‐scaled and modeled toward an all‐ones *y*‐vector. For each of the stratifications (all, nonaggressive, aggressive, younger, older), three models were constructed: a model containing all cases, a model excluding the extreme 1% of cases based on Hotelling's *T* ^2^ range, and a model excluding the extreme 5% of cases. The three different models were considered, in order to ensure that all features in the dataset with significant differences between cases and controls were included, while also accounting for the impact of potential outliers. The significance of the models was determined using 7‐fold cross‐validated analysis of variance (CV‐ANOVA), with a *P*‐value <.05 considered significant.^28^ Features with variable importance on projection (VIP) values ≥1.5 and VIP lower 95% confidence interval (CI) >0 in at least one of the three models were considered as discriminating between cases and controls.^29^ Multivariate statistical analyses were performed using SIMCA 16 (Sartorius Stedim Data Analytics AB, Umeå, Sweden).

In the conservative univariate analysis, two‐sided Wilcoxon signed‐rank test was used to find discriminating features. The threshold for statistical significance was set at *P* < 4.55 × 10^−5^, based on Bonferroni correction for 1100 tests. Univariate statistical analyses were performed using R 3.5.1.

### 2.6 Metabolite feature filtering

**FIGURE 1:**
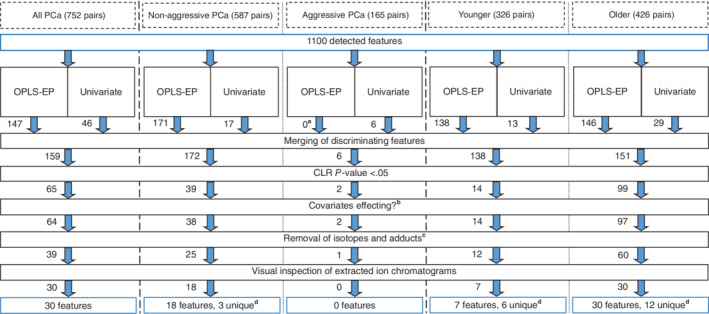
Flowchart showing the workflow in extracting and filtering metabolite features significantly associated with overall future prostate cancer (PCa) development and after stratification by disease aggressiveness and baseline age [younger (40‐ and 50‐year‐olds); older (60‐year‐olds)]. ^a^Orthogonal projections to latent structures‐effect projections (OPLS‐EP) models not significant via cross‐validated analysis of variance (CV‐ANOVA), *P* > .05. ^b^Features were excluded if the conditional logistic regression (CLR) *P*‐value rose above .05 or if the odds ratio changed by more than 10% after inclusion of the covariates exact age, body mass index, alcohol consumption (<10, 10‐19, 20‐39, ≥40 g/day), and smoking status (no, past, current, unknown) in the CLR model. ^c^Isotope and adduct filtering was done by assessing whether features with the same retention time differed in *m/z* corresponding to ^13^C isotope differences (Δ = n × 1.0033), Na^+^‐adducts (Δ = 21.9819) or in‐source H_2_O (Δ = 18.0153) or NH_3_ (Δ = 17.0266)‐loss, retaining (by decreasing priority) the [M ± H]^±^ adduct, the lowest *m/z* isotopologue, or an in‐source fragment rather than sodium adduct. ^d^Unique features are features which were not found to be significantly associated in the full dataset of 752 pairs.

The discriminating metabolite features obtained in multivariate or univariate statistical analyses were subjected to a four‐step filtering process in order to identify features significantly prospectively associated with PCa risk (filtered features) in each dataset (full dataset, aggressive/nonaggressive, younger/older). The filtering process, and the number of features remaining after each step, are graphically summarized in Figure 1.

Step 1: The prospective association between each discriminating feature and the risk of future PCa was evaluated by CLR. The feature intensities were log_2_‐transformed prior to analysis, in order to relate the resulting odds ratios to a doubling of signal intensity in the samples. The analysis was performed using the procedure PHREG in SAS (version 9.4, SAS Institute, Cary, North Carolina), with the case‐control pair number (pairing identifier) as stratum. Discriminating features with a CLR *P*‐value >.05 were excluded.
Step 2: The remaining features were further tested for association with covariates. Each CLR model was adjusted for exact age, BMI, smoking status (no, past, current, unknown), and alcohol consumption (<10, 10‐19, 20‐39, ≥40 g/day).^10^, ^12^ Features for which the CLR *P*‐value exceeded .05 or the odds ratio (OR) changed by more than 10% after covariates adjustment were excluded. For the remaining features, the results from the unadjusted CLR models are presented in the Section 3.
Step 3: The remaining features were further filtered by manual removal of features with the same origin (^13^C isotopes, Na^+^‐adducts, and in‐source losses). Isotope and adduct filtering was performed by assessing whether features with the same retention time differed in *m/z* corresponding to ^13^C isotope differences (Δ = *n* × 1.0033), Na^+^‐adducts (Δ = 21.9819) or in‐source H_2_O (Δ = 18.0153) or NH_3_ (Δ = 17.0266) loss, retaining (by decreasing priority) the [M ± H]^±^ adduct, the lowest *m/z* isotopologue or an in‐source fragment rather than an Na^+^‐adduct.
Step 4: As a final quality control step, extracted ion chromatograms (XIC) of the remaining features were plotted using the R package *peakPantheR* (v 1.0.0).^30^ The XICs of features were visually inspected to remove those with non‐Gaussian peaks and to validate the results of the peak picking process.

Features exceeding all filtering criteria found within a subgroup (nonaggressive/aggressive or younger/older) were further investigated for possible differential associations (heterogeneity). The features were allowed different associations with PCa risk for the two categories within aggressiveness and age group, respectively. Comparisons were made using a Wald test for equal regression coefficients, on the log_2_‐transformed feature data in SAS 9.4.

### 2.7 Annotation of filtered metabolite features

Metabolite feature annotation was based on accurate mass of the parent ion and accurate masses of the CID fragments in both positive and negative mode (when applicable). When two different phospholipid features with the same sum of side‐chain carbons and unsaturations were annotated, they were given an additional (A) or (B) suffix. The annotation of individual side‐chain fatty acids was determined based on the accurate masses of detected free fatty acid, lysophosphatidylcholine or lysosphingomyelin CID fragments (when applicable). The relative positions of side‐chain fatty acids in phosphatidylcholines (PCs) were suggested (when applicable) based on CID fragment signal intensities, with the stronger of the two potential lyso‐fragment signals corresponding to the more stable secondary carbocation retaining the fatty acid on carbon 3/*sn* − 1. Comparisons of spectral data with databases (Human Metabolome Database [HMDB], METLIN, mzCloud, Lipid Maps, MassBank) were conducted in order to aid annotation. For details regarding annotation procedures, see Supporting Information Materials and Methods.

## 3 RESULTS

### 3.1 Baseline and case characteristics

The baseline characteristics of the PCa cases and controls are presented in Table 1 for the whole set of 752 case‐control pairs and for the four subgroups (nonaggressive, aggressive, younger, older). The factors used for sample matching (age and BMI) were evenly distributed between the cases and controls, as intended by the study design.

The data used for assessing the degree of aggressiveness of the 752 PCa cases are presented in Table 2, including data for the full set of 752 cases and for the subgroups of nonaggressive and aggressive PCa individually. In total, 587 cases were classified as nonaggressive, while 165 cases (22% of cases) were considered aggressive, based on tumor grade, tumor stage, serum PSA level, and fatal PCa by January 2017. Within the younger subgroup, 43 cases (13.2%) were deemed aggressive, while in the older subgroup 122 cases (28.6%) were considered aggressive.

### 3.2 Identification of discriminating metabolite features, feature filtering, and differential associations

Discriminating features were first identified with multivariate and univariate statistics (OPLS‐EP and two‐sided Wilcoxon signed‐rank test, followed by Bonferroni correction) in each of the five datasets (full, nonaggressive/aggressive, younger/older) (Figure 1, Table S1). The features found by multivariate and univariate statistics were merged and subjected to a four‐step filtering process, in order to identify features prospectively associated with PCa risk in each of the five datasets (Figure 1, Table S1) (for a summary of the number of discriminating metabolite features found using multivariate statistics, univariate statistics or both, see Figure S1). Metabolite features identified as discriminating by the multivariate statistics, and their VIP values and corresponding odds ratios, are compiled in Table S2. Features identified as discriminating by the univariate statistics, and their *P*‐values and corresponding odds ratios, are compiled in Table S3. There were differential associations between the disease aggressiveness categories for none of 18 features and for 16 out of 37 features after stratification by baseline age (Table S4).

### 3.3 Annotation of filtered features

The features prospectively associated with PCa risk that remained after the filtering process were annotated using accurate mass and MS^2^ data. For details of how the annotation of different features was supported by databases and MS^2^ (in both ionization modes), see Table S5. In the following, PCs are presented with the total number of carbons and double bonds in their side‐chain fatty acids and sphingomyelins (SMs) are presented with the total number of carbons and double bonds in their sphingosine backbone (ie, fatty acid plus sphingosine), unless detailed information on fatty acid composition was available. For details on assignment of individual side‐chain fatty acids and tentative assignment of their relative positions (when applicable), see Table S5.

Of the 50 unique features found to be prospectively associated with PCa risk, 18% were annotated at MSI level 2, 58% at level 3, and 24% at level 4. The parent ion *m/z* error of identification for all features was 2.69 ± 1.76 ppm (mean ± 95% CI).

### 3.4 Prospective association of filtered features with risk of prostate cancer

**FIGURE 2:**
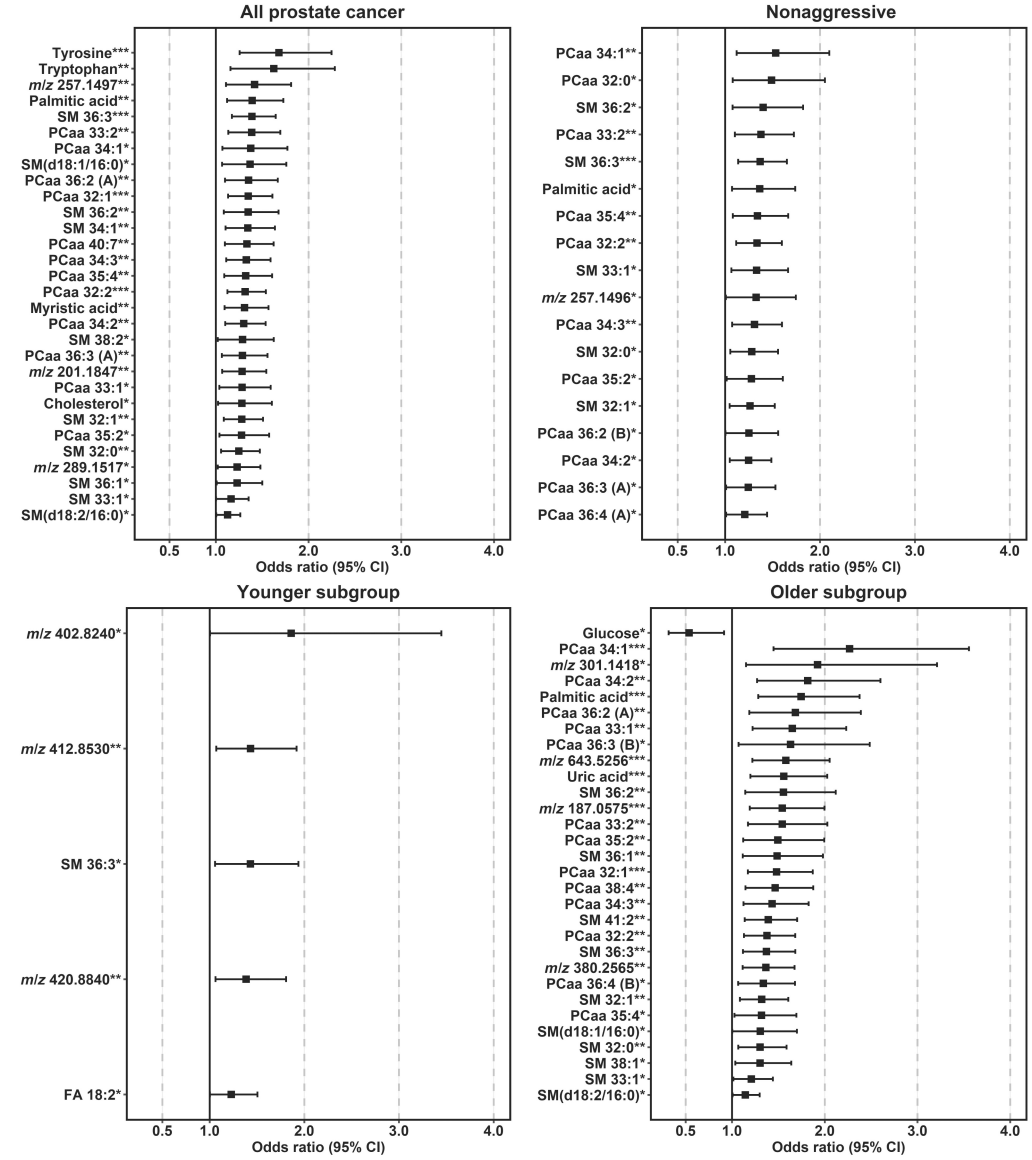
Odds ratios for prostate cancer (PCa) risk by log_2_ of metabolite feature signal intensities. The odds ratios and 95% confidence intervals (CIs) are derived from a conditional logistic regression, *P < .05, **P < .01, ***P < .001. The metabolites presented also showed variable importance on projection (VIP) ≥1.5 and VIP 95% CI >0 in orthogonal projection of latent structures‐effect projections (OPLS‐EP) models, and/or statistical significance (*P* < .05) after Bonferroni correction in a two‐sided Wilcoxon signed‐rank test. Adjustment for age, body mass index, smoking status, and alcohol consumption did not significantly affect the odds ratio. Upper left panel: results for all 752 matched case‐control pairs (overall), upper right panel: results for the nonaggressive subset (587 pairs), lower left panel: results for the younger subset (326 pairs) with a baseline age of 40 and 50 years, and lower right panel: results for the older subset (426 pairs) with a baseline age of 60 years. Two unannotated features (*m/z* 216.9227**), odds ratio (confidence interval) 5.49 (1.56‐19.3) and *m/z* 206.8938*, 3.13 (1.23‐7.96) were excluded from the younger subgroup plot, due to large CI (for details, see Table S6). Note the linear *x*‐axis. FA, fatty acid; PCaa, diacyl‐phosphatidylcholine; SM, sphingomyelin.

The odds ratios for the significantly associated features in the full dataset of PCa cases and the three subgroups (nonaggressive, younger, older) are graphically summarized in Figure 2. All features exhibiting a significant association with future PCa risk were positively associated with future disease development except for glucose, which was significantly negatively associated (odds ratio 0.54) for the older subgroup.

**FIGURE 3:**
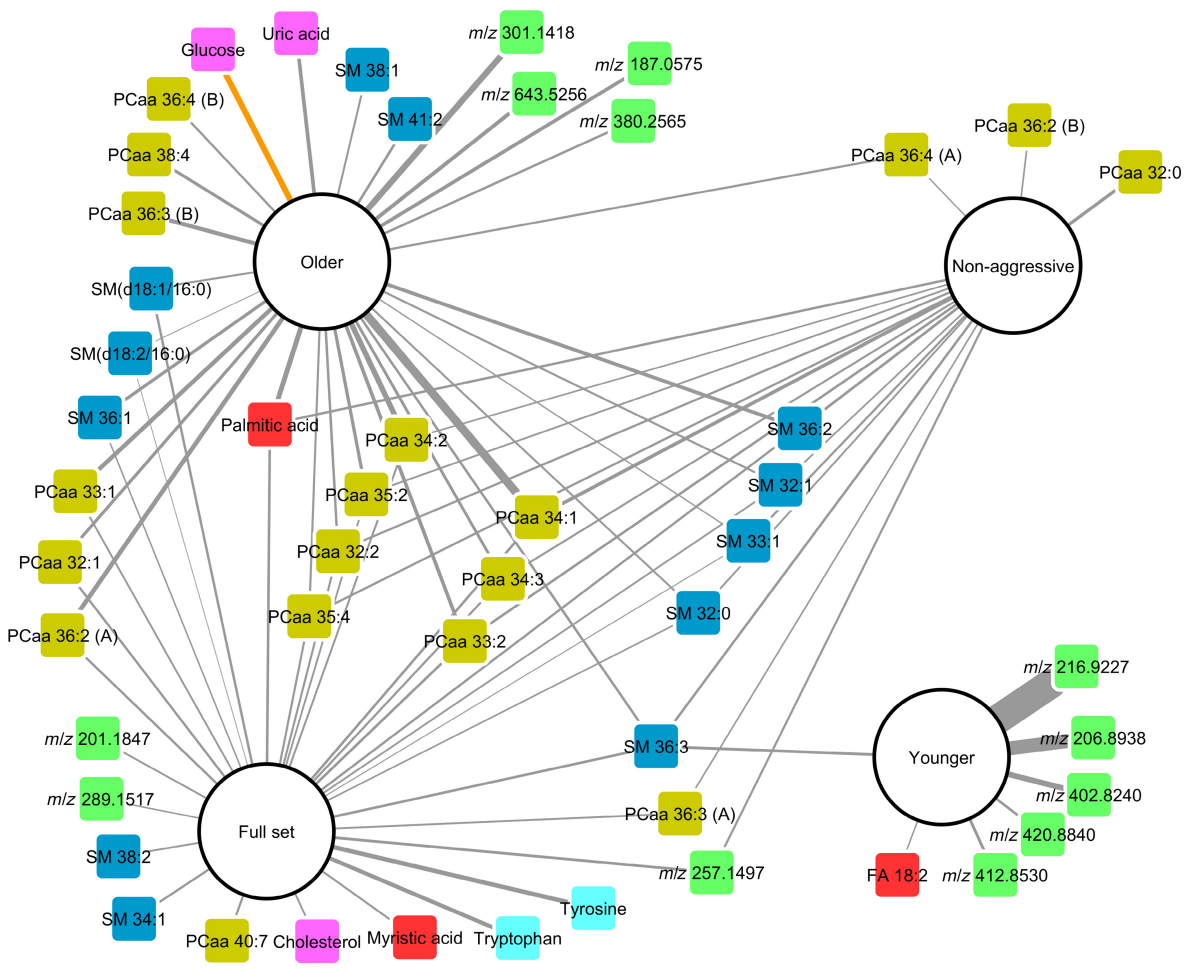
Network plot of filtered metabolite features prospectively associated with prostate cancer (PCa) risk for the full set of 752 case‐control pairs (overall) and after stratification by disease aggressiveness (587 nonaggressive pairs) or baseline age (326 younger pairs (40‐ and 50‐year‐olds) and 426 older pairs (60‐year‐olds)). The edge widths are weighted according to odds ratios by log_2_ of feature signal intensities. Edges with positive associations are shown in gray, while inverse associations are shown in orange. Model nodes are represented by white circles and feature nodes by squares colored by chemical class: Diacyl‐phosphatidylcholines (PCaas) (olive), sphingomyelins (SMs) (blue), amino acids (turquoise), fatty acids (red), others (purple), and unidentified (green).

Thirty features were positively associated with risk of overall PCa (full dataset of 752 pairs) including aromatic amino acids, phosphatidylcholines, sphingomyelins, free fatty acids, and cholesterol (the odds ratios ranged between 1.13 and 1.68). Eighteen features were positively associated with risk of nonaggressive PCa (dataset of 587 pairs) including phosphatidylcholines, sphingomyelins, and free fatty acids (the odds ratios ranged between 1.21 and 1.53). No significant association was found between metabolite features and risk of aggressive PCa (dataset of 165 pairs). Seven features were positively associated with risk of PCa in the younger subset of cases (dataset of 326 pairs) including (of those identified) a sphingomyelin and a free fatty acid (the odds ratios ranged between 1.23 and 5.49). Twenty‐nine features (including phosphatidylcholines, sphingomyelins, free fatty acids, and uric acid) were positively and one feature (glucose) was inversely associated with risk of PCa in the older subset of cases (data set of 426 pairs) (the odds ratios for positive associations ranged between 1.14 and 2.27). A summary of statistical and annotation data for all filtered features prospectively associated with PCa risk is provided in Table S6. A network plot of all 50 unique features showing features in common between the subsets and the full dataset is presented in Figure 3.

## 4 DISCUSSION

### 4.1 Main findings

In this nested case‐control study, untargeted high‐resolution mass spectrometry‐based metabolomics was employed to explore prospective associations of plasma metabolites with risk of PCa. Associations were also investigated after stratification of cases by baseline age and disease aggressiveness. A range of phospholipids, that is, diacyl‐phosphatidylcholines (PCaas) and SMs, and several free fatty acids were found to be positively associated with the risk of overall and nonaggressive PCa, and with the risk of PCa among younger and older subsets of cases. These phospholipids included both saturated and unsaturated odd‐ and even carbon fatty acid chains. Uric acid was found to be positively associated in the older subgroup, while glucose was found to be negatively associated.

### 4.2 Strengths and limitations

The present study is the largest prospective study (follow‐up 5‐20 years), which uses an untargeted LC‐MS metabolomics approach to look for circulating metabolites associated with risk of PCa. The prospective nature of study enables generating insight for better understanding PCa etiology, which paves the way for establishing prevention strategies. The use of an untargeted approach also expanded the number of metabolites investigated in association with risk of PCa. In addition, we only used overnight fasting plasma samples in our study, in order to reduce the variation in metabolite concentrations because of differences in recent foodstuff consumption and variations in time since last meal. We also used a follow‐up time of at least 5 years (follow‐up time ≥5 years) to reduce metabolic variations due to potential presence of subclinical PCa at baseline.

Our study had a number of limitations. The use of only untargeted LC‐MS, as compared to also using gas chromatography mass spectrometry metabolomics, limited the detection of some categories of metabolites. The study was also limited by the nonquantitative nature of untargeted LC‐MS metabolomics and the fact that some metabolite features remained unidentified even after MS^2^ analysis. In addition, only one blood sample was measured per participant. This can affect the results if a single measurement does not represent long‐term measurement. However, reasonable mid‐term (ie, a period of 4‐48 months) reproducibility of metabolite concentrations has been shown.^31^, ^32^ Moreover, our study was also limited by including participants only from a cohort of individuals from a single European country (ie, NSHDS, Sweden), not considering the effects of some other confounding factors in the model (ie, family history of PCa and physical activity) and low number of aggressive PCa cases.

## 5 OTHER STUDIES

Eleven other prospective studies (six different cohorts) investigating the association of metabolite levels with PCa risk have been published (referred to as “the 11 studies” hereafter).^5^, ^6^, ^7^, ^8^, ^9^, ^10^, ^11^, ^12^, ^13^, ^14^, ^16^ These studies differed in their experimental design and analytical methodologies. One study was nested in the Prostate, Lung, Colorectal and Ovarian Cancer Screening Trial (PLCO),^8^ one in the NSHDS,^10^ three in the Alpha‐Tocopherol, Beta‐Carotene Cancer Prevention Study (ATBC),^5^, ^6^, ^7^ three in the European Prospective Investigation into Cancer and Nutrition Study (EPIC),^9^, ^11^, ^12^ two in SU.VI.MAX,^13^, ^14^ and one in Cancer Prevention Study‐II Nutrition Cohort.^16^ Of the three EPIC studies, one was conducted within the EPIC‐Heidelberg cohort^9^ and two were overlapping multicenter studies.^11^, ^12^

Our previous study nested within the NSHDS^10^ and the three EPIC studies^9^, ^11^, ^12^ employed a targeted methodology, a kit‐based LC‐MS^2^ assay (Biocrates, Innsbruck, Austria). The three ATBC studies,^5^, ^6^, ^7^ one of the SU.VI.MAX studies,^14^ the PLCO study,^8^ and Cancer Prevention Study‐II Nutrition study^16^ employed an untargeted MS platform and included only identified metabolites prior to statistical analysis. No previous study employed the (untargeted) analytical methodology used in the present study, that is, LC‐high‐resolution MS with untargeted data processing using XCMS prior to the feature identification.

In our previous targeted MS analysis on the same nested cohort, we observed positive associations between several phospholipids, that is, acyl‐ether phosphatidylcholines (PCaes) and acyl‐lysophosphatidylcholines (LPCas), and risk of PCa.^10^ In the present study, we found positive associations with risk of PCa for several PCs and SMs, but these were not the same PCs as in our previous study. We previously observed a positive association between LPCa 17:0 and risk of overall and aggressive PCa for different age groups,^10^ but LPCa 17:0 was not among the filtered features found in the present study. However, we observed associations between several other phospholipids with odd‐chain fatty acids in their structure and risk of PCa, that is, PCaa 33:2, PCaa 35:2, PCaa 35:4, PCaa 33:1, SM 33:1 and SM 41:2.

Differences between our present and previous study can be partly attributed to differences in the analytical procedures used, including: (a) lower sensitivity of the QToF method used in the present study compared to the triple quadrupole method used in our previous study (ie, relevant also for LPCa 17:0); (b) none of the PCs and SMs with odd‐chain fatty acids among the filtered features in the present untargeted metabolomics study were included in the list of metabolites measured by the targeted methodology used in our previous study,^10^ and (c) the untargeted and targeted methods used in the present and previous study, respectively, differed in their analytical selectivity due to differences between the present LC‐high‐resolution MS approach and the previous flow injection analysis‐low‐resolution triple quadrupole approach.^33^ To elaborate, in a low‐resolution triple quadrupole approach, the feature annotated as, for example, PCaa 33:1 in the present study might potentially overlap with what is reported as PCae 34:1 (36 mDa mass difference).

In the larger EPIC study^11^ including the case‐control sets from the smaller EPIC study,^12^ none of multivariate components representing different groups of metabolites was associated with overall, localized, nonaggressive, low‐intermediate or high‐grade PCa, according to the authors. However, that study^11^ found inverse associations between components representing phospholipids and risk of advanced stage PCa. In the present study, we found positive associations between several phospholipids and risk of overall and nonaggressive PCa. However, we did not observe any association between metabolites and risk of aggressive PCa (possibly because of small sample size). The experimental design in the present study differed from that in EPIC multicenter studies by using (a) only overnight fasting plasma samples (compared to also including nonfasting in EPIC multicenter studies) and (b) inclusion of both nonlocalized (T3‐4/N1‐3/M1) and high‐grade (Gleason sum score ≥8) tumors in aggressive disease subtype as in some previous studies^5^, ^6^, ^8^ (compared to only nonlocalized in advanced stage subtype in EPIC multicenter studies). These differences might partly explain the differences in the observed associations.

None of the PCs reported in the PLCO and ATBC cohort studies were found to be significant after correction for multiple testing,^5^, ^7^, ^8^ but positive associations between PCaa 34:3 and risk of nonaggressive PCa were observed in both the present study and the PLCO study.^8^ PCaa 36:2 (A) and PCaa 34:2, which were positively associated with overall PCa risk in the present study, showed an inverse association (*P* < .05) with overall and T3 PCa in the ATBC studies.^5^, ^7^ One of the ATBC studies found a positive association (although not after correction for multiple testing) between SM(d18:1/18:0) and risk of T3 PCa.^7^ This SM was possibly found for the full set and older subgroup in the present study, in the form of SM 36:1. No associations were found for any sphingomyelins in the other four previous studies.^6^, ^8^, ^9^, ^10^

In the present study, tyrosine and tryptophan were the two features exhibiting the highest odds ratios in the full dataset. Positive associations between tyrosine and risk of overall PCa have been reported previously in one SU.VI.MAX study^13^ and inverse associations between tryptophan (not after correction for multiple testing) and risk of overall and aggressive PCa in the PLCO study.^34^

In the present study, palmitic acid was found to have a significant positive association with future PCa risk for both the full set (overall) and the nonaggressive and older subgroups. Palmitic acid was not found to be significantly associated with PCa risk after correction for multiple testing in any of the previous 11 studies, but inverse associations were found for both nonaggressive and aggressive PCa in the ATBC cohort.^5^, ^6^ In the present study, several of the annotated phospholipids associated with future PCa risk contained palmitate side‐chains (Tables S5 and S6). Previous studies investigating the fatty acid composition of plasma phospholipids have consistently reported positive associations between higher amounts of palmitate in phospholipids and risk of overall PCa,^35^, ^36^ and as part of a pattern of three fatty acids in phospholipids positively associated with risk of higher‐grade PCa (ie, Gleason sum score ≥ 7).^37^

Uric acid was found to be positively associated with future PCa risk in the older subgroup in our study, but no such association was found in any of the 11 previous (metabolomics) studies. Other previous studies (those not using metabolomics analysis) have presented conflicting results regarding uric acid and risk of PCa. A positive association with PCa risk has been found in a prospective cohort of Japanese men in Hawaii,^38^ while some studies show no association.^39^, ^40^ A large Austrian study found higher uric acid to be associated with overall cancer, but not with genital cancers (including PCa).^41^ Interestingly, elevated uric acid has previously been shown to be associated with PCa risk in a smaller Swedish study.^42^

The inverse association between glucose and PCa risk in the present study (using an untargeted approach) is consistent with the inverse association found between impaired glucose tolerance and risk of PCa in our previous study^10^ and lower risk of subsequently developing PCa in patients with type 2 diabetes (T2D).^43^, ^44^, ^45^

## 6 SUGGESTED MECHANISM

In the present study, plasma lipids (ie, phospholipids and free fatty acids) and uric acid positively, and plasma glucose inversely, were associated with risk of PCa. It is important to investigate if these metabolic alterations can lead the research to upstream gene and signaling pathways with significance in etiology of PCa. Here, we suggest a common upstream signaling pathway, with potential implications in risk of PCa, may have contributed to the metabolic alterations observed in the present study.

Uric acid is a byproduct of purine catabolism. Although excess cancer risk in association with higher concentration of uric acid has been attributed to induction of proinflammatory responses, no significant causality between serum uric acid and incidence of several distinct malignancies, that is, PCa has been evidenced in Mendelian randomization studies.^46^ Blood concentration of uric acid is determined by endogenous production and urinary excretion. Intriguingly, it has been shown that the reabsorption of uric acid in kidneys, which results in its reduced urinary expression, is increased by activation of PI3K‐Akt signaling pathway.^47^ This pathway is also a very important signaling pathway for regulating glucose homeostasis and its activation can reduce circulating glucose concentration.^48^ A relatively higher activity of PI3K‐Akt pathway in cases compared to controls can potentially induce a metabolic phenotype similar with that observed in the present study, that is, higher lipids (possibly because of higher endogenous biosynthesis) and uric acids (because of lower urinary excretion) and lower glucose in fasting plasma. Higher activity of PI3K‐Akt singling is also etiologically important in the development of PCa.^49^ Therefore, here we propose a prospectively higher activity of PI3K‐Akt singling in PCa cases compared to controls. PI3K‐Akt activity is reduced by phosphatase and tensin homolog (*PTEN*),^48^ a tumor suppressor protein also involved in PCa development^49^ and glucose metabolism.^48^ Intriguingly, PTEN can be measured in prospective plasma as an important regulator of PI3K‐Akt singling pathway.^48^

## 7 CONCLUSIONS

The present study, which is the largest prospective untargeted metabolomics study to date focusing on PCa risk, identified new metabolic PCa risk factors observable in blood plasma at least 5 years before diagnosis. Positive associations with PCa risk were found for aromatic amino acids, free fatty acids, uric acid, and a range of phospholipids, while inverse associations were found for glucose. The differential associations were observed based on stratification of cases by baseline age (eg, for glucose and uric acid), highlighting the importance of considering subtyping in investigations of PCa etiology and its underlying causes. These findings suggest a possible role for lipid and glucose metabolism in PCa etiology. Future studies are needed on the origins of these new risk factors and whether they are adjustable or genetic, using research material with a higher number or proportion of aggressive cases. Understanding the origins of these risk factors will open new doors for establishing prevention strategies.^50^

## AUTHOR CONTRIBUTIONS

**Johnny R. Östman:** Metabolomics analysis, data analysis and drafted the article. **Rui C. Pinto:** Data processing. **Timothy M. D. Ebbels:** Data processing. **Elin Thysell:** Data evaluation and reviewed the article. **Göran Hallmans:** Data evaluation and reviewed the article. **Ali A. Moazzami:** Conceived and designed the study, received funding, participated in the interpretation of findings and supervised data analysis and article writing.

## CONFLICT OF INTEREST

The authors declare no potential conflicts of interest.

## ETHICS STATEMENT

The Research Ethics Committee of Umeå University Hospital and the Regional Ethical Committee in Uppsala (no. 2013‐124) approved our study. All participants gave informed consent to participate in the study.

## Supporting information


**Appendix S1**Supporting Information.

## Data Availability

To access the data and samples for another study, after an ethical approval, a request should be submitted to the Biobank Research Unit at Umeå University, which administers and conveys information about access to data and samples in our study (https://www.umu.se/en/biobank-research-unit/). Further information is available from the corresponding author upon request.

## References

[1] Bray F , Ferlay J , Soerjomataram I , Siegel RL , Torre LA , Jemal A . Global cancer statistics 2018: GLOBOCAN estimates of incidence and mortality worldwide for 36 cancers in 185 countries. CA Cancer J Clin. 2018;68:394‐424. 30207593 10.3322/caac.21492

[2] Pernar CH , Ebot EM , Wilson KM , Mucci LA . The epidemiology of prostate cancer. Cold Spring Harb Perspect Med. 2018;8:a030361. 29311132 10.1101/cshperspect.a030361 PMC6280714

[3] World Cancer Research Fund/American Institute for Cancer Research, Diet, Nutrition, Physical Activity and Prostate Cancer: A gloBal Perspective. Continuous Update Project Expert Report; 2018.

[4] Nicholson JK , Holmes E , Elliott P . The metabolome‐wide association study: a new look at human disease risk factors. J Proteome Res. 2008;7:3637‐3638. 18707153 10.1021/pr8005099

[5] Mondul AM , Moore SC , Weinstein SJ , Karoly ED , Sampson JN , Albanes D . Metabolomic analysis of prostate cancer risk in a prospective cohort: the alpha‐tocolpherol, beta‐carotene cancer prevention (ATBC) study. Int J Cancer. 2015;137:2124‐2132. 25904191 10.1002/ijc.29576 PMC4537663

[6] Mondul AM , Moore SC , Weinstein SJ , Männistö S , Sampson JN , Albanes D . 1‐stearoylglycerol is associated with risk of prostate cancer: results from serum metabolomic profiling. Metabolomics. 2014;10:1036‐1041. 25254003 10.1007/s11306-014-0643-0 PMC4169990

[7] Huang J , Mondul AM , Weinstein SJ , Karoly ED , Sampson JN , Albanes D . Prospective serum metabolomic profile of prostate cancer by size and extent of primary tumor. Oncotarget. 2017;8:45190‐45199. 28423352 10.18632/oncotarget.16775 PMC5542177

[8] Huang J , Mondul AM , Weinstein SJ , et al. Serum metabolomic profiling of prostate cancer risk in the prostate, lung, colorectal, and ovarian cancer screening trial. Br J Cancer. 2016;115:1087‐1095. 27673363 10.1038/bjc.2016.305 PMC5117796

[9] Kühn T , Floegel A , Sookthai D , et al. Higher plasma levels of lysophosphatidylcholine 18:0 are related to a lower risk of common cancers in a prospective metabolomics study. BMC Med. 2016;14:13. 26817443 10.1186/s12916-016-0552-3 PMC4730724

[10] Röhnisch HE , Kyrø C , Olsen A , Thysell E , Hallmans G , Moazzami AA . Identification of metabolites associated with prostate cancer risk: a nested case‐control study with long follow‐up in the northern Sweden Health and Disease Study. BMC Med. 2020;18:187. 32698845 10.1186/s12916-020-01655-1 PMC7376662

[11] Schmidt JA , Fensom GK , Rinaldi S , et al. Patterns in metabolite profile are associated with risk of more aggressive prostate cancer: a prospective study of 3,057 matched case‐control sets from EPIC. Int J Cancer. 2020;146:720‐730. 30951192 10.1002/ijc.32314 PMC6916595

[12] Schmidt JA , Fensom GK , Rinaldi S , et al. Pre‐diagnostic metabolite concentrations and prostate cancer risk in 1077 cases and 1077 matched controls in the European Prospective Investigation into Cancer and Nutrition. BMC Med. 2017;15:122. 28676103 10.1186/s12916-017-0885-6 PMC5497352

[13] Lecuyer L , Victor Bala A , Demidem A , et al. NMR metabolomic profiles associated with long‐term risk of prostate cancer. Metabolomics. 2021;17:32. 33704614 10.1007/s11306-021-01780-9

[14] Lin X , Lecuyer L , Liu X , et al. Plasma metabolomics for discovery of early metabolic markers of prostate cancer based on ultra‐high‐performance liquid chromatography‐high resolution mass spectrometry. Cancers (Basel). 2021;13:3140. 10.3390/cancers13133140 PMC8268247 34201735

[15] Huang J , Weinstein SJ , Moore SC , et al. Pre‐diagnostic serum metabolomic profiling of prostate cancer survival. J Gerontol A Biol Sci Med Sci. 2019;74:853‐859. 29878065 10.1093/gerona/gly128 PMC6521920

[16] Wang Y , Jacobs EJ , Carter BD , Gapstur SM , Stevens VL . Plasma metabolomic profiles and risk of advanced and fatal prostate cancer. Eur Urol Oncol. 2021;4:56‐65. 31378665 10.1016/j.euo.2019.07.005

[17] Huang J , Mondul AM , Weinstein SJ , et al. Prospective serum metabolomic profiling of lethal prostate cancer. Int J Cancer. 2019;145:3231‐3243. 30779128 10.1002/ijc.32218 PMC6698432

[18] Krug S , Kastenmüller G , Stückler F , et al. The dynamic range of the human metabolome revealed by challenges. FASEB J. 2012;26:2607‐2619. 22426117 10.1096/fj.11-198093

[19] Shrestha A , Müllner E , Poutanen K , Mykkänen H , Moazzami AA . Metabolic changes in serum metabolome in response to a meal. Eur J Nutr. 2017;56:671‐681. 26658764 10.1007/s00394-015-1111-y

[20] Hallmans G , Ågren Å , Johansson G , et al. Cardiovascular disease and diabetes in the northern Sweden Health and Disease Study Cohort: evaluation of risk factors and their interactions. Scand J Public Health. 2003;31:18‐24. 10.1080/14034950310001432 14660243

[21] Stattin P , Rinaldi S , Biessy C , Stenman U‐H , Hallmans G , Kaaks R . High levels of circulating insulin‐like growth factor‐I increase prostate cancer risk: a prospective study in a population‐based nonscreened cohort. J Clin Oncol. 2004;22:3104‐3112. 15284261 10.1200/JCO.2004.10.105

[22] Mostofi FK , Sesterhenn IA , Sobin LH . Histological Typing of Prostate Tumours. Geneva, Switzerland: World Health Organization; 1980:17‐21.

[23] Egevad L . Reproducibility of Gleason grading of prostate cancer can be improved by the use of reference images. Urology. 2001;57:291‐295. 11182339 10.1016/s0090-4295(00)00922-5

[24] Evans AM , DeHaven CD , Barrett T , Mitchell M , Milgram E . Integrated, nontargeted ultrahigh performance liquid chromatography/electrospray ionization tandem mass spectrometry platform for the identification and relative quantification of the small‐molecule complement of biological systems. Anal Chem. 2009;81:6656‐6667. 19624122 10.1021/ac901536h

[25] Jonsson P , Wuolikainen A , Thysell E , et al. Constrained randomization and multivariate effect projections improve information extraction and biomarker pattern discovery in metabolomics studies involving dependent samples. Metabolomics. 2015;11:1667‐1678. 26491420 10.1007/s11306-015-0818-3 PMC4605978

[26] Smith CA , Want EJ , O'Maille G , Abagyan R , Siuzdak G . XCMS: processing mass spectrometry data for metabolite profiling using nonlinear peak alignment, matching, and identification. Anal Chem. 2006;78:779‐787. 16448051 10.1021/ac051437y

[27] Climaco Pinto R , Karaman I , Lewis MR , et al. Finding correspondence between metabolomic features in untargeted liquid chromatography‐mass spectrometry metabolomics datasets. Anal Chem. 2022;94:5493‐5503. 35360896 10.1021/acs.analchem.1c03592 PMC9008693

[28] Eriksson L , Trygg J , Wold S . CV‐ANOVA for significance testing of PLS and OPLS® models. J Chemometr. 2008;22:594‐600.

[29] Favilla S , Durante C , Vigni ML , Cocchi M . Assessing feature relevance in NPLS models by VIP. Chemometr Intell Lab. 2013;129:76‐86.

[30] Wolfer A , Correia G . peakPantheR: Peak Picking and Annotation of High Resolution Experiments 2020.

[31] Floegel A , Drogan D , Wang‐Sattler R , et al. Reliability of serum metabolite concentrations over a 4‐month period using a targeted metabolomic approach. PLoS One. 2011;6:e21103. 21698256 10.1371/journal.pone.0021103 PMC3115978

[32] Carayol M , Licaj I , Achaintre D , et al. Reliability of serum metabolites over a two‐year period: a targeted Metabolomic approach in fasting and non‐fasting Samples from EPIC. PLoS One. 2015;10:e0135437. 26274920 10.1371/journal.pone.0135437 PMC4537119

[33] Quell JD , Römisch‐Margl W , Haid M , et al. Characterization of bulk phosphatidylcholine compositions in human plasma using side‐chain resolving lipidomics. Metabolites. 2019;9:109. 31181753 10.3390/metabo9060109 PMC6631474

[34] Khan A , Choi SA , Na J , et al. Noninvasive serum metabolomic profiling reveals elevated kynurenine pathway's metabolites in humans with prostate cancer. J Proteome Res. 2019;18:1532‐1541. 30628444 10.1021/acs.jproteome.8b00803

[35] Harvei S , Bjerve KS , Tretli S , Jellum E , Robsahm TE , Vatten L . Prediagnostic level of fatty acids in serum phospholipids: Ω‐3 and Ω‐6 fatty acids and the risk of prostate cancer. Int J Cancer. 1997;71:545‐551. 9178806 10.1002/(sici)1097-0215(19970516)71:4<545::aid-ijc7>3.0.co;2-u

[36] Crowe FL , Allen NE , Appleby PN , et al. Fatty acid composition of plasma phospholipids and risk of prostate cancer in a case‐control analysis nested within the European Prospective Investigation into Cancer and Nutrition. Am J Clin Nutr. 2008;88:1353‐1363. 18996872 10.3945/ajcn.2008.26369

[37] Dahm CC , Gorst‐Rasmussen A , Crowe FL , et al. Fatty acid patterns and risk of prostate cancer in a case‐control study nested within the European Prospective Investigation into Cancer and Nutrition. Am J Clin Nutr. 2012;96:1354‐1361. 23134890 10.3945/ajcn.112.034157

[38] Kolonel LN , Yoshizawa C , Nomura AM , Stemmermann GN . Relationship of serum uric acid to cancer occurrence in a prospective male cohort. Cancer Epidemiol Biomarkers Prev. 1994;3:225‐228. 8019371

[39] Wang A , Barber JR , Tin A , et al. Serum urate, genetic variation, and prostate cancer risk: atherosclerosis risk in communities (ARIC) study. Cancer Epidemiol Biomarkers Prev. 2019;28:1259‐1261. 31263056 10.1158/1055-9965.EPI-19-0161 PMC6608724

[40] Kühn T , Sookthai D , Graf ME , et al. Albumin, bilirubin, uric acid and cancer risk: results from a prospective population‐based study. Br J Cancer. 2017;117:1572‐1579. 28898231 10.1038/bjc.2017.313 PMC5680462

[41] Strasak AM , Lang S , Kneib T , et al. Use of penalized splines in extended cox‐type additive Hazard regression to flexibly estimate the effect of time‐varying serum uric acid on risk of cancer incidence: a prospective, population‐based study in 78,850 men. Ann Epidemiol. 2009;19:15‐24. 18835524 10.1016/j.annepidem.2008.08.009 PMC2666912

[42] Hammarsten J , Högstedt B . Clinical, haemodynamic, anthropometric, metabolic and insulin profile of men with high‐stage and high‐grade clinical prostate cancer. Blood Press. 2004;13:47‐55. 15083641 10.1080/08037050310025735

[43] Kasper JS , Liu Y , Giovannucci E . Diabetes mellitus and risk of prostate cancer in the health professionals follow‐up study. Int J Cancer. 2009;124:1398‐1403. 19058180 10.1002/ijc.24044 PMC2680190

[44] Tsilidis KK , Allen NE , Appleby PN , et al. Diabetes mellitus and risk of prostate cancer in the European prospective investigation into cancer and nutrition. Int J Cancer. 2015;136:372‐381. 24862312 10.1002/ijc.28989

[45] Feng X , Song M , Preston MA , et al. The association of diabetes with risk of prostate cancer defined by clinical and molecular features. Br J Cancer. 2020;123:657‐665. 32467600 10.1038/s41416-020-0910-y PMC7435261

[46] Jiang M , Ren L , Chen S , Li G . Serum uric acid levels and risk of eight site‐specific cancers: a Mendelian randomization study. Front Genet. 2021;12:608311. 33767728 10.3389/fgene.2021.608311 PMC7985250

[47] Mandal AK , Leask MP , Estiverne C , Choi HK , Merriman TR , Mount DB . Genetic and physiological effects of insulin on human urate homeostasis. Front Physiol. 2021;12:713710. 34408667 10.3389/fphys.2021.713710 PMC8366499

[48] Li YZ , Di Cristofano A , Woo M . Metabolic role of PTEN in insulin signaling and resistance. Cold Spring Harb Perspect Med. 2020;10:a036137. 31964643 10.1101/cshperspect.a036137 PMC7397839

[49] Pencik J , Schlederer M , Gruber W , et al. STAT3 regulated ARF expression suppresses prostate cancer metastasis. Nat Commun. 2015;6:7736. 26198641 10.1038/ncomms8736 PMC4525303

[50] Östman J . [Doctoral thesis] Metabolomics and flux analysis by mass spectrometry : Investigations of factors associated with insulin secretion and prostate cancer risk. Acta Universitatis Agriculturae Sueciae; 2021:18.

