# Supplementary material for: Identification of prediagnostic metabolites associated with prostate cancer risk by untargeted mass spectrometry‐based metabolomics: A case‐control study nested in the Northern Sweden Health and Disease Study
Source: Int J Cancer. 2022 Aug 12;151(12):2115–27. doi: 10.1002/ijc.34223 (PMC9804595; doi:10.1002/ijc.34223)
Supplement: Supplementary file 1 — Appendix S1Supporting Information. [file IJC-151-2115-s001.pdf]

Supporting Information

Identification of pre-diagnostic metabolites associated with prostate cancer risk by untargeted mass spectrometry-based metabolomics

A case-control study nested in the Northern Sweden Health and Disease Study

Johnny R Östman, Rui C Pinto, Timothy MD Ebbels, Elin Thysell, Göran Hallmans, and Ali A Moazzami

Table of contents	1
Supplementary Materials & Methods	2-4
Supplementary Table 1. *Compilation of metabolite feature filtering procedure*	5–16
Supplementary Table 2. *Association between discriminating features identified by orthogonal projection of latent structures-effect projections (OPLS-EP) and prostate cancer risk*	17–27
Supplementary Table 3. *Association between discriminating features identified by Wilcoxon signed-rank tests and prostate cancer risk*	28–30
Supplementary Table 4. *Differential associations by age group and disease aggressiveness*	31–33
Supplementary Table 5. *MS^2 data and annotation of filtered features associated with prostate cancer risk*	34–41
Supplementary Table 6. *Compilation of data for filtered features associated with prostate cancer risk*	42–45
Supplementary Figure 1. Identification of discriminative features	46

Supplementary Materials & Methods

Liquid chromatography-mass spectrometry

The samples were prepared according to the method described by Evans *et al.* [1] with minor modifications. A 50 µL aliquot of plasma was mixed with 200 µL of methanol (LC-MS grade, JT Baker, Phillipsburg, NJ) with temperature -20 °C, vortexed, incubated at -20 °C for 20 min, and centrifuged (16,000 g for 15 min). The supernatant was split between two tubes (90+90 µL), for analysis in either positive or negative ionization mode. The samples were dried down in a speed vacuum concentrator overnight at 30 °C. The aliquots were reconstituted in 50 µL aqueous solution of 0.1% formic acid (vol:vol) (LC-MS grade, Fisher Chemical, Pittsburgh, PA) or 6.5 mM ammonium bicarbonate (LC-MS grade, Honeywell Fluka, Charlotte, NC) for positive or negative ionization mode analysis, respectively. External heparinized quality control (QC) samples were prepared in the same manner for the respective modes and were injected after every eighth sample, in order to monitor instrument drift. The two paired samples were analyzed immediately after each other, with case-control injection order randomized in order to minimize impact of instrument drift [2]. Due to logistical limitations, the samples were analyzed in four batches in both positive and negative mode.

Chromatographic separation was carried out on MS-analysis polarity dedicated Waters BEH C18 (1.7 µm, 2.1 × 100 mm) columns (Waters, Milford, MA), using an Agilent 1290 Infinity II Ultrahigh Performance Liquid Chromatograph (Agilent Technologies, Santa Clara, CA). The injection volume was 5 µL, the column was kept at 60 °C, and the flow rate was 350 µL/min for both polarities. For positive mode separation, (A) 0.1% formic acid in water and (B) 0.1% formic acid in methanol were used as eluents. For negative mode separation, (A) 6.5 mM ammonium bicarbonate in water and (B) 6.5 mM ammonium bicarbonate in methanol (ammonium bicarbonate soluble upon sonication) were used as eluents. The following gradient profile was used in both modes: 2% B to 70% B in 4 min, 70-98% B in 1.5 min, 98% B for 2.9 min, 2% B in 1 min, 2% B for 2.6 min.

Mass spectrometry analyses were carried out on an electrospray quadrupole-time-of-flight (QToF) mass spectrometer (Bruker maXis impact, Bruker Daltonics, Bremen, Germany) with a plate offset voltage of ±500 V and capillary voltage set at ±4 kV. Nitrogen gas at a temperature of 200 °C was administered at 8 L/min with a nebulizer pressure of 2 bar for desolvation. Positive and negative ionization mode profile sample spectra in the range 50-1200 *m/z* were collected at a rate of 5 Hz, with the digitizer sample rate set at 4 GHz. MS^2 spectra were also collected, in order to assist in annotation of metabolite features found to be significantly associated with PCa risk after statistical analysis and feature filtering. Positive and negative ionization mode collisional induced dissociation (CID) fragment ion spectra (MS^2) were collected for all filtered features, irrespective of the chromatographic conditions in which the feature was first detected. For the MS^2 analyses, a mixture of 100 plasma samples picked randomly from among the 1504 study samples was prepared. The sample mixture was extracted and separated under the same two sets of chromatographic conditions as described above. The $[M+H]^+$ and $[M-H]^-$-adducts of all filtered features were individually isolated (isolation width 0.5 *m/z*) and fragmented.

Data processing

The liquid chromatography-mass spectrometry (LC-MS) profile spectra raw data were converted to centroided mzML format using Bruker CompassXport (v. 3.0.9.2) and processed using the R (https://www.r-project.org/, v. 3.5.1) package *XCMS* (v. 3.4.2) [3]. Data processing included peak picking, grouping, and filling of zero intensity features. Peak picking was performed using the 'centWave' algorithm (positive/negative mode settings: peak width = 5-30/4-25 s, S/N threshold = 3, ppm = 30/40), grouping was carried out using the 'density' algorithm (bw

= 10), and filling of zero intensity features was performed using the 'fillPeaks' algorithm with the 'chrom' method. The processing resulted in a peak table in which the spectral features in each sample were assigned an *m/z*-value, a retention time value, and an intensity value. Data processing was applied separately on the four batches for each polarity.

An in-house algorithm [4] was used to find correspondence between (metabolite) features in the different batches. The procedure was defined as follows: (1) All possible matches within adequately defined median retention time (RT) and *m/z*-thresholds were identified batch-wise. This resulted in clusters, including features from the four batches, and in inter-batch alignment of both RT and *m/z*. (2) In each cluster, only features that matched features in all other batches were allowed (*i.e.*, cliques), while the rest were deleted; (3) In cases of match multiplicity, a penalization score was created to decide the best match. This score was defined after batch-to-batch alignment as the Euclidean distance of RT and *m/z* difference (both normalized) between each of the features in the match. Processing was performed separately on the positive and negative mode datasets. The two resulting datasets (one for the four batches analyzed in positive mode and one for the four batches analyzed in negative mode) were then merged, resulting in a single dataset containing the 1100 metabolite features in total detected in all four positive-mode and four negative-mode batches. The data were not adjusted for intra-batch instrument drift, since all statistical comparisons were made pair-wise, with the two-paired samples being analyzed consecutively in randomized order [2].

Annotation of filtered metabolite features

Metabolite feature annotation was based on accurate mass of the parent ion and accurate masses of the CID fragments in both positive and negative mode (when applicable). The accurate masses presented for the parent ions used for annotation are manually curated *m/z*-values from a randomly chosen subset of raw profile mass spectra, whereas the values provided by XCMS are based on centroided spectra. Comparisons of spectral data with databases (Human Metabolome Database (HMDB), METLIN, mzCloud, Lipid Maps, MassBank) were conducted in order to aid annotation. Annotation of phospholipid features was also assisted by previously presented guidelines consisting of lists of ions characteristic for phospholipid class identification for different adducts and ionization modes, common fragmentation patterns, and lists of fragments characteristic for specific side-chains [5]. When two different filtered features with the same sum of side-chain carbons and unsaturations were annotated, they were given an additional (A) or (B) suffix.

The annotation of individual side-chain fatty acids was determined based on the accurate masses of detected free fatty acid, lysophosphatidylcholine, or lysosphingomyelin CID fragments (when applicable). The relative positions of side-chain fatty acids in phosphatidylcholines (PCs) were suggested (when applicable) based on CID fragment signal intensities, with the stronger of the two potential lyso-fragment signals corresponding to the ion with a fatty acid on carbon 3/*sn*-1. Secondary carbocations formed by loss of the side-chain at the *sn*-2 position of the glycerol backbone are more stable than primary carbocations formed by loss at *sn*-1, yielding higher signals for lyso-fragment signals with a fatty acid on carbon 3/*sn*-1. The level of identification presented follows the Metabolomics Standards Initiative (MSI) guidelines for metabolite identification [6].

References

1. Evans AM, DeHaven CD, Barrett T, Mitchell M, Milgram E. Integrated, nontargeted ultrahigh performance liquid chromatography/electrospray ionization tandem mass spectrometry platform for the identification and relative quantification of the small-molecule complement of biological systems. *Analytical Chemistry* 2009;**81**: 6656-67.

2. Jonsson P, Wuolikainen A, Thysell E, Chorell E, Stattin P, Wikstrom P, Antti H. Constrained randomization and multivariate effect projections improve information extraction and biomarker pattern discovery in metabolomics studies involving dependent samples. *Metabolomics* 2015;**11**: 1667-78.

3. Smith CA, Want EJ, O'Maille G, Abagyan R, Siuzdak G. XCMS: Processing Mass Spectrometry Data for Metabolite Profiling Using Nonlinear Peak Alignment, Matching, and Identification. *Analytical Chemistry* 2006;**78**: 779-87.

4. Climaco Pinto R, Karaman I, Lewis MR, Hallqvist J, Kaluarachchi M, Graca G, Chekmeneva E, Durainayagam B, Ghanbari M, Ikram MA, Zetterberg H, Griffin J, et al. Finding Correspondence between Metabolomic Features in Untargeted Liquid Chromatography-Mass Spectrometry Metabolomics Datasets. *Anal Chem* 2022;**94**: 5493-503.

5. Godzien J, Ciborowski M, Martínez-Alcázar MP, Samczuk P, Kretowski A, Barbas C. Rapid and Reliable Identification of Phospholipids for Untargeted Metabolomics with LC-ESI-QTOF-MS/MS. *Journal of proteome research* 2015;**14**: 3204-16.

6. Sumner LW, Amberg A, Barrett D, Beale MH, Beger R, Daykin CA, Fan TW, Fiehn O, Goodacre R, Griffin JL, Hankemeier T, Hardy N, et al. Proposed minimum reporting standards for chemical analysis Chemical Analysis Working Group (CAWG) Metabolomics Standards Initiative (MSI). *Metabolomics : Official journal of the Metabolomic Society* 2007;**3**: 211-21.

Supplementary Table 1. *Compilation of metabolite feature filtering procedure*

**Feature**			**Overall prostate cancer (752 pairs)**			
**Polarity**	***m/z***	**RT (min)**	**Statistical procedure**	**Filtered, yes/no**	**Filtering step**	**Adduct/isotope detail**
Neg	311.2002	6.16	M	Yes	CLR	-
Neg	356.1849	6.23	M	Yes	Visual inspection	-
Neg	367.2623	6.54	M	Yes	CLR	-
Neg	368.2656	6.53	MU	Yes	Covar	-
Neg	381.3714	6.79	M	Yes	Visual inspection	-
Neg	395.3870	6.90	M	Yes	Visual inspection	-
Neg	409.2343	6.40	M	Yes	CLR	-
Neg	410.2372	6.40	M	Yes	CLR	-
Neg	412.2472	5.95	M	Yes	CLR	-
Neg	425.3611	5.97	M	Yes	Visual inspection	-
Neg	480.3061	6.40	M	Yes	CLR	-
Neg	508.3365	6.58	M	Yes	CLR	-
Neg	540.3273	6.40	M	Yes	CLR	-
Neg	541.3305	6.40	M	Yes	CLR	-
Neg	566.3424	6.45	M	Yes	CLR	-
Neg	748.5213	7.45	M	Yes	CLR	-
Pos	189.0715	2.24	U	No	-	-
Pos	201.1828	6.25	M	No	-	-
Pos	205.0836	5.96	M	Yes	CLR	-
Pos	226.0424	1.16	U	No	-	-
Pos	227.0764	2.25	U	Yes	Adduct/isotope	Adduct of 189
Pos	229.2139	6.50	MU	No	-	-
Pos	239.2345	6.69	M	Yes	Adduct/isotope	M-H_2O of 257
Pos	257.1486	6.11	MU	No	-	-
Pos	257.2450	6.69	MU	No	-	-
Pos	258.2480	6.69	MU	Yes	Adduct/isotope	M+1 of 257
Pos	289.1510	5.92	U	No	-	-
Pos	301.1381	5.95	M	Yes	CLR	-
Pos	302.1413	5.95	M	Yes	CLR	-
Pos	315.1900	6.09	M	Yes	CLR	-
Pos	326.2420	5.92	U	Yes	Visual inspection	-
Pos	357.2457	7.24	M	Yes	CLR	-
Pos	369.3478	7.70	MU	No	-	-
Pos	370.2526	7.30	M	Yes	CLR	-
Pos	371.2604	7.51	M	Yes	CLR	-
Pos	378.2385	7.30	M	Yes	CLR	-
Pos	409.7493	7.42	M	Yes	CLR	-
Pos	421.7779	6.60	M	Yes	Visual inspection	-
Pos	434.6859	0.74	U	Yes	Visual inspection	-
Pos	438.2934	6.53	M	Yes	CLR	-
Pos	500.2714	6.16	M	Yes	CLR	-
Pos	501.2751	6.16	M	Yes	CLR	-
Pos	515.3069	6.41	M	Yes	CLR	-
Pos	515.8086	6.41	M	Yes	CLR	-
Pos	522.7918	6.41	M	Yes	CLR	-
Pos	523.7946	6.41	M	Yes	CLR	-
Pos	547.2927	6.32	M	Yes	CLR	-
Pos	551.3234	6.59	M	Yes	CLR	-
Pos	555.4068	6.41	M	Yes	CLR	-
Pos	564.2996	6.19	M	Yes	CLR	-
Pos	565.3031	6.19	M	Yes	CLR	-
Pos	675.5366	7.24	M	Yes	CLR	-
Pos	676.5393	7.24	M	No	-	-
Pos	677.5417	7.24	M	Yes	Adduct/isotope	M+1 of 676
Pos	677.5490	7.36	M	No	-	-

Pos	689.5513	7.37	M	No	-	-
Pos	690.5542	7.37	M	Yes	Adduct/isotope	M+1 of 689
Pos	691.5570	7.37	M	Yes	Adduct/isotope	M+2 of 689
Pos	692.5582	7.38	M	Yes	Adduct/isotope	M+3 of 689
Pos	697.5171	7.24	MU	Yes	Adduct/isotope	M+Na of 675, which is parent compound of 676 (M+1)
Pos	698.5202	7.24	MU	Yes	Adduct/isotope	M+1 of 697
Pos	701.5518	7.29	M	No	-	-
Pos	702.5544	7.29	M	Yes	CLR	-
Pos	703.5678	7.51	MU	Yes	CLR	-
Pos	704.5703	7.51	MU	Yes	CLR	-
Pos	705.5726	7.51	MU	No	-	-
Pos	711.5321	7.37	M	Yes	Adduct/isotope	M+Na of 689
Pos	712.5355	7.37	M	Yes	CLR	-
Pos	715.5655	7.43	M	No	-	-
Pos	716.5688	7.43	MU	Yes	Adduct/isotope	M+1 of 715
Pos	723.5317	7.29	M	Yes	Adduct/isotope	M+Na of 701
Pos	724.5350	7.29	M	Yes	Adduct/isotope	M+1 of 723
Pos	725.5476	7.51	M	Yes	CLR	-
Pos	726.5508	7.51	M	Yes	CLR	-
Pos	727.5653	7.35	MU	No	-	-
Pos	728.5685	7.35	M	Yes	Adduct/isotope	M+1 of 727
Pos	729.5813	7.57	MU	No	-	-
Pos	730.5291	7.36	MU	No	-	-
Pos	730.5843	7.57	MU	Yes	Adduct/isotope	M+1 of 729
Pos	731.5320	7.36	MU	Yes	Adduct/isotope	M+1 of 730
Pos	731.5968	7.83	M	No	-	-
Pos	732.5447	7.57	MU	No	-	-
Pos	732.5998	7.83	M	Yes	CLR	-
Pos	733.5478	7.57	MU	Yes	Adduct/isotope	M+1 of 732
Pos	733.6028	7.83	M	Yes	CLR	-
Pos	744.5441	7.50	MU	No	-	-
Pos	745.5479	7.50	MU	Yes	Adduct/isotope	M+1 of 745
Pos	746.5595	7.73	M	No	-	-
Pos	747.5631	7.73	M	Yes	CLR	-
Pos	752.5110	7.35	MU	Yes	Adduct/isotope	M+Na of 730
Pos	756.5441	7.45	MU	No	-	-
Pos	757.5473	7.45	MU	Yes	Adduct/isotope	M+1 of 756
Pos	757.6113	7.88	MU	No	-	-
Pos	758.5509	7.44	U	Yes	CLR	-
Pos	758.5611	7.66	MU	Yes	CLR	-
Pos	759.5638	7.66	MU	No	-	-
Pos	760.5662	7.65	U	Yes	CLR	-
Pos	760.5764	7.89	MU	Yes	CLR	-
Pos	761.5687	7.65	U	Yes	Adduct/isotope	M+2 of 759
Pos	761.5791	7.89	MU	No	-	-
Pos	762.5816	7.89	M	Yes	CLR	-
Pos	763.5842	7.89	MU	Yes	Adduct/isotope	M+2 of 761
Pos	768.5431	7.46	MU	No	-	-
Pos	769.5480	7.46	U	Yes	Adduct/isotope	M+1 of 768
Pos	770.5586	7.57	MU	Yes	Visual inspection	-
Pos	770.9559	6.41	M	Yes	CLR	-
Pos	772.0667	7.89	M	Yes	CLR	-
Pos	772.5748	7.80	M	No	-	-
Pos	773.5779	7.80	M	Yes	Adduct/isotope	M+1 of 772
Pos	783.5621	7.59	M	Yes	CLR	-
Pos	784.5755	7.73	M	Yes	CLR	-
Pos	785.5783	7.73	MU	No	-	-
Pos	786.5914	7.98	MU	Yes	CLR	-

Pos	787.5941	7.98	M	No	-	-
Pos	788.5965	7.98	U	Yes	Adduct/isotope	M+1 of 787
Pos	793.5475	7.41	M	Yes	CLR	-
Pos	821.6385	8.47	M	Yes	Visual inspection	-
Pos	829.5612	4.86	M	Yes	CLR	-
Pos	829.9371	4.86	M	Yes	CLR	-
Pos	832.5727	7.59	U	Yes	Visual inspection	-
Pos	947.7818	4.86	M	Yes	CLR	-
Pos	947.9254	4.87	M	Yes	CLR	-
Pos	948.4975	4.86	M	Yes	CLR	-
Pos	948.6405	4.86	M	Yes	CLR	-
Pos	987.6250	6.26	M	Yes	CLR	-
Pos	991.6579	6.41	M	Yes	CLR	-
Pos	992.6612	6.41	M	Yes	CLR	-
Pos	993.6633	6.41	M	Yes	CLR	-
Pos	994.6653	6.41	M	Yes	CLR	-
Pos	995.6677	6.41	M	Yes	CLR	-
Pos	1001.6405	6.31	M	Yes	CLR	-
Pos	1005.6723	6.47	M	Yes	CLR	-
Pos	1006.6760	6.48	M	Yes	CLR	-
Pos	1015.6547	6.39	M	Yes	CLR	-
Pos	1016.6583	6.38	M	Yes	CLR	-
Pos	1017.6705	6.44	M	Yes	CLR	-
Pos	1019.6766	6.44	M	Yes	CLR	-
Pos	1020.6794	6.43	M	Yes	CLR	-
Pos	1021.6077	6.31	M	Yes	CLR	-
Pos	1022.6119	6.31	M	Yes	CLR	-
Pos	1038.6415	6.35	M	Yes	CLR	-
Pos	1041.6676	6.40	M	Yes	CLR	-
Pos	1045.6066	6.30	M	Yes	CLR	-
Pos	1046.6109	6.30	M	Yes	CLR	-
Pos	1046.7033	6.58	M	Yes	CLR	-
Pos	1047.6183	6.32	M	Yes	CLR	-
Pos	1061.6354	6.31	M	Yes	CLR	-
Pos	1062.6398	6.31	M	Yes	CLR	-
Pos	1063.6523	6.31	M	Yes	CLR	-
Pos	1064.6558	6.31	M	Yes	CLR	-
Pos	1065.6633	6.33	M	Yes	CLR	-
Pos	1066.3317	7.51	M	Yes	CLR	-
Pos	1069.6058	6.29	M	Yes	CLR	-
Pos	1085.6351	6.30	M	Yes	CLR	-
Pos	1087.6513	6.30	M	Yes	CLR	-
Pos	1088.6550	6.30	M	Yes	CLR	-
Pos	1089.6634	6.32	M	Yes	CLR	-
Pos	1090.6677	6.32	M	Yes	CLR	-
Pos	1105.5755	4.87	M	Yes	CLR	-

**Feature**			**Non-aggressive prostate cancer (587 pairs)**			
**Polarity**	***m/z***	**RT (min)**	**Statistical procedure**	**Filtered, yes/no**	**Filtering step**	**Adduct/isotope detail**
Neg	311.2002	6.16	M	Yes	CLR	-
Neg	356.1849	6.23	M	Yes	Visual inspection	-
Neg	368.2656	6.53	MU	Yes	CLR	-
Neg	409.2343	6.40	M	Yes	CLR	-
Neg	410.2372	6.40	M	Yes	CLR	-
Neg	412.2472	5.95	M	Yes	CLR	-
Neg	508.3365	6.58	M	Yes	CLR	-
Neg	540.3273	6.40	M	Yes	CLR	-
Neg	541.3305	6.40	M	Yes	CLR	-
Neg	568.3581	6.58	M	Yes	CLR	-

Neg	569.3614	6.58	M	Yes	CLR	-
Neg	748.5213	7.45	M	Yes	CLR	-
Pos	205.0836	5.96	M	Yes	CLR	-
Pos	226.9491	0.64	U	Yes	Visual inspection	-
Pos	229.2139	6.50	M	Yes	Visual inspection	-
Pos	239.2345	6.69	M	Yes	CLR	-
Pos	257.1486	6.11	MU	No	-	-
Pos	257.2450	6.69	MU	No	-	-
Pos	258.2480	6.69	MU	Yes	Adduct/isotope	M+1 of 257
Pos	301.1381	5.95	M	Yes	CLR	-
Pos	302.1413	5.95	M	Yes	CLR	-
Pos	315.1900	6.09	M	Yes	CLR	-
Pos	357.2457	7.24	M	Yes	CLR	-
Pos	370.2526	7.30	M	Yes	CLR	-
Pos	370.7369	0.74	M	Yes	CLR	-
Pos	371.2604	7.51	M	Yes	CLR	-
Pos	378.2385	7.30	M	Yes	CLR	-
Pos	379.2396	7.30	M	Yes	Visual inspection	-
Pos	409.7493	7.42	M	Yes	CLR	-
Pos	421.7779	6.60	M	Yes	CLR	-
Pos	434.6859	0.74	M	Yes	Visual inspection	-
Pos	438.2934	6.53	M	Yes	CLR	-
Pos	512.3290	6.23	M	Yes	CLR	-
Pos	515.3069	6.41	M	Yes	CLR	-
Pos	515.8086	6.41	M	Yes	CLR	-
Pos	522.7918	6.41	M	Yes	CLR	-
Pos	523.7946	6.41	M	Yes	CLR	-
Pos	539.4801	6.27	M	Yes	CLR	-
Pos	547.2927	6.32	M	Yes	CLR	-
Pos	551.3234	6.59	M	Yes	CLR	-
Pos	555.4068	6.41	M	Yes	CLR	-
Pos	564.2996	6.19	M	Yes	CLR	-
Pos	565.3031	6.19	M	Yes	CLR	-
Pos	570.3470	6.35	M	Yes	CLR	-
Pos	675.5366	7.24	M	Yes	CLR	-
Pos	676.5393	7.24	M	No	-	-
Pos	677.5417	7.24	M	Yes	Adduct/isotope	M+1 of 676
Pos	677.5490	7.36	M	No	-	-
Pos	689.5513	7.37	M	Yes	CLR	-
Pos	690.5542	7.37	M	No	-	-
Pos	691.5570	7.37	M	Yes	Adduct/isotope	M+1 of 690
Pos	692.5582	7.38	M	Yes	CLR	-
Pos	697.5171	7.24	MU	Yes	CLR	-
Pos	698.5202	7.24	MU	Yes	Adduct/isotope	M+Na of 676
Pos	701.5518	7.29	M	Yes	CLR	-
Pos	702.5544	7.29	M	Yes	CLR	-
Pos	703.5678	7.51	MU	Yes	CLR	-
Pos	704.5703	7.51	MU	Yes	CLR	-
Pos	705.5726	7.51	MU	Yes	Covar	-
Pos	706.5715	7.51	M	Yes	CLR	-
Pos	711.5321	7.37	M	Yes	CLR	-
Pos	712.5355	7.37	M	Yes	CLR	-
Pos	715.5655	7.43	M	Yes	Visual inspection	-
Pos	716.5688	7.43	M	Yes	Adduct/isotope	M+1 of 715
Pos	723.5317	7.29	M	Yes	CLR	-
Pos	724.5350	7.29	M	Yes	CLR	-
Pos	725.5476	7.51	M	Yes	CLR	-
Pos	726.5508	7.51	M	Yes	CLR	-
Pos	727.5653	7.35	M	No	-	-

Pos	728.5685	7.35	M	Yes	Adduct/isotope	M+1 of 727
Pos	729.5813	7.57	MU	No	-	-
Pos	730.5291	7.36	M	No	-	-
Pos	730.5843	7.57	MU	Yes	Adduct/isotope	M+1 of 729
Pos	731.5320	7.36	M	Yes	Adduct/isotope	M+1 of 730
Pos	731.5968	7.83	M	Yes	CLR	-
Pos	732.5998	7.83	M	Yes	CLR	-
Pos	733.6028	7.83	M	Yes	CLR	-
Pos	734.5600	7.84	M	No	-	-
Pos	744.5441	7.50	M	No	-	-
Pos	745.5479	7.50	MU	Yes	Adduct/isotope	M+1 of 744
Pos	747.5631	7.73	M	Yes	CLR	-
Pos	752.5110	7.35	M	Yes	Adduct/isotope	M+Na of 730
Pos	756.5441	7.45	M	No	-	-
Pos	757.5473	7.45	MU	Yes	Adduct/isotope	M+1 of 756
Pos	757.6113	7.88	M	Yes	CLR	-
Pos	758.5611	7.66	M	Yes	CLR	-
Pos	759.5638	7.66	MU	No	-	-
Pos	760.5764	7.89	M	Yes	CLR	-
Pos	761.5791	7.89	MU	No	-	-
Pos	762.5816	7.89	M	Yes	CLR	-
Pos	763.5842	7.89	M	Yes	CLR	-
Pos	768.5431	7.46	M	No	-	-
Pos	770.5586	7.57	MU	Yes	Visual inspection	-
Pos	770.9559	6.41	M	Yes	CLR	-
Pos	772.0667	7.89	M	Yes	CLR	-
Pos	772.5748	7.80	M	No	-	-
Pos	773.5779	7.80	M	Yes	Adduct/isotope	M+1 of 772
Pos	782.5591	7.60	M	No	-	-
Pos	783.5621	7.59	M	Yes	CLR	-
Pos	784.5755	7.73	M	Yes	CLR	-
Pos	785.5783	7.73	M	No	-	-
Pos	786.5914	7.98	M	No	-	-
Pos	787.5941	7.98	M	Yes	Adduct/isotope	M+1 of 786
Pos	821.6385	8.47	M	Yes	CLR	-
Pos	828.5398	7.54	M	Yes	CLR	-
Pos	829.5431	7.54	M	Yes	CLR	-
Pos	829.5612	4.86	M	Yes	CLR	-
Pos	829.6862	4.87	M	Yes	CLR	-
Pos	829.8116	4.86	M	Yes	CLR	-
Pos	829.9371	4.86	M	Yes	CLR	-
Pos	833.6384	8.32	M	Yes	CLR	-
Pos	947.7818	4.86	M	Yes	CLR	-
Pos	947.9254	4.87	M	Yes	CLR	-
Pos	948.0679	4.87	M	Yes	CLR	-
Pos	948.3545	4.86	M	Yes	CLR	-
Pos	948.4975	4.86	M	Yes	CLR	-
Pos	948.6405	4.86	M	Yes	CLR	-
Pos	949.6108	6.41	M	Yes	CLR	-
Pos	975.6263	6.44	M	Yes	CLR	-
Pos	976.6310	6.44	M	Yes	CLR	-
Pos	987.6250	6.26	M	Yes	CLR	-
Pos	991.6579	6.41	M	Yes	CLR	-
Pos	992.6612	6.41	M	Yes	CLR	-
Pos	993.6633	6.41	M	Yes	CLR	-
Pos	994.6653	6.41	M	Yes	CLR	-
Pos	995.6677	6.41	M	Yes	CLR	-
Pos	1001.6405	6.31	M	Yes	CLR	-
Pos	1003.6516	6.40	M	Yes	CLR	-

Pos	1005.6723	6.47	M	Yes	CLR	-
Pos	1006.6760	6.48	M	Yes	CLR	-
Pos	1013.6377	6.40	M	Yes	CLR	-
Pos	1014.6415	6.40	M	Yes	CLR	-
Pos	1015.6547	6.39	M	Yes	CLR	-
Pos	1016.6583	6.38	M	Yes	CLR	-
Pos	1017.6705	6.44	M	Yes	CLR	-
Pos	1019.6766	6.44	M	Yes	CLR	-
Pos	1020.6794	6.43	M	Yes	CLR	-
Pos	1021.6077	6.31	M	Yes	CLR	-
Pos	1022.6119	6.31	M	Yes	CLR	-
Pos	1031.6858	6.47	M	Yes	CLR	-
Pos	1032.6885	6.47	M	Yes	CLR	-
Pos	1037.6360	6.35	M	Yes	CLR	-
Pos	1038.6415	6.35	M	Yes	CLR	-
Pos	1040.6575	6.32	M	Yes	CLR	-
Pos	1041.6676	6.40	M	Yes	CLR	-
Pos	1042.6721	6.40	M	Yes	CLR	-
Pos	1044.6879	6.45	M	Yes	CLR	-
Pos	1045.6066	6.30	M	Yes	CLR	-
Pos	1045.6996	6.58	M	Yes	CLR	-
Pos	1046.6109	6.30	M	Yes	CLR	-
Pos	1046.7033	6.58	M	Yes	CLR	-
Pos	1047.6183	6.32	M	Yes	CLR	-
Pos	1047.7160	6.59	M	Yes	CLR	-
Pos	1049.7224	6.59	M	Yes	CLR	-
Pos	1050.7254	6.59	M	Yes	CLR	-
Pos	1061.6354	6.31	M	Yes	CLR	-
Pos	1062.6398	6.31	M	Yes	CLR	-
Pos	1063.6523	6.31	M	Yes	CLR	-
Pos	1064.6558	6.31	M	Yes	CLR	-
Pos	1065.6633	6.33	M	Yes	CLR	-
Pos	1066.3317	7.51	M	Yes	CLR	-
Pos	1066.6676	6.34	M	Yes	CLR	-
Pos	1068.6868	6.43	M	Yes	CLR	-
Pos	1069.6058	6.29	M	Yes	CLR	-
Pos	1085.6351	6.30	M	Yes	CLR	-
Pos	1087.6513	6.30	M	Yes	CLR	-
Pos	1088.6550	6.30	M	Yes	CLR	-
Pos	1089.6633	6.32	M	Yes	CLR	-
Pos	1090.6677	6.32	M	Yes	CLR	-
Pos	1105.5755	4.87	M	Yes	CLR	-
Pos	1105.7424	4.86	M	Yes	CLR	-
Pos	1106.0764	4.86	M	Yes	CLR	-

**Feature**			**Aggressive prostate cancer (165 pairs)**			
**Polarity**	***m/z***	**RT (min)**	**Statistical procedure**	**Filtered, yes/no**	**Filtering step**	**Adduct/isotope detail**
Pos	188.0685	2.24	U	Yes	CLR	-
Pos	189.0715	2.24	U	Yes	CLR	-
Pos	205.0950	2.24	U	Yes	Visual inspection	-
Pos	206.0979	2.24	U	Yes	Adduct/isotope	M+1 of 205
Pos	227.0764	2.24	U	Yes	CLR	-
Pos	409.1826	2.24	U	Yes	CLR	-

**Feature**			**Younger subgroup of 40- and 50-year-olds at baseline (326 pairs)**			
**Polarity**	***m/z***	**RT (min)**	**Statistical procedure**	**Filtered, yes/no**	**Filtering step**	**Adduct/isotope detail**
Neg	279.2319	6.32	M	Yes	CLR	-
Neg	620.5933	8.87	M	Yes	CLR	-
Neg	645.5961	8.50	M	Yes	CLR	-

Neg	750.5378	7.68	M	Yes	CLR	-
Neg	857.6637	8.67	MU	Yes	CLR	-
Neg	887.6739	8.69	MU	Yes	CLR	-
Pos	135.0010	1.05	M	Yes	CLR	-
Pos	158.9620	0.64	M	No	-	-
Pos	162.0480	0.93	M	Yes	CLR	-
Pos	176.0635	1.54	M	Yes	CLR	-
Pos	201.1828	6.25	M	Yes	CLR	-
Pos	203.0507	0.73	M	Yes	CLR	-
Pos	206.8915	0.69	M	No	-	-
Pos	208.8886	0.69	M	Yes	Adduct/isotope	M+2 of 206
Pos	211.1069	5.92	M	Yes	CLR	-
Pos	216.9203	0.68	MU	No	-	-
Pos	218.9173	0.68	M	Yes	Adduct/isotope	M+2 of 216
Pos	226.0424	1.16	MU	Yes	CLR	-
Pos	226.9491	0.64	MU	No	-	-
Pos	227.0764	2.25	MU	Yes	CLR	-
Pos	229.2139	6.50	M	Yes	CLR	-
Pos	257.1486	6.11	MU	Yes	CLR	-
Pos	257.2450	6.69	M	Yes	CLR	-
Pos	258.2480	6.69	M	Yes	CLR	-
Pos	263.0820	0.72	M	Yes	Visual inspection	-
Pos	265.2499	6.73	M	Yes	CLR	-
Pos	267.1692	5.92	M	Yes	CLR	-
Pos	270.9552	0.96	M	Yes	CLR	-
Pos	272.9523	0.96	M	Yes	CLR	-
Pos	283.2605	6.73	M	Yes	CLR	-
Pos	284.2637	6.73	M	Yes	CLR	-
Pos	285.2759	6.91	M	Yes	CLR	-
Pos	289.1510	5.92	M	Yes	CLR	-
Pos	293.2421	6.97	M	Yes	CLR	-
Pos	303.2265	6.61	MU	No	-	-
Pos	305.2423	6.73	MU	Yes	CLR	-
Pos	306.2454	6.73	M	Yes	CLR	-
Pos	315.1900	6.09	M	Yes	CLR	-
Pos	326.2420	5.92	M	Yes	CLR	-
Pos	337.1640	6.61	M	Yes	CLR	-
Pos	342.8651	0.68	M	Yes	CLR	-
Pos	352.8937	0.67	M	Yes	CLR	-
Pos	369.3478	7.70	M	Yes	CLR	-
Pos	370.7369	0.74	M	Yes	CLR	-
Pos	372.7339	0.74	M	Yes	CLR	-
Pos	374.7309	0.74	M	Yes	CLR	-
Pos	379.2396	7.30	M	Yes	CLR	-
Pos	383.1125	0.73	M	Yes	CLR	-
Pos	384.1153	0.73	MU	Yes	CLR	-
Pos	390.7943	0.68	M	Yes	CLR	-
Pos	392.7913	0.68	MU	Yes	CLR	-
Pos	400.8230	0.68	M	Yes	CLR	-
Pos	402.8202	0.68	MU	No	-	-
Pos	410.8517	0.67	M	Yes	CLR	-
Pos	412.8489	0.67	M	No	-	-
Pos	420.8804	0.67	M	No	-	-
Pos	421.7779	6.60	M	Yes	CLR	-
Pos	430.6919	0.74	M	Yes	CLR	-
Pos	432.6889	0.74	M	Yes	CLR	-
Pos	434.6859	0.74	M	Yes	CLR	-
Pos	460.7781	0.68	MU	Yes	CLR	-
Pos	466.3244	6.71	M	Yes	CLR	-

Pos	480.3377	6.52	M	Yes	CLR	-
Pos	488.6497	0.74	M	Yes	CLR	-
Pos	490.6468	0.74	M	Yes	CLR	-
Pos	506.3535	6.58	M	Yes	CLR	-
Pos	524.3680	6.59	M	Yes	CLR	-
Pos	525.3700	6.59	M	Yes	CLR	-
Pos	526.3719	6.59	M	Yes	CLR	-
Pos	539.4969	6.72	M	Yes	CLR	-
Pos	555.4068	6.41	M	Yes	Visual inspection	-
Pos	565.5124	6.73	M	Yes	CLR	-
Pos	570.3470	6.35	M	Yes	CLR	-
Pos	571.3512	6.35	M	Yes	CLR	-
Pos	621.4318	6.74	M	Yes	Visual inspection	-
Pos	661.5199	7.12	M	Yes	CLR	-
Pos	673.5199	7.05	M	Yes	CLR	-
Pos	674.5229	7.05	M	Yes	CLR	-
Pos	675.5366	7.24	M	Yes	CLR	-
Pos	676.5393	7.24	M	Yes	CLR	-
Pos	677.5417	7.24	M	Yes	CLR	-
Pos	677.5490	7.36	M	Yes	CLR	-
Pos	687.5351	7.17	M	Yes	CLR	-
Pos	689.5513	7.37	M	Yes	CLR	-
Pos	690.5542	7.37	M	Yes	CLR	-
Pos	691.5570	7.37	M	Yes	CLR	-
Pos	697.5171	7.24	M	Yes	CLR	-
Pos	698.5202	7.24	M	Yes	CLR	-
Pos	699.5257	7.24	M	Yes	CLR	-
Pos	701.5518	7.29	M	Yes	CLR	-
Pos	702.5544	7.29	M	Yes	CLR	-
Pos	703.5678	7.51	M	Yes	CLR	-
Pos	704.5703	7.51	M	Yes	CLR	-
Pos	705.5726	7.51	M	Yes	CLR	-
Pos	715.5655	7.43	M	Yes	CLR	-
Pos	716.5688	7.43	M	Yes	CLR	-
Pos	727.5653	7.35	M	No	-	-
Pos	728.5685	7.35	M	Yes	CLR	-
Pos	729.5813	7.57	M	Yes	CLR	-
Pos	730.5291	7.36	M	Yes	CLR	-
Pos	731.5320	7.36	M	Yes	CLR	-
Pos	744.5441	7.50	M	Yes	CLR	-
Pos	745.5479	7.50	M	Yes	CLR	-
Pos	752.5110	7.35	M	Yes	CLR	-
Pos	756.5441	7.45	M	Yes	CLR	-
Pos	757.5473	7.45	M	Yes	CLR	-
Pos	758.5611	7.66	M	Yes	CLR	-
Pos	759.5638	7.66	M	Yes	CLR	-
Pos	770.5586	7.57	M	Yes	CLR	-
Pos	786.5914	7.98	M	Yes	CLR	-
Pos	787.5941	7.98	M	Yes	CLR	-
Pos	792.5421	7.41	M	Yes	CLR	-
Pos	829.5431	7.54	M	Yes	CLR	-
Pos	832.5727	7.59	M	Yes	CLR	-
Pos	992.6612	6.41	M	Yes	CLR	-
Pos	993.6633	6.41	M	Yes	CLR	-
Pos	994.6653	6.41	M	Yes	CLR	-
Pos	995.6677	6.41	M	Yes	CLR	-
Pos	1017.6705	6.44	M	Yes	CLR	-
Pos	1019.6766	6.44	M	Yes	CLR	-
Pos	1020.6794	6.43	M	Yes	CLR	-

Pos	1021.6077	6.31	M	Yes	CLR	-
Pos	1022.6119	6.31	M	Yes	CLR	-
Pos	1045.6066	6.30	M	Yes	CLR	-
Pos	1046.6109	6.30	M	Yes	CLR	-
Pos	1047.6183	6.32	M	Yes	CLR	-
Pos	1050.7254	6.59	M	Yes	CLR	-
Pos	1061.6354	6.31	M	Yes	CLR	-
Pos	1062.6398	6.31	M	Yes	CLR	-
Pos	1063.6523	6.31	M	Yes	CLR	-
Pos	1064.6558	6.31	M	Yes	CLR	-
Pos	1065.6633	6.33	M	Yes	CLR	-
Pos	1069.6058	6.29	M	Yes	CLR	-
Pos	1085.6351	6.30	M	Yes	CLR	-
Pos	1087.6513	6.30	M	Yes	CLR	-
Pos	1088.6550	6.30	M	Yes	CLR	-
Pos	1089.6633	6.32	M	Yes	CLR	-
Pos	1090.6677	6.32	M	Yes	CLR	-

**Feature**			**Older subgroup of 60-year-olds at baseline (426 pairs)**			
**Polarity**	***m/z***	**RT (min)**	**Statistical procedure**	**Filtered, yes/no**	**Filtering step**	**Adduct/isotope detail**
Neg	179.0550	0.72	M	No	-	-
Neg	290.2108	5.45	U	Yes	CLR	-
Neg	311.2002	6.16	MU	No	-	-
Neg	356.1849	6.23	MU	No	-	-
Neg	367.2623	6.54	M	No	-	-
Neg	368.2656	6.53	MU	Yes	Adduct/isotope	M+1 of 367
Neg	409.2343	6.40	M	Yes	CLR	-
Neg	410.2372	6.40	M	Yes	CLR	-
Neg	412.2472	5.95	MU	No	-	-
Neg	480.3061	6.40	M	Yes	CLR	-
Neg	508.3365	6.58	M	Yes	CLR	-
Neg	540.3273	6.40	M	Yes	CLR	-
Neg	541.3305	6.40	M	Yes	CLR	-
Neg	748.5213	7.45	M	Yes	CLR	-
Neg	778.5876	6.40	M	No	-	-
Pos	187.0554	0.80	U	No	-	-
Pos	191.0152	0.96	M	No	-	-
Pos	205.0836	5.96	M	No	-	-
Pos	239.2345	6.69	M	Yes	Adduct/isotope	M-H_2O of 257
Pos	257.1486	6.11	MU	No	-	-
Pos	257.2450	6.69	MU	No	-	-
Pos	258.2480	6.69	M	Yes	Adduct/isotope	M+1 of 257
Pos	301.1381	5.95	M	No	-	-
Pos	302.1413	5.95	M	Yes	Adduct/isotope	M+1 of 301
Pos	357.2457	7.24	M	No	-	-
Pos	370.2526	7.30	M	No	-	-
Pos	371.2604	7.51	M	Yes	CLR	-
Pos	378.2385	7.30	M	No	-	-
Pos	380.2520	6.23	M	No	-	-
Pos	409.7493	7.42	M	No	-	-
Pos	421.7779	6.60	M	No	-	-
Pos	437.3197	6.70	M	No	-	-
Pos	438.2934	6.53	M	No	-	-
Pos	496.3380	6.41	M	Yes	CLR	-
Pos	515.3069	6.41	M	No	-	-
Pos	515.8086	6.41	M	No	-	-
Pos	522.7918	6.41	MU	No	-	-
Pos	523.2933	6.41	M	No	-	-

Pos	523.7946	6.41	M	Yes	Adduct/isotope	M+1 of 522
Pos	539.3063	6.32	M	No	-	-
Pos	547.2927	6.32	M	No	-	-
Pos	551.3234	6.59	M	No	-	-
Pos	555.4068	6.41	M	Yes	CLR	-
Pos	603.5275	8.52	M	No	-	-
Pos	638.5643	8.55	MU	No	-	-
Pos	643.5195	8.55	MU	No	-	-
Pos	644.5229	8.55	M	Yes	Adduct/isotope	M+1 of 643
Pos	675.5366	7.24	M	Yes	CLR	-
Pos	676.5393	7.24	M	No	-	-
Pos	677.5417	7.24	M	Yes	CLR	-
Pos	677.5490	7.36	M	No	-	-
Pos	689.5513	7.37	M	No	-	-
Pos	690.5542	7.37	M	Yes	Adduct/isotope	M+1 of 689
Pos	691.5570	7.37	M	Yes	Adduct/isotope	M+2 of 689
Pos	692.5582	7.38	M	Yes	Adduct/isotope	M+3 of 689
Pos	697.5171	7.24	M	Yes	Adduct/isotope	M+Na of 675, which is parent compound of 676 (M+1)
Pos	698.5202	7.24	MU	Yes	Adduct/isotope	M+1 of 697
Pos	701.5518	7.29	M	No	-	-
Pos	702.5544	7.29	M	Yes	CLR	-
Pos	703.5678	7.51	M	Yes	CLR	-
Pos	704.5703	7.51	M	No	-	-
Pos	705.5726	7.51	M	Yes	Adduct/isotope	M+1 of 704
Pos	707.3203	6.80	M	Yes	CLR	-
Pos	711.5321	7.37	M	Yes	Adduct/isotope	M+Na of 689
Pos	712.5355	7.37	M	Yes	Adduct/isotope	M+1 of 711
Pos	715.5655	7.43	M	No	-	-
Pos	716.5688	7.43	M	Yes	Adduct/isotope	M+1 of 715
Pos	723.5317	7.29	M	Yes	Adduct/isotope	M+Na of 701
Pos	724.5350	7.29	M	Yes	Adduct/isotope	M+1 of 723
Pos	725.5476	7.51	M	Yes	Covar	-
Pos	726.5508	7.51	M	Yes	Adduct/isotope	M+Na of 704
Pos	727.5653	7.35	M	No	-	-
Pos	728.5685	7.35	M	Yes	Adduct/isotope	M+1 of 727
Pos	729.5813	7.57	MU	No	-	-
Pos	730.5291	7.36	M	No	-	-
Pos	730.5843	7.57	MU	Yes	Adduct/isotope	M+1 of 729
Pos	731.5320	7.36	M	Yes	Adduct/isotope	M+1 of 730
Pos	731.5968	7.83	MU	No	-	-
Pos	732.5447	7.57	M	No	-	-
Pos	732.5998	7.83	MU	Yes	Adduct/isotope	M+1 of 731
Pos	733.5478	7.57	M	Yes	Adduct/isotope	M+1 of 732
Pos	733.6028	7.83	MU	Yes	Adduct/isotope	M+1 of 732
Pos	744.5441	7.50	M	No	-	-
Pos	745.5479	7.50	M	Yes	Adduct/isotope	M+1 of 744
Pos	746.5595	7.73	M	No	-	-
Pos	747.5631	7.73	M	Yes	Adduct/isotope	M+1 of 746
Pos	752.5110	7.35	M	Yes	Adduct/isotope	M+Na of 730
Pos	756.5441	7.45	M	No	-	-
Pos	757.5473	7.45	M	Yes	Adduct/isotope	M+1 of 756
Pos	757.6113	7.88	MU	No	-	-
Pos	758.5611	7.66	MU	Yes	CLR	-
Pos	759.5638	7.66	MU	No	-	-
Pos	759.6273	8.20	U	No	-	-
Pos	760.5764	7.89	MU	No	-	-
Pos	761.5687	7.65	U	Yes	Adduct/isotope	M+2 of 759
Pos	761.5791	7.89	MU	Yes	Adduct/isotope	M+1 of 760

Pos	762.5816	7.89	M	Yes	Adduct/isotope	M+2 of 760
Pos	763.5842	7.89	MU	Yes	Adduct/isotope	M+3 of 760
Pos	768.5431	7.46	M	No	-	-
Pos	770.5586	7.57	M	No	-	-
Pos	770.9559	6.41	MU	Yes	CLR	-
Pos	772.0667	7.89	M	No	-	-
Pos	772.5748	7.80	M	No	-	-
Pos	773.5779	7.80	M	Yes	Adduct/isotope	M+1 of 772
Pos	774.6455	8.41	M	No	-	-
Pos	782.5591	7.60	M	No	-	-
Pos	783.5621	7.59	M	Yes	CLR	-
Pos	784.5755	7.73	MU	No	-	-
Pos	785.5783	7.73	MU	Yes	Adduct/isotope	M+1 of 784
Pos	786.5809	7.72	U	Yes	Adduct/isotope	M+2 of 784
Pos	786.5914	7.98	M	Yes	Covar	-
Pos	787.5941	7.98	M	No	-	-
Pos	788.5965	7.98	M	Yes	Adduct/isotope	M+1 of 787
Pos	799.6575	8.47	M	No	-	-
Pos	800.6054	8.16	M	No	-	-
Pos	811.5925	7.92	M	No	-	-
Pos	821.6385	8.47	M	Yes	Adduct/isotope	M+Na of 799
Pos	829.5612	4.86	M	Yes	CLR	-
Pos	829.6862	4.87	M	Yes	CLR	-
Pos	829.8116	4.86	M	Yes	CLR	-
Pos	829.9371	4.86	M	Yes	CLR	-
Pos	833.6384	8.32	M	No	-	-
Pos	947.7818	4.86	M	Yes	CLR	-
Pos	947.9254	4.87	M	Yes	CLR	-
Pos	948.0679	4.87	M	Yes	CLR	-
Pos	948.3545	4.86	M	Yes	CLR	-
Pos	948.4975	4.86	M	Yes	CLR	-
Pos	948.6405	4.86	M	Yes	CLR	-
Pos	987.6250	6.26	M	Yes	CLR	-
Pos	991.6579	6.41	M	Yes	CLR	-
Pos	992.6612	6.41	M	Yes	CLR	-
Pos	993.6633	6.41	M	Yes	CLR	-
Pos	994.6653	6.41	M	Yes	CLR	-
Pos	995.6677	6.41	M	Yes	CLR	-
Pos	1005.6723	6.47	M	Yes	CLR	-
Pos	1006.6760	6.48	M	Yes	CLR	-
Pos	1015.6547	6.39	M	Yes	CLR	-
Pos	1016.6583	6.38	M	Yes	CLR	-
Pos	1017.6705	6.44	M	Yes	CLR	-
Pos	1019.6766	6.44	M	Yes	CLR	-
Pos	1045.6066	6.30	M	Yes	CLR	-
Pos	1046.6109	6.30	M	Yes	CLR	-
Pos	1063.6523	6.31	M	Yes	CLR	-
Pos	1064.6558	6.31	M	Yes	CLR	-
Pos	1066.3317	7.51	M	Yes	CLR	-
Pos	1069.6058	6.29	M	Yes	CLR	-
Pos	1087.6513	6.30	M	Yes	CLR	-
Pos	1088.6550	6.30	M	Yes	CLR	-
Pos	1089.6633	6.32	M	Yes	CLR	-
Pos	1090.6677	6.32	M	Yes	CLR	-
Pos	1105.5755	4.87	M	Yes	CLR	-

RT, retention time; M, multivariate statistics, orthogonal projections to latent structures-effect projections (OPLS-EP) model with cross-validated analysis of variance (CV-ANOVA), $p<0.05$, variable importance on projection (VIP) ≥1.5, and VIP 95%CI >0; U, univariate statistics, two-sided Wilcoxon signed-rank test with Bonferroni correction, $p<0.05$. CLR, conditional logistic regression, $p>0.05$. Covar, features excluded if the conditional logistic regression (CLR) p-value rose above 0.05 or if the odds ratio changed by more than 10% after inclusion of the covariates exact age, body mass index, alcohol consumption (<10, 10-19, 20-39, ≥40 g/day), and smoking status (no, past, current, unknown) in the CLR model. Isotope and adduct filtering was done by

assessing whether features with the same retention time differed in *m/z* corresponding to ^{13}C isotope differences ($\Delta = n \times 1.0033$), Na^+-adducts ($\Delta = 21.9819$), or in-source H_2O ($\Delta = 18.0153$) or NH_3 ($\Delta = 17.0266$) loss, retaining (by decreasing priority) the $[M+H]^+$ adduct, the lowest *m/z* isotopolog, or an in-source fragment rather than Na^+-adduct.

Supplementary Table 2. *Association between discriminating features identified by orthogonal projection of latent structures-effect projections (OPLS-EP) and prostate cancer risk*

Feature			Overall prostate cancer (752 pairs)			
**Polarity**	***m/z***	**RT (min)**	**OR (95% CI)**	**CLR p-**	**VIP (95% CI)**	**Statistical**
Neg	311.200	6.16	1.00 (0.90 – 1.11)	0.998	2.08 (1.63 – 2.54)	M
Neg	356.184	6.23	1.12 (1.03 – 1.22)	**0.007**	1.65 (1.05 – 2.25)	M
Neg	367.262	6.54	0.80 (0.58 – 1.12)	0.190	1.52 (1.08 – 1.96)	M
Neg	368.265	6.53	0.78 (0.62 – 0.99)	**0.044**	1.69 (1.24 – 2.14)	MU
Neg	381.371	6.79	0.90 (0.82 – 0.99)	**0.036**	1.55 (0.87 – 2.23)	M
Neg	395.387	6.90	0.86 (0.78 – 0.95)	**0.002**	1.52 (0.95 – 2.09)	M
Neg	409.234	6.40	1.01 (0.77 – 1.32)	0.969	1.50 (0.27 – 2.73)	M
Neg	410.237	6.40	1.06 (0.85 – 1.31)	0.612	1.51 (0.48 – 2.54)	M
Neg	412.247	5.95	1.07 (0.98 – 1.18)	0.128	1.60 (1.10 – 2.11)	M
Neg	425.361	5.97	0.85 (0.76 – 0.94)	**0.002**	1.50 (0.73 – 2.27)	M
Neg	480.306	6.40	0.98 (0.78 – 1.23)	0.860	1.54 (0.73 – 2.35)	M
Neg	508.336	6.58	1.08 (0.88 – 1.32)	0.489	1.54 (0.73 – 2.35)	M
Neg	540.327	6.40	0.96 (0.87 – 1.05)	0.344	1.57 (0.54 – 2.61)	M
Neg	541.330	6.40	1.09 (0.89 – 1.34)	0.423	1.71 (0.76 – 2.65)	M
Neg	566.342	6.45	1.10 (0.86 – 1.39)	0.449	1.50 (0.70 – 2.30)	M
Neg	748.521	7.45	0.94 (0.84 – 1.04)	0.207	1.52 (0.91 – 2.13)	M
Pos	201.182	6.25	1.28 (1.07 – 1.54)	**0.008**	1.57 (0.93 – 2.22)	M
Pos	205.083	5.96	1.29 (0.89 – 1.88)	0.175	1.65 (1.25 – 2.04)	M
Pos	229.213	6.50	1.31 (1.09 – 1.57)	**0.003**	1.63 (0.93 – 2.32)	MU
Pos	239.234	6.69	1.17 (1.02 – 1.35)	**0.026**	1.61 (1.02 – 2.20)	M
Pos	257.148	6.11	1.42 (1.11 – 1.81)	**0.005**	2.26 (1.53 – 2.99)	MU
Pos	257.245	6.69	1.39 (1.12 – 1.73)	**0.003**	1.76 (1.28 – 2.24)	MU
Pos	258.248	6.69	1.35 (1.09 – 1.69)	**0.007**	1.70 (1.24 – 2.15)	MU
Pos	301.138	5.95	1.00 (0.70 – 1.43)	0.995	1.62 (1.27 – 1.97)	M
Pos	302.141	5.95	1.18 (0.82 – 1.72)	0.378	1.69 (1.33 – 2.06)	M
Pos	315.190	6.09	1.21 (0.84 – 1.74)	0.313	1.51 (0.81 – 2.22)	M
Pos	357.245	7.24	1.14 (0.91 – 1.42)	0.264	1.89 (1.38 – 2.41)	M
Pos	369.347	7.70	1.28 (1.02 – 1.60)	**0.031**	1.56 (0.83 – 2.28)	MU
Pos	370.252	7.30	1.04 (0.83 – 1.29)	0.740	1.77 (1.09 – 2.45)	M
Pos	371.260	7.51	1.00 (0.86 – 1.15)	0.944	1.63 (0.84 – 2.42)	M
Pos	378.238	7.30	1.06 (0.86 – 1.32)	0.579	2.11 (1.63 – 2.58)	M
Pos	409.749	7.42	1.07 (0.91 – 1.26)	0.444	1.71 (1.00 – 2.41)	M
Pos	421.777	6.60	1.23 (1.03 – 1.46)	**0.022**	1.70 (1.25 – 2.16)	M
Pos	438.293	6.53	1.11 (0.96 – 1.29)	0.166	1.64 (1.08 – 2.19)	M
Pos	500.271	6.16	1.04 (0.95 – 1.15)	0.402	1.51 (0.57 – 2.44)	M
Pos	501.275	6.16	1.00 (0.89 – 1.13)	0.963	1.51 (0.57 – 2.46)	M
Pos	515.306	6.41	1.24 (0.98 – 1.56)	0.076	1.72 (1.06 – 2.38)	M
Pos	515.808	6.41	1.22 (0.96 – 1.54)	0.110	1.60 (0.96 – 2.25)	M
Pos	522.791	6.41	1.19 (0.93 – 1.51)	0.168	2.14 (1.50 – 2.77)	M
Pos	523.794	6.41	1.05 (0.83 – 1.32)	0.710	2.04 (1.42 – 2.65)	M
Pos	547.292	6.32	1.13 (0.94 – 1.35)	0.188	1.62 (0.92 – 2.33)	M
Pos	551.323	6.59	1.14 (0.95 – 1.37)	0.169	1.80 (1.33 – 2.26)	M
Pos	555.406	6.41	0.89 (0.75 – 1.06)	0.185	1.79 (0.34 – 3.25)	M
Pos	564.299	6.19	1.03 (0.92 – 1.14)	0.643	1.64 (0.80 – 2.47)	M
Pos	565.303	6.19	0.99 (0.88 – 1.11)	0.837	1.61 (0.79 – 2.42)	M
Pos	675.536	7.24	1.12 (0.99 – 1.26)	0.080	1.74 (1.07 – 2.41)	M
Pos	676.539	7.24	1.28 (1.09 – 1.51)	**0.003**	1.76 (1.10 – 2.42)	M
Pos	677.541	7.24	1.21 (1.02 – 1.45)	**0.034**	1.74 (1.09 – 2.39)	M
Pos	677.549	7.36	1.25 (1.06 – 1.48)	**0.009**	1.71 (1.09 – 2.33)	M
Pos	689.551	7.37	1.17 (1.00 – 1.35)	**0.047**	1.86 (1.22 – 2.49)	M
Pos	690.554	7.37	1.35 (1.11 – 1.64)	**0.003**	1.84 (1.16 – 2.52)	M
Pos	691.557	7.37	1.29 (1.06 – 1.58)	**0.011**	1.78 (1.21 – 2.36)	M
Pos	692.558	7.38	1.20 (1.00 – 1.44)	**0.050**	1.69 (1.09 – 2.29)	M
Pos	697.517	7.24	1.27 (1.04 – 1.55)	**0.018**	2.14 (1.66 – 2.61)	MU
Pos	698.520	7.24	1.53 (1.17 – 1.99)	**0.002**	2.15 (1.74 – 2.55)	MU
Pos	701.551	7.29	1.13 (1.00 – 1.26)	**0.042**	1.74 (1.05 – 2.43)	M

Pos	702.554	7.29	1.13 (0.99 – 1.29)	0.079	1.72 (1.04 – 2.40)	M
Pos	703.567	7.51	1.11 (0.92 – 1.33)	0.287	2.17 (1.54 – 2.80)	MU
Pos	704.570	7.51	1.14 (0.96 – 1.35)	0.127	2.17 (1.65 – 2.69)	MU
Pos	705.572	7.51	1.37 (1.07 – 1.76)	**0.014**	2.04 (1.50 – 2.58)	MU
Pos	711.532	7.37	1.37 (1.04 – 1.81)	**0.028**	1.96 (1.48 – 2.43)	M
Pos	712.535	7.37	1.30 (1.00 – 1.70)	0.054	1.92 (1.40 – 2.44)	M
Pos	715.565	7.43	1.35 (1.11 – 1.64)	**0.003**	1.85 (1.31 – 2.38)	M
Pos	716.568	7.43	1.34 (1.10 – 1.62)	**0.003**	1.82 (1.28 – 2.36)	MU
Pos	723.531	7.29	1.25 (1.03 – 1.53)	**0.028**	2.08 (1.46 – 2.70)	M
Pos	724.535	7.29	1.37 (1.02 – 1.84)	**0.038**	2.11 (1.60 – 2.63)	M
Pos	725.547	7.51	1.14 (0.94 – 1.40)	0.191	1.77 (0.90 – 2.65)	M
Pos	726.550	7.51	1.17 (0.95 – 1.43)	0.137	1.79 (0.93 – 2.66)	M
Pos	727.565	7.35	1.39 (1.17 – 1.65)	**0.000**	1.58 (1.03 – 2.12)	MU
Pos	728.568	7.35	1.29 (1.09 – 1.52)	**0.003**	1.56 (1.01 – 2.10)	M
Pos	729.581	7.57	1.35 (1.09 – 1.68)	**0.007**	2.09 (1.71 – 2.48)	MU
Pos	730.529	7.36	1.32 (1.13 – 1.54)	**0.001**	1.75 (1.27 – 2.23)	MU
Pos	730.584	7.57	1.62 (1.24 – 2.11)	**0.000**	2.07 (1.72 – 2.42)	MU
Pos	731.532	7.36	1.24 (1.07 – 1.44)	**0.004**	1.74 (1.28 – 2.20)	MU
Pos	731.596	7.83	1.23 (1.01 – 1.50)	**0.041**	1.68 (1.15 – 2.21)	M
Pos	732.544	7.57	1.35 (1.13 – 1.61)	**0.001**	1.54 (0.86 – 2.22)	MU
Pos	732.599	7.83	1.25 (0.96 – 1.63)	0.101	1.69 (1.23 – 2.15)	M
Pos	733.547	7.57	1.33 (1.12 – 1.58)	**0.001**	1.53 (0.83 – 2.22)	MU
Pos	733.602	7.83	1.23 (0.94 – 1.60)	0.127	1.61 (1.04 – 2.17)	M
Pos	744.544	7.50	1.39 (1.13 – 1.70)	**0.001**	2.14 (1.87 – 2.40)	MU
Pos	745.547	7.50	1.39 (1.14 – 1.70)	**0.001**	2.07 (1.74 – 2.39)	MU
Pos	746.559	7.73	1.28 (1.04 – 1.59)	**0.022**	1.55 (1.18 – 1.92)	M
Pos	747.563	7.73	1.24 (1.00 – 1.55)	0.052	1.56 (1.14 – 1.98)	M
Pos	752.511	7.35	1.44 (1.17 – 1.79)	**0.001**	1.68 (1.04 – 2.32)	MU
Pos	756.544	7.45	1.33 (1.11 – 1.59)	**0.002**	1.96 (1.57 – 2.34)	MU
Pos	757.547	7.45	1.31 (1.08 – 1.59)	**0.006**	1.90 (1.49 – 2.30)	MU
Pos	757.611	7.88	1.29 (1.02 – 1.62)	**0.033**	1.77 (1.53 – 2.02)	MU
Pos	758.561	7.66	1.02 (0.91 – 1.14)	0.790	2.29 (1.90 – 2.68)	MU
Pos	759.563	7.66	1.30 (1.10 – 1.54)	**0.002**	2.19 (1.70 – 2.68)	MU
Pos	760.576	7.89	1.24 (0.96 – 1.61)	0.100	1.90 (1.27 – 2.53)	MU
Pos	761.579	7.89	1.38 (1.07 – 1.77)	**0.013**	1.83 (1.29 – 2.37)	MU
Pos	762.581	7.89	1.27 (0.96 – 1.68)	0.090	1.97 (1.45 – 2.50)	M
Pos	763.584	7.89	1.44 (1.07 – 1.94)	**0.018**	2.02 (1.41 – 2.64)	MU
Pos	768.543	7.46	1.32 (1.09 – 1.61)	**0.005**	1.70 (1.25 – 2.16)	MU
Pos	770.558	7.57	1.41 (1.11 – 1.80)	**0.005**	2.03 (1.55 – 2.50)	MU
Pos	770.955	6.41	0.98 (0.85 – 1.13)	0.787	1.91 (1.63 – 2.20)	M
Pos	772.066	7.89	1.03 (0.90 – 1.18)	0.663	1.62 (0.68 – 2.56)	M
Pos	772.574	7.80	1.28 (1.04 – 1.58)	**0.021**	1.85 (1.56 – 2.13)	M
Pos	773.577	7.80	1.29 (1.04 – 1.60)	**0.023**	1.68 (1.29 – 2.06)	M
Pos	783.562	7.59	1.18 (0.94 – 1.47)	0.149	1.74 (1.14 – 2.33)	M
Pos	784.575	7.73	1.14 (0.98 – 1.33)	0.082	2.02 (1.47 – 2.57)	M
Pos	785.578	7.73	1.29 (1.06 – 1.56)	**0.009**	2.22 (1.73 – 2.71)	MU
Pos	786.591	7.98	1.17 (0.98 – 1.39)	0.078	1.96 (1.56 – 2.37)	MU
Pos	787.594	7.98	1.35 (1.10 – 1.67)	**0.005**	1.92 (1.49 – 2.35)	M
Pos	793.547	7.41	1.18 (0.94 – 1.48)	0.145	1.52 (1.24 – 1.79)	M
Pos	821.638	8.47	1.30 (1.03 – 1.64)	**0.030**	1.55 (0.95 – 2.15)	M
Pos	829.561	4.86	1.01 (0.88 – 1.15)	0.940	1.50 (0.76 – 2.24)	M
Pos	829.937	4.86	0.99 (0.86 – 1.14)	0.891	1.50 (0.71 – 2.30)	M
Pos	947.781	4.86	1.02 (0.88 – 1.18)	0.788	1.56 (0.92 – 2.19)	M
Pos	947.925	4.87	1.00 (0.87 – 1.16)	0.955	1.51 (0.87 – 2.14)	M
Pos	948.497	4.86	1.02 (0.88 – 1.18)	0.812	1.51 (0.78 – 2.23)	M
Pos	948.640	4.86	0.99 (0.85 – 1.14)	0.879	1.52 (0.86 – 2.19)	M
Pos	987.625	6.26	1.03 (0.92 – 1.14)	0.646	1.60 (0.98 – 2.22)	M
Pos	991.657	6.41	1.11 (0.98 – 1.24)	0.093	1.51 (0.69 – 2.33)	M
Pos	992.661	6.41	1.06 (0.94 – 1.19)	0.354	1.54 (0.72 – 2.36)	M
Pos	993.663	6.41	1.07 (0.94 – 1.23)	0.310	1.52 (0.71 – 2.34)	M
Pos	994.665	6.41	1.07 (0.93 – 1.24)	0.361	1.55 (0.71 – 2.40)	M

Pos	995.667	6.41	1.05 (0.91 – 1.22)	0.494	1.65 (0.30 – 2.99)	M
Pos	1001.64	6.31	1.07 (0.94 – 1.21)	0.316	1.52 (0.38 – 2.66)	M
Pos	1005.67	6.47	1.06 (0.93 – 1.21)	0.380	1.67 (0.40 – 2.93)	M
Pos	1006.67	6.48	1.01 (0.89 – 1.16)	0.845	1.72 (0.53 – 2.92)	M
Pos	1015.65	6.39	1.09 (0.96 – 1.23)	0.183	1.54 (0.61 – 2.47)	M
Pos	1016.65	6.38	1.05 (0.93 – 1.20)	0.443	1.53 (0.60 – 2.47)	M
Pos	1017.67	6.44	1.07 (0.95 – 1.21)	0.269	1.56 (0.71 – 2.42)	M
Pos	1019.67	6.44	1.08 (0.93 – 1.25)	0.333	1.59 (0.75 – 2.43)	M
Pos	1020.67	6.43	1.04 (0.89 – 1.23)	0.596	1.51 (0.60 – 2.42)	M
Pos	1021.60	6.31	1.04 (0.90 – 1.20)	0.578	1.59 (0.61 – 2.57)	M
Pos	1022.61	6.31	1.07 (0.93 – 1.23)	0.359	1.57 (0.60 – 2.53)	M
Pos	1038.64	6.35	1.01 (0.86 – 1.19)	0.872	1.51 (0.65 – 2.37)	M
Pos	1041.66	6.40	1.09 (0.97 – 1.23)	0.153	1.52 (0.31 – 2.72)	M
Pos	1045.60	6.30	1.01 (0.87 – 1.18)	0.902	1.68 (0.71 – 2.64)	M
Pos	1046.61	6.30	1.05 (0.90 – 1.22)	0.568	1.67 (0.71 – 2.63)	M
Pos	1046.70	6.58	1.06 (0.93 – 1.21)	0.382	1.55 (0.50 – 2.61)	M
Pos	1047.61	6.32	1.00 (0.85 – 1.17)	0.959	1.59 (0.70 – 2.47)	M
Pos	1061.63	6.31	1.10 (0.94 – 1.29)	0.246	1.56 (0.33 – 2.79)	M
Pos	1062.63	6.31	1.07 (0.91 – 1.27)	0.413	1.60 (0.17 – 3.02)	M
Pos	1063.65	6.31	1.03 (0.91 – 1.17)	0.619	1.68 (0.73 – 2.64)	M
Pos	1064.65	6.31	1.05 (0.93 – 1.18)	0.450	1.68 (0.72 – 2.63)	M
Pos	1065.66	6.33	1.05 (0.93 – 1.19)	0.425	1.68 (0.45 – 2.91)	M
Pos	1066.33	7.51	1.03 (0.91 – 1.17)	0.637	1.65 (0.98 – 2.32)	M
Pos	1069.60	6.29	1.00 (0.87 – 1.15)	0.997	1.67 (0.69 – 2.64)	M
Pos	1085.63	6.30	0.91 (0.74 – 1.11)	0.337	1.53 (0.38 – 2.67)	M
Pos	1087.65	6.30	1.03 (0.91 – 1.17)	0.659	1.72 (0.78 – 2.66)	M
Pos	1088.65	6.30	1.03 (0.91 – 1.17)	0.634	1.71 (0.78 – 2.64)	M
Pos	1089.66	6.32	0.97 (0.84 – 1.12)	0.696	1.68 (0.76 – 2.60)	M
Pos	1090.66	6.32	0.98 (0.84 – 1.15)	0.816	1.60 (0.70 – 2.50)	M
Pos	1105.57	4.87	1.02 (0.88 – 1.17)	0.821	1.52 (0.85 – 2.20)	M
**Feature**			**Non–aggressive prostate cancer (587 pairs)**			
**Polarity**	***m/z***	**RT (min)**	**OR (95% CI)**	**CLR p-**	**VIP (95% CI)**	**Statistical**
Neg	311.200	6.16	0.97 (0.87 – 1.09)	0.639	2.01 (1.44 – 2.58)	M
Neg	356.184	6.23	1.10 (1.00 – 1.21)	**0.042**	1.59 (1.14 – 2.04)	M
Neg	368.265	6.53	0.84 (0.64 – 1.09)	0.184	1.65 (1.11 – 2.20)	MU
Neg	409.234	6.40	1.07 (0.79 – 1.45)	0.668	1.54 (0.46 – 2.63)	M
Neg	410.237	6.40	1.09 (0.86 – 1.39)	0.457	1.50 (0.49 – 2.52)	M
Neg	412.247	5.95	1.08 (0.97 – 1.20)	0.149	1.53 (1.07 – 1.99)	M
Neg	508.336	6.58	1.14 (0.91 – 1.43)	0.267	1.59 (0.71 – 2.47)	M
Neg	540.327	6.40	1.17 (0.89 – 1.54)	0.253	1.64 (0.65 – 2.63)	M
Neg	541.330	6.40	1.25 (0.96 – 1.64)	0.100	1.71 (0.92 – 2.51)	M
Neg	568.358	6.58	1.02 (0.80 – 1.30)	0.896	1.50 (0.63 – 2.37)	M
Neg	569.361	6.58	1.03 (0.84 – 1.27)	0.794	1.53 (0.92 – 2.15)	M
Neg	748.521	7.45	0.94 (0.83 – 1.06)	0.278	1.52 (0.87 – 2.16)	M
Pos	205.083	5.96	1.16 (0.77 – 1.74)	0.485	1.57 (1.26 – 1.88)	M
Pos	229.213	6.50	1.27 (1.04 – 1.55)	**0.020**	1.54 (1.02 – 2.06)	M
Pos	239.234	6.69	1.13 (0.97 – 1.32)	0.118	1.50 (1.21 – 1.79)	M
Pos	257.148	6.11	1.33 (1.01 – 1.75)	**0.044**	2.26 (1.58 – 2.95)	MU
Pos	257.245	6.69	1.37 (1.07 – 1.74)	**0.011**	1.68 (1.56 – 1.80)	MU
Pos	258.248	6.69	1.33 (1.04 – 1.71)	**0.021**	1.64 (1.52 – 1.76)	MU
Pos	301.138	5.95	1.02 (0.68 – 1.52)	0.934	1.59 (1.21 – 1.97)	M
Pos	302.141	5.95	1.09 (0.72 – 1.65)	0.701	1.62 (1.21 – 2.02)	M
Pos	315.190	6.09	1.10 (0.74 – 1.65)	0.636	1.60 (1.01 – 2.19)	M
Pos	357.245	7.24	1.13 (0.89 – 1.45)	0.317	1.83 (1.53 – 2.13)	M
Pos	370.252	7.30	1.03 (0.81 – 1.32)	0.790	1.85 (1.48 – 2.21)	M
Pos	370.736	0.74	1.25 (0.96 – 1.63)	0.096	1.64 (1.04 – 2.24)	M
Pos	371.260	7.51	0.99 (0.84 – 1.16)	0.888	1.67 (1.31 – 2.03)	M
Pos	378.238	7.30	1.06 (0.84 – 1.35)	0.606	2.20 (2.01 – 2.39)	M
Pos	379.239	7.30	1.57 (1.15 – 2.15)	**0.005**	1.54 (1.01 – 2.07)	M
Pos	409.749	7.42	1.08 (0.90 – 1.30)	0.397	1.78 (0.93 – 2.64)	M

Pos	421.777	6.60	1.17 (0.97 – 1.40)	0.093	1.58 (1.04 – 2.13)	M
Pos	434.685	0.74	1.41 (1.04 – 1.91)	**0.028**	1.56 (0.92 – 2.21)	M
Pos	438.293	6.53	1.08 (0.92 – 1.27)	0.332	1.59 (0.84 – 2.33)	M
Pos	512.329	6.23	1.01 (0.83 – 1.22)	0.945	1.60 (0.81 – 2.40)	M
Pos	515.306	6.41	1.16 (0.89 – 1.50)	0.275	1.70 (1.29 – 2.11)	M
Pos	515.808	6.41	1.16 (0.90 – 1.49)	0.266	1.53 (0.99 – 2.07)	M
Pos	522.791	6.41	1.14 (0.88 – 1.49)	0.324	2.06 (1.55 – 2.58)	M
Pos	523.794	6.41	1.02 (0.79 – 1.31)	0.903	1.98 (1.47 – 2.50)	M
Pos	539.480	6.27	0.97 (0.83 – 1.13)	0.694	1.51 (0.81 – 2.22)	M
Pos	547.292	6.32	1.15 (0.95 – 1.39)	0.149	1.68 (1.34 – 2.02)	M
Pos	551.323	6.59	1.15 (0.93 – 1.40)	0.193	1.71 (1.09 – 2.33)	M
Pos	555.406	6.41	0.88 (0.72 – 1.06)	0.174	1.74 (0.72 – 2.76)	M
Pos	564.299	6.19	1.07 (0.94 – 1.20)	0.301	1.51 (0.32 – 2.69)	M
Pos	565.303	6.19	1.04 (0.91 – 1.18)	0.589	1.63 (0.43 – 2.84)	M
Pos	570.347	6.35	1.11 (0.93 – 1.32)	0.236	1.55 (0.17 – 2.93)	M
Pos	675.536	7.24	1.12 (0.97 – 1.29)	0.116	1.61 (1.02 – 2.21)	M
Pos	676.539	7.24	1.26 (1.05 – 1.52)	**0.015**	1.63 (1.04 – 2.21)	M
Pos	677.541	7.24	1.23 (1.00 – 1.51)	**0.046**	1.62 (1.11 – 2.13)	M
Pos	677.549	7.36	1.28 (1.05 – 1.56)	**0.013**	1.59 (0.93 – 2.25)	M
Pos	689.551	7.37	1.17 (0.99 – 1.39)	0.074	1.82 (1.00 – 2.63)	M
Pos	690.554	7.37	1.33 (1.07 – 1.66)	**0.012**	1.80 (1.05 – 2.55)	M
Pos	691.557	7.37	1.27 (1.02 – 1.58)	**0.033**	1.76 (1.03 – 2.49)	M
Pos	692.558	7.38	1.20 (0.98 – 1.48)	0.079	1.68 (1.08 – 2.28)	M
Pos	697.517	7.24	1.23 (0.98 – 1.56)	0.079	2.07 (1.53 – 2.61)	MU
Pos	698.520	7.24	1.48 (1.09 – 2.00)	**0.011**	2.07 (1.57 – 2.56)	MU
Pos	701.551	7.29	1.14 (0.99 – 1.30)	0.071	1.66 (1.07 – 2.26)	M
Pos	702.554	7.29	1.15 (0.96 – 1.37)	0.130	1.65 (1.06 – 2.24)	M
Pos	703.567	7.51	1.18 (0.94 – 1.48)	0.153	2.24 (1.54 – 2.95)	MU
Pos	704.570	7.51	1.13 (0.95 – 1.33)	0.167	2.27 (1.57 – 2.96)	MU
Pos	705.572	7.51	1.28 (1.10 – 1.50)	**0.001**	2.14 (1.44 – 2.85)	MU
Pos	706.571	7.51	1.16 (0.94 – 1.42)	0.174	1.52 (0.65 – 2.40)	M
Pos	711.532	7.37	1.24 (0.93 – 1.66)	0.147	2.09 (1.50 – 2.67)	M
Pos	712.535	7.37	1.17 (0.87 – 1.57)	0.301	2.01 (1.29 – 2.73)	M
Pos	715.565	7.43	1.33 (1.07 – 1.65)	**0.010**	1.85 (0.99 – 2.72)	M
Pos	716.568	7.43	1.29 (1.04 – 1.59)	**0.018**	1.86 (1.01 – 2.70)	M
Pos	723.531	7.29	1.23 (0.98 – 1.53)	0.074	2.13 (1.62 – 2.64)	M
Pos	724.535	7.29	1.28 (0.93 – 1.75)	0.126	2.17 (1.63 – 2.71)	M
Pos	725.547	7.51	1.16 (0.93 – 1.44)	0.196	2.03 (1.02 – 3.03)	M
Pos	726.550	7.51	1.16 (0.93 – 1.45)	0.201	2.06 (1.02 – 3.09)	M
Pos	727.565	7.35	1.37 (1.14 – 1.65)	**0.001**	1.56 (0.72 – 2.41)	M
Pos	728.568	7.35	1.25 (1.05 – 1.51)	**0.015**	1.55 (0.82 – 2.28)	M
Pos	729.581	7.57	1.40 (1.08 – 1.82)	**0.012**	2.17 (1.62 – 2.72)	MU
Pos	730.529	7.36	1.34 (1.12 – 1.60)	**0.002**	1.60 (1.15 – 2.05)	M
Pos	730.584	7.57	1.61 (1.19 – 2.17)	**0.002**	2.15 (1.58 – 2.72)	MU
Pos	731.532	7.36	1.28 (1.07 – 1.53)	**0.007**	1.59 (1.21 – 1.97)	M
Pos	731.596	7.83	1.25 (0.98 – 1.59)	0.075	1.75 (0.97 – 2.53)	M
Pos	732.599	7.83	1.23 (0.91 – 1.68)	0.183	1.71 (1.06 – 2.35)	M
Pos	733.602	7.83	1.23 (0.91 – 1.65)	0.180	1.59 (0.99 – 2.19)	M
Pos	734.560	7.84	1.49 (1.08 – 2.05)	**0.015**	1.53 (1.11 – 1.95)	M
Pos	744.544	7.50	1.38 (1.10 – 1.72)	**0.005**	2.11 (1.57 – 2.66)	M
Pos	745.547	7.50	1.40 (1.12 – 1.74)	**0.003**	2.08 (1.63 – 2.53)	MU
Pos	747.563	7.73	1.23 (0.97 – 1.57)	0.091	1.50 (1.08 – 1.93)	M
Pos	752.511	7.35	1.42 (1.12 – 1.80)	**0.004**	1.66 (1.39 – 1.94)	M
Pos	756.544	7.45	1.31 (1.07 – 1.60)	**0.008**	1.89 (1.61 – 2.17)	M
Pos	757.547	7.45	1.31 (1.06 – 1.62)	**0.012**	1.85 (1.61 – 2.09)	MU
Pos	757.611	7.88	1.27 (0.98 – 1.64)	0.067	1.88 (1.21 – 2.56)	M
Pos	758.561	7.66	1.00 (0.88 – 1.15)	0.968	2.31 (1.93 – 2.69)	M
Pos	759.563	7.66	1.25 (1.05 – 1.49)	**0.013**	2.20 (1.83 – 2.57)	MU
Pos	760.576	7.89	1.25 (0.94 – 1.67)	0.119	1.91 (1.44 – 2.39)	M
Pos	761.579	7.89	1.53 (1.12 – 2.10)	**0.008**	1.92 (1.30 – 2.53)	MU
Pos	762.581	7.89	1.28 (0.93 – 1.77)	0.130	1.98 (1.58 – 2.38)	M

Pos	763.584	7.89	1.35 (0.97 – 1.88)	0.074	2.01 (1.49 – 2.53)	M
Pos	768.543	7.46	1.34 (1.08 – 1.66)	**0.008**	1.69 (1.33 – 2.04)	M
Pos	770.558	7.57	1.49 (1.13 – 1.95)	**0.004**	2.02 (1.72 – 2.32)	MU
Pos	770.955	6.41	0.95 (0.82 – 1.11)	0.524	1.79 (1.22 – 2.36)	M
Pos	772.066	7.89	1.04 (0.89 – 1.20)	0.646	1.61 (1.03 – 2.19)	M
Pos	772.574	7.80	1.28 (1.02 – 1.61)	**0.036**	1.91 (1.11 – 2.71)	M
Pos	773.577	7.80	1.31 (1.02 – 1.67)	**0.033**	1.77 (1.03 – 2.51)	M
Pos	782.559	7.60	1.21 (1.01 – 1.44)	**0.039**	1.50 (0.82 – 2.18)	M
Pos	783.562	7.59	1.21 (0.94 – 1.54)	0.136	1.75 (1.19 – 2.31)	M
Pos	784.575	7.73	1.09 (0.94 – 1.27)	0.249	1.95 (1.65 – 2.26)	M
Pos	785.578	7.73	1.24 (1.01 – 1.53)	**0.040**	2.21 (1.94 – 2.49)	M
Pos	786.591	7.98	1.25 (1.00 – 1.56)	**0.047**	1.92 (1.38 – 2.47)	M
Pos	787.594	7.98	1.37 (1.07 – 1.75)	**0.011**	1.88 (1.22 – 2.53)	M
Pos	821.638	8.47	1.28 (0.99 – 1.66)	0.059	1.58 (0.99 – 2.17)	M
Pos	828.539	7.54	1.18 (0.97 – 1.43)	0.096	1.51 (0.37 – 2.65)	M
Pos	829.543	7.54	1.19 (0.98 – 1.44)	0.085	1.51 (0.66 – 2.35)	M
Pos	829.561	4.86	1.08 (0.93 – 1.25)	0.321	1.57 (0.46 – 2.68)	M
Pos	829.686	4.87	1.04 (0.89 – 1.23)	0.624	1.59 (0.45 – 2.74)	M
Pos	829.811	4.86	1.05 (0.90 – 1.23)	0.537	1.56 (0.37 – 2.76)	M
Pos	829.937	4.86	1.04 (0.89 – 1.22)	0.624	1.59 (0.44 – 2.75)	M
Pos	833.638	8.32	0.99 (0.77 – 1.26)	0.912	1.54 (0.94 – 2.13)	M
Pos	947.781	4.86	1.08 (0.92 – 1.28)	0.346	1.62 (0.59 – 2.65)	M
Pos	947.925	4.87	1.03 (0.88 – 1.20)	0.705	1.60 (0.48 – 2.72)	M
Pos	948.067	4.87	1.08 (0.91 – 1.28)	0.407	1.54 (0.37 – 2.70)	M
Pos	948.354	4.86	1.12 (0.96 – 1.29)	0.142	1.52 (0.48 – 2.56)	M
Pos	948.497	4.86	1.04 (0.88 – 1.23)	0.643	1.62 (0.58 – 2.66)	M
Pos	948.640	4.86	1.06 (0.90 – 1.25)	0.482	1.59 (0.47 – 2.72)	M
Pos	949.610	6.41	1.11 (0.94 – 1.30)	0.214	1.60 (0.59 – 2.60)	M
Pos	975.626	6.44	1.10 (0.92 – 1.31)	0.303	1.61 (0.46 – 2.77)	M
Pos	976.631	6.44	1.08 (0.91 – 1.29)	0.355	1.53 (0.32 – 2.74)	M
Pos	987.625	6.26	1.06 (0.94 – 1.20)	0.336	1.72 (0.60 – 2.84)	M
Pos	991.657	6.41	1.09 (0.96 – 1.24)	0.204	1.78 (0.27 – 3.28)	M
Pos	992.661	6.41	1.05 (0.92 – 1.20)	0.454	1.80 (0.26 – 3.34)	M
Pos	993.663	6.41	1.07 (0.92 – 1.24)	0.383	1.80 (0.27 – 3.34)	M
Pos	994.665	6.41	1.06 (0.90 – 1.25)	0.469	1.82 (0.25 – 3.39)	M
Pos	995.667	6.41	1.04 (0.88 – 1.23)	0.646	1.69 (0.25 – 3.14)	M
Pos	1001.64	6.31	1.09 (0.95 – 1.25)	0.221	1.53 (0.10 – 2.96)	M
Pos	1003.65	6.40	1.15 (0.93 – 1.41)	0.207	1.77 (0.25 – 3.28)	M
Pos	1005.67	6.47	1.07 (0.92 – 1.23)	0.392	1.66 (0.30 – 3.02)	M
Pos	1006.67	6.48	1.04 (0.90 – 1.20)	0.636	1.73 (0.34 – 3.12)	M
Pos	1013.63	6.40	1.10 (0.85 – 1.42)	0.484	1.61 (0.82 – 2.40)	M
Pos	1014.64	6.40	1.05 (0.82 – 1.35)	0.682	1.72 (0.97 – 2.48)	M
Pos	1015.65	6.39	1.10 (0.96 – 1.26)	0.156	1.76 (0.59 – 2.93)	M
Pos	1016.65	6.38	1.07 (0.93 – 1.23)	0.371	1.75 (0.55 – 2.94)	M
Pos	1017.67	6.44	1.08 (0.95 – 1.23)	0.261	1.81 (0.48 – 3.15)	M
Pos	1019.67	6.44	1.09 (0.92 – 1.29)	0.342	1.85 (0.48 – 3.21)	M
Pos	1020.67	6.43	1.06 (0.88 – 1.26)	0.563	1.77 (0.70 – 2.85)	M
Pos	1021.60	6.31	1.06 (0.91 – 1.23)	0.454	1.66 (0.23 – 3.09)	M
Pos	1022.61	6.31	1.09 (0.93 – 1.28)	0.289	1.62 (0.02 – 3.21)	M
Pos	1031.68	6.47	1.09 (0.95 – 1.26)	0.216	1.60 (0.67 – 2.53)	M
Pos	1032.68	6.47	1.05 (0.90 – 1.22)	0.539	1.68 (0.64 – 2.72)	M
Pos	1037.63	6.35	1.10 (0.93 – 1.29)	0.281	1.66 (1.05 – 2.26)	M
Pos	1038.64	6.35	1.04 (0.88 – 1.24)	0.642	1.67 (1.00 – 2.34)	M
Pos	1040.65	6.32	1.07 (0.95 – 1.19)	0.270	1.58 (0.25 – 2.91)	M
Pos	1041.66	6.40	1.09 (0.96 – 1.25)	0.185	1.68 (0.92 – 2.45)	M
Pos	1042.67	6.40	1.08 (0.94 – 1.25)	0.288	1.58 (0.78 – 2.39)	M
Pos	1044.68	6.45	1.10 (0.95 – 1.28)	0.214	1.65 (0.16 – 3.14)	M
Pos	1045.60	6.30	1.03 (0.87 – 1.21)	0.766	1.77 (0.32 – 3.23)	M
Pos	1045.69	6.58	1.09 (0.94 – 1.25)	0.256	1.60 (0.58 – 2.63)	M
Pos	1046.61	6.30	1.08 (0.91 – 1.27)	0.384	1.78 (0.28 – 3.28)	M
Pos	1046.70	6.58	1.09 (0.95 – 1.26)	0.217	1.53 (0.52 – 2.55)	M

Pos	1047.61	6.32	1.01 (0.85 – 1.20)	0.919	1.70 (0.43 – 2.97)	M
Pos	1047.71	6.59	1.03 (0.91 – 1.17)	0.644	1.82 (0.47 – 3.18)	M
Pos	1049.72	6.59	1.01 (0.88 – 1.17)	0.859	1.83 (0.54 – 3.12)	M
Pos	1050.72	6.59	1.02 (0.90 – 1.17)	0.741	1.84 (0.70 – 2.98)	M
Pos	1061.63	6.31	1.13 (0.95 – 1.35)	0.175	1.55 (0.73 – 2.38)	M
Pos	1062.63	6.31	1.11 (0.92 – 1.33)	0.290	1.51 (0.64 – 2.38)	M
Pos	1063.65	6.31	1.06 (0.93 – 1.20)	0.398	1.80 (0.55 – 3.05)	M
Pos	1064.65	6.31	1.07 (0.93 – 1.22)	0.343	1.78 (0.50 – 3.06)	M
Pos	1065.66	6.33	1.08 (0.94 – 1.24)	0.277	1.59 (0.23 – 2.95)	M
Pos	1066.33	7.51	1.04 (0.90 – 1.19)	0.629	1.56 (1.11 – 2.01)	M
Pos	1066.66	6.34	1.07 (0.91 – 1.24)	0.421	1.52 (0.98 – 2.06)	M
Pos	1068.68	6.43	1.03 (0.86 – 1.23)	0.753	1.55 (0.54 – 2.56)	M
Pos	1069.60	6.29	0.99 (0.85 – 1.16)	0.933	1.77 (0.60 – 2.94)	M
Pos	1085.63	6.30	0.98 (0.80 – 1.20)	0.828	1.65 (0.85 – 2.44)	M
Pos	1087.65	6.30	1.04 (0.90 – 1.19)	0.605	1.87 (0.67 – 3.07)	M
Pos	1088.65	6.30	1.04 (0.91 – 1.19)	0.553	1.85 (0.63 – 3.07)	M
Pos	1089.66	6.32	1.01 (0.87 – 1.18)	0.872	1.82 (0.56 – 3.08)	M
Pos	1090.66	6.32	1.02 (0.86 – 1.20)	0.836	1.72 (0.61 – 2.83)	M
Pos	1105.57	4.87	1.05 (0.90 – 1.23)	0.536	1.61 (0.49 – 2.73)	M
Pos	1105.74	4.86	1.04 (0.89 – 1.22)	0.602	1.52 (0.44 – 2.59)	M
Pos	1106.07	4.86	1.09 (0.94 – 1.27)	0.270	1.51 (0.48 – 2.53)	M
**Feature**			**Younger subgroup of 40- and 50-year-olds at baseline (326**			
**Polarity**	***m/z***	**RT (min)**	**OR (95% CI)**	**CLR p-**	**VIP (95% CI)**	**Statistical**
Neg	279.231	6.32	1.22 (0.97 – 1.52)	0.084	1.87 (0.99 – 2.76)	M
Neg	620.593	8.87	1.01 (0.86 – 1.19)	0.877	1.70 (1.04 – 2.35)	M
Neg	645.596	8.50	0.94 (0.74 – 1.18)	0.584	1.59 (0.81 – 2.38)	M
Neg	750.537	7.68	1.02 (0.81 – 1.27)	0.891	1.68 (0.83 – 2.53)	M
Neg	857.663	8.67	0.96 (0.83 – 1.11)	0.569	2.07 (1.59 – 2.55)	MU
Neg	887.673	8.69	0.83 (0.67 – 1.03)	0.089	1.58 (1.03 – 2.14)	MU
Pos	135.001	1.05	1.30 (0.85 – 2.00)	0.225	1.96 (1.06 – 2.86)	M
Pos	158.962	0.64	5.13 (1.95 –	**0.001**	1.83 (1.41 – 2.25)	M
Pos	162.048	0.93	1.07 (0.71 – 1.61)	0.761	1.59 (0.77 – 2.41)	M
Pos	176.063	1.54	1.32 (0.95 – 1.82)	0.098	2.05 (1.56 – 2.54)	M
Pos	201.182	6.25	1.24 (0.98 – 1.58)	0.073	2.09 (1.61 – 2.56)	M
Pos	203.050	0.73	1.33 (0.76 – 2.34)	0.319	1.86 (1.19 – 2.53)	M
Pos	206.891	0.69	3.13 (1.23 – 7.96)	**0.017**	2.18 (1.37 – 3.00)	M
Pos	208.888	0.69	2.57 (1.06 – 6.24)	**0.037**	2.16 (1.30 – 3.02)	M
Pos	211.106	5.92	1.24 (0.93 – 1.64)	0.146	1.85 (1.59 – 2.12)	M
Pos	216.920	0.68	5.49 (1.56 – 19.3)	**0.008**	2.14 (1.09 – 3.18)	MU
Pos	218.917	0.68	3.33 (1.17 – 9.43)	**0.024**	2.17 (1.20 – 3.14)	M
Pos	226.042	1.16	1.41 (0.94 – 2.12)	0.093	2.02 (1.13 – 2.90)	MU
Pos	226.949	0.64	4.16 (1.50 –	**0.006**	2.37 (1.79 – 2.95)	MU
Pos	227.076	2.25	1.34 (0.89 – 2.01)	0.162	2.00 (1.06 – 2.94)	MU
Pos	229.213	6.50	1.14 (0.89 – 1.47)	0.294	1.75 (1.38 – 2.13)	M
Pos	257.148	6.11	0.96 (0.67 – 1.39)	0.836	2.56 (1.67 – 3.46)	MU
Pos	257.245	6.69	1.06 (0.77 – 1.46)	0.730	1.78 (1.48 – 2.08)	M
Pos	258.248	6.69	1.06 (0.77 – 1.47)	0.704	1.65 (1.23 – 2.07)	M
Pos	263.082	0.72	1.85 (1.20 – 2.83)	**0.005**	1.85 (1.20 – 2.49)	M
Pos	265.249	6.73	1.08 (0.87 – 1.34)	0.502	1.64 (1.31 – 1.97)	M
Pos	267.169	5.92	1.21 (0.89 – 1.64)	0.232	1.81 (1.36 – 2.27)	M
Pos	270.955	0.96	1.13 (0.75 – 1.71)	0.556	2.04 (1.34 – 2.75)	M
Pos	272.952	0.96	1.05 (0.72 – 1.54)	0.809	2.11 (1.49 – 2.73)	M
Pos	283.260	6.73	1.10 (0.89 – 1.36)	0.401	1.63 (1.30 – 1.97)	M
Pos	284.263	6.73	1.10 (0.89 – 1.37)	0.384	1.62 (1.14 – 2.10)	M
Pos	285.275	6.91	1.07 (0.80 – 1.41)	0.663	1.74 (1.42 – 2.05)	M
Pos	289.151	5.92	1.17 (0.88 – 1.55)	0.290	1.92 (1.55 – 2.29)	M
Pos	293.242	6.97	1.08 (0.81 – 1.44)	0.618	1.51 (0.90 – 2.12)	M
Pos	303.226	6.61	1.23 (1.00 – 1.51)	**0.048**	2.06 (1.26 – 2.86)	MU
Pos	305.242	6.73	1.19 (0.95 – 1.49)	0.138	1.84 (1.32 – 2.36)	MU
Pos	306.245	6.73	1.14 (0.91 – 1.41)	0.253	1.74 (1.08 – 2.39)	M

Pos	315.190	6.09	0.84 (0.52 – 1.35)	0.459	1.74 (1.17 – 2.31)	M
Pos	326.242	5.92	1.11 (0.83 – 1.47)	0.489	1.52 (0.66 – 2.38)	M
Pos	337.164	6.61	1.16 (0.93 – 1.45)	0.183	1.71 (0.93 – 2.49)	M
Pos	342.865	0.68	1.52 (0.81 – 2.86)	0.195	1.92 (1.02 – 2.82)	M
Pos	352.893	0.67	1.71 (0.86 – 3.38)	0.124	2.11 (1.23 – 2.99)	M
Pos	369.347	7.70	0.91 (0.63 – 1.30)	0.591	1.63 (1.38 – 1.88)	M
Pos	370.736	0.74	1.11 (0.73 – 1.69)	0.612	1.68 (1.40 – 1.96)	M
Pos	372.733	0.74	1.13 (0.71 – 1.80)	0.606	1.53 (0.76 – 2.30)	M
Pos	374.730	0.74	0.87 (0.56 – 1.35)	0.522	1.61 (0.82 – 2.39)	M
Pos	379.239	7.30	1.37 (0.92 – 2.04)	0.125	2.13 (1.69 – 2.58)	M
Pos	383.112	0.73	1.39 (0.85 – 2.27)	0.193	1.79 (1.00 – 2.58)	M
Pos	384.115	0.73	1.28 (0.81 – 2.02)	0.286	1.64 (0.92 – 2.36)	MU
Pos	390.794	0.68	1.39 (0.84 – 2.29)	0.199	1.82 (1.38 – 2.26)	M
Pos	392.791	0.68	1.46 (0.79 – 2.70)	0.223	2.12 (1.46 – 2.79)	MU
Pos	400.823	0.68	1.65 (0.89 – 3.07)	0.115	2.20 (1.59 – 2.80)	M
Pos	402.820	0.68	1.86 (1.00 – 3.45)	**0.048**	2.09 (1.13 – 3.04)	MU
Pos	410.851	0.67	1.34 (0.83 – 2.15)	0.231	1.93 (1.07 – 2.78)	M
Pos	412.848	0.67	1.43 (1.07 – 1.92)	**0.016**	1.80 (1.11 – 2.49)	M
Pos	420.880	0.67	1.39 (1.06 – 1.81)	**0.017**	1.84 (1.47 – 2.20)	M
Pos	421.777	6.60	1.12 (0.89 – 1.42)	0.331	1.63 (1.01 – 2.24)	M
Pos	430.691	0.74	0.98 (0.67 – 1.43)	0.902	1.62 (0.82 – 2.41)	M
Pos	432.688	0.74	1.04 (0.68 – 1.60)	0.862	1.81 (0.97 – 2.66)	M
Pos	434.685	0.74	1.21 (0.77 – 1.89)	0.413	2.00 (1.30 – 2.71)	M
Pos	460.778	0.68	1.36 (0.89 – 2.07)	0.151	1.66 (1.08 – 2.24)	MU
Pos	466.324	6.71	1.12 (0.89 – 1.41)	0.348	1.64 (0.73 – 2.55)	M
Pos	480.337	6.52	0.99 (0.77 – 1.29)	0.955	1.70 (1.16 – 2.25)	M
Pos	488.649	0.74	1.17 (0.82 – 1.69)	0.388	2.02 (1.19 – 2.85)	M
Pos	490.646	0.74	1.15 (0.80 – 1.66)	0.452	1.97 (1.16 – 2.78)	M
Pos	506.353	6.58	0.94 (0.67 – 1.33)	0.732	1.50 (1.25 – 1.75)	M
Pos	524.368	6.59	1.10 (0.81 – 1.50)	0.529	1.63 (1.27 – 2.00)	M
Pos	525.370	6.59	1.14 (0.83 – 1.57)	0.412	1.53 (1.34 – 1.72)	M
Pos	526.371	6.59	1.24 (0.89 – 1.75)	0.209	1.53 (1.29 – 1.77)	M
Pos	539.496	6.72	1.15 (0.96 – 1.39)	0.140	1.83 (1.43 – 2.24)	M
Pos	555.406	6.41	0.70 (0.53 – 0.93)	**0.014**	1.87 (1.44 – 2.29)	M
Pos	565.512	6.73	1.06 (0.90 – 1.25)	0.477	1.53 (1.05 – 2.01)	M
Pos	570.347	6.35	0.95 (0.74 – 1.23)	0.705	1.68 (0.93 – 2.44)	M
Pos	571.351	6.35	0.97 (0.73 – 1.28)	0.814	1.57 (0.97 – 2.18)	M
Pos	621.431	6.74	1.54 (1.06 – 2.24)	**0.023**	1.59 (0.99 – 2.19)	M
Pos	661.519	7.12	1.24 (0.98 – 1.59)	0.080	1.51 (0.97 – 2.04)	M
Pos	673.519	7.05	1.28 (0.99 – 1.66)	0.064	1.53 (0.98 – 2.09)	M
Pos	674.522	7.05	1.20 (0.93 – 1.56)	0.165	1.54 (0.95 – 2.12)	M
Pos	675.536	7.24	1.11 (0.82 – 1.49)	0.502	1.77 (1.16 – 2.39)	M
Pos	676.539	7.24	1.17 (0.85 – 1.62)	0.333	1.79 (1.21 – 2.38)	M
Pos	677.541	7.24	1.16 (0.83 – 1.62)	0.381	1.67 (1.04 – 2.29)	M
Pos	677.549	7.36	1.12 (0.81 – 1.53)	0.498	1.90 (1.39 – 2.41)	M
Pos	687.535	7.17	1.19 (0.91 – 1.54)	0.202	1.51 (0.94 – 2.07)	M
Pos	689.551	7.37	1.01 (0.72 – 1.41)	0.974	1.65 (1.00 – 2.30)	M
Pos	690.554	7.37	1.08 (0.76 – 1.54)	0.654	1.62 (0.94 – 2.31)	M
Pos	691.557	7.37	1.06 (0.76 – 1.47)	0.752	1.59 (0.97 – 2.21)	M
Pos	697.517	7.24	1.00 (0.66 – 1.52)	0.987	1.88 (1.22 – 2.54)	M
Pos	698.520	7.24	1.06 (0.71 – 1.58)	0.781	1.82 (1.15 – 2.50)	M
Pos	699.525	7.24	1.43 (0.99 – 2.08)	0.060	1.59 (1.39 – 1.78)	M
Pos	701.551	7.29	1.03 (0.76 – 1.41)	0.844	1.79 (1.09 – 2.48)	M
Pos	702.554	7.29	1.13 (0.80 – 1.61)	0.484	1.78 (1.06 – 2.49)	M
Pos	703.567	7.51	0.87 (0.58 – 1.30)	0.486	1.83 (0.81 – 2.85)	M
Pos	704.570	7.51	0.87 (0.62 – 1.21)	0.398	1.67 (0.45 – 2.88)	M
Pos	705.572	7.51	0.96 (0.58 – 1.57)	0.861	1.77 (0.78 – 2.77)	M
Pos	715.565	7.43	1.27 (0.91 – 1.77)	0.154	1.83 (0.93 – 2.74)	M
Pos	716.568	7.43	1.14 (0.86 – 1.53)	0.367	1.84 (1.16 – 2.51)	M
Pos	727.565	7.35	1.43 (1.06 – 1.94)	**0.020**	1.75 (0.76 – 2.74)	M
Pos	728.568	7.35	1.35 (0.99 – 1.83)	0.056	1.69 (0.74 – 2.63)	M

Pos	729.581	7.57	1.00 (0.67 – 1.50)	0.995	1.67 (0.00 – 3.35)	M
Pos	730.529	7.36	1.21 (0.93 – 1.56)	0.155	1.66 (0.99 – 2.33)	M
Pos	731.532	7.36	1.21 (0.92 – 1.61)	0.179	1.67 (0.97 – 2.38)	M
Pos	744.544	7.50	1.19 (0.89 – 1.59)	0.232	1.95 (1.28 – 2.62)	M
Pos	745.547	7.50	1.25 (0.92 – 1.71)	0.159	1.97 (1.43 – 2.51)	M
Pos	752.511	7.35	1.22 (0.89 – 1.66)	0.212	1.57 (0.92 – 2.22)	M
Pos	756.544	7.45	1.18 (0.90 – 1.55)	0.223	1.97 (1.42 – 2.52)	M
Pos	757.547	7.45	1.18 (0.88 – 1.58)	0.272	1.95 (1.44 – 2.47)	M
Pos	758.561	7.66	0.92 (0.75 – 1.13)	0.442	1.63 (0.73 – 2.53)	M
Pos	759.563	7.66	1.00 (0.80 – 1.25)	0.986	1.70 (0.96 – 2.44)	M
Pos	770.558	7.57	1.06 (0.75 – 1.49)	0.745	1.73 (0.91 – 2.55)	M
Pos	786.591	7.98	1.02 (0.80 – 1.30)	0.866	1.86 (1.13 – 2.60)	M
Pos	787.594	7.98	1.03 (0.77 – 1.38)	0.831	1.89 (1.22 – 2.57)	M
Pos	792.542	7.41	0.99 (0.75 – 1.29)	0.924	1.52 (0.62 – 2.42)	M
Pos	829.543	7.54	0.99 (0.77 – 1.28)	0.927	1.59 (0.89 – 2.29)	M
Pos	832.572	7.59	1.05 (0.82 – 1.36)	0.697	1.52 (1.10 – 1.93)	M
Pos	992.661	6.41	1.01 (0.86 – 1.19)	0.867	1.51 (0.80 – 2.23)	M
Pos	993.663	6.41	1.02 (0.82 – 1.25)	0.892	1.56 (0.82 – 2.30)	M
Pos	994.665	6.41	1.01 (0.80 – 1.28)	0.930	1.56 (0.85 – 2.28)	M
Pos	995.667	6.41	0.94 (0.74 – 1.20)	0.627	1.52 (0.76 – 2.29)	M
Pos	1017.67	6.44	1.07 (0.90 – 1.26)	0.448	1.53 (0.74 – 2.32)	M
Pos	1019.67	6.44	1.04 (0.80 – 1.35)	0.791	1.65 (0.71 – 2.60)	M
Pos	1020.67	6.43	0.97 (0.75 – 1.26)	0.840	1.66 (0.66 – 2.67)	M
Pos	1021.60	6.31	1.08 (0.85 – 1.37)	0.532	1.53 (1.18 – 1.88)	M
Pos	1022.61	6.31	1.13 (0.88 – 1.46)	0.349	1.70 (0.93 – 2.47)	M
Pos	1045.60	6.30	1.00 (0.78 – 1.29)	0.993	1.59 (1.09 – 2.10)	M
Pos	1046.61	6.30	1.04 (0.78 – 1.37)	0.807	1.58 (1.01 – 2.16)	M
Pos	1047.61	6.32	0.96 (0.73 – 1.26)	0.753	1.66 (1.19 – 2.13)	M
Pos	1050.72	6.59	0.83 (0.67 – 1.03)	0.094	1.55 (0.91 – 2.18)	M
Pos	1061.63	6.31	0.99 (0.75 – 1.31)	0.939	1.51 (0.81 – 2.22)	M
Pos	1062.63	6.31	0.89 (0.64 – 1.24)	0.488	1.51 (1.00 – 2.02)	M
Pos	1063.65	6.31	1.04 (0.86 – 1.26)	0.715	1.60 (1.12 – 2.08)	M
Pos	1064.65	6.31	1.05 (0.85 – 1.29)	0.654	1.59 (1.15 – 2.03)	M
Pos	1065.66	6.33	1.06 (0.85 – 1.31)	0.608	1.56 (1.15 – 1.97)	M
Pos	1069.60	6.29	0.95 (0.75 – 1.22)	0.708	1.74 (0.84 – 2.65)	M
Pos	1085.63	6.30	0.73 (0.51 – 1.06)	0.103	1.74 (0.60 – 2.87)	M
Pos	1087.65	6.30	0.86 (0.66 – 1.12)	0.267	1.66 (0.89 – 2.43)	M
Pos	1088.65	6.30	0.96 (0.76 – 1.21)	0.743	1.64 (0.88 – 2.41)	M
Pos	1089.66	6.32	0.98 (0.77 – 1.25)	0.863	1.68 (0.95 – 2.42)	M
Pos	1090.66	6.32	0.95 (0.72 – 1.25)	0.706	1.58 (0.87 – 2.30)	M
**Feature**			**Older subgroup of 60-year-olds at baseline (426 pairs)**			
**Polarity**	***m/z***	**RT (min)**	**OR (95% CI)**	**CLR p-**	**VIP (95% CI)**	**Statistical**
Neg	179.055	0.72	0.54 (0.32 – 0.91)	**0.022**	1.56 (0.69 – 2.43)	M
Neg	311.200	6.16	1.29 (1.10 – 1.52)	**0.002**	2.33 (1.91 – 2.75)	MU
Neg	356.184	6.23	1.23 (1.10 – 1.36)	**0.000**	1.79 (1.32 – 2.26)	MU
Neg	367.262	6.54	0.50 (0.32 – 0.79)	**0.003**	1.80 (1.21 – 2.40)	M
Neg	368.265	6.53	0.54 (0.37 – 0.79)	**0.002**	1.74 (1.21 – 2.26)	MU
Neg	409.234	6.40	0.99 (0.70 – 1.41)	0.950	1.67 (0.90 – 2.43)	M
Neg	410.237	6.40	0.97 (0.71 – 1.32)	0.834	1.67 (1.02 – 2.33)	M
Neg	412.247	5.95	1.18 (1.04 – 1.33)	**0.008**	1.81 (1.45 – 2.17)	MU
Neg	480.306	6.40	0.83 (0.61 – 1.14)	0.250	1.64 (0.57 – 2.71)	M
Neg	508.336	6.58	1.00 (0.76 – 1.32)	0.981	1.50 (0.98 – 2.03)	M
Neg	540.327	6.40	1.06 (0.81 – 1.38)	0.677	1.72 (0.82 – 2.62)	M
Neg	541.330	6.40	1.02 (0.80 – 1.31)	0.851	1.79 (0.76 – 2.82)	M
Neg	748.521	7.45	0.89 (0.79 – 1.00)	0.057	1.65 (1.21 – 2.08)	M
Neg	778.587	6.40	1.28 (1.05 – 1.56)	**0.013**	1.88 (1.49 – 2.27)	M
Pos	191.015	0.96	1.56 (1.20 – 2.03)	**0.001**	1.54 (0.57 – 2.52)	M
Pos	205.083	5.96	2.30 (1.35 – 3.93)	**0.002**	1.81 (1.33 – 2.30)	M
Pos	239.234	6.69	1.33 (1.10 – 1.60)	**0.003**	1.53 (0.97 – 2.09)	M
Pos	257.148	6.11	1.92 (1.36 – 2.70)	**0.000**	2.29 (1.92 – 2.66)	MU

Pos	257.245	6.69	1.75 (1.28 – 2.38)	**0.000**	1.64 (1.18 – 2.10)	MU
Pos	258.248	6.69	1.66 (1.22 – 2.26)	**0.001**	1.58 (1.15 – 2.00)	M
Pos	301.138	5.95	1.92 (1.15 – 3.21)	**0.013**	1.74 (1.40 – 2.07)	M
Pos	302.141	5.95	2.57 (1.47 – 4.49)	**0.001**	1.80 (1.50 – 2.10)	M
Pos	357.245	7.24	1.98 (1.37 – 2.87)	**0.000**	1.86 (1.48 – 2.24)	M
Pos	370.252	7.30	1.66 (1.15 – 2.40)	**0.007**	1.76 (1.22 – 2.30)	M
Pos	371.260	7.51	1.24 (0.99 – 1.56)	0.061	1.59 (0.83 – 2.35)	M
Pos	378.238	7.30	1.75 (1.24 – 2.47)	**0.001**	2.02 (1.52 – 2.52)	M
Pos	380.252	6.23	1.37 (1.11 – 1.67)	**0.003**	1.51 (1.01 – 2.01)	M
Pos	409.749	7.42	1.63 (1.21 – 2.21)	**0.002**	1.70 (1.40 – 2.00)	M
Pos	421.777	6.60	1.35 (1.04 – 1.75)	**0.024**	1.54 (1.25 – 1.84)	M
Pos	437.319	6.70	1.63 (1.03 – 2.59)	**0.037**	1.69 (1.12 – 2.26)	M
Pos	438.293	6.53	1.36 (1.10 – 1.67)	**0.005**	1.66 (1.17 – 2.15)	M
Pos	496.338	6.41	1.24 (0.85 – 1.80)	0.266	1.54 (0.58 – 2.50)	M
Pos	515.306	6.41	1.48 (1.08 – 2.03)	**0.015**	1.72 (1.31 – 2.14)	M
Pos	515.808	6.41	1.87 (1.24 – 2.80)	**0.003**	1.67 (1.24 – 2.11)	M
Pos	522.791	6.41	2.47 (1.57 – 3.88)	**0.000**	2.30 (1.87 – 2.73)	MU
Pos	523.293	6.41	2.28 (1.54 – 3.39)	**0.000**	2.04 (1.62 – 2.46)	M
Pos	523.794	6.41	2.00 (1.34 – 3.00)	**0.001**	2.16 (1.77 – 2.56)	M
Pos	539.306	6.32	1.50 (1.09 – 2.07)	**0.013**	1.52 (0.94 – 2.10)	M
Pos	547.292	6.32	1.41 (1.07 – 1.85)	**0.013**	1.67 (1.07 – 2.27)	M
Pos	551.323	6.59	1.41 (1.08 – 1.84)	**0.011**	1.89 (1.64 – 2.14)	M
Pos	555.406	6.41	1.09 (0.86 – 1.40)	0.471	1.58 (0.53 – 2.63)	M
Pos	603.527	8.52	1.40 (1.09 – 1.79)	**0.009**	1.64 (1.21 – 2.07)	M
Pos	638.564	8.55	1.40 (1.10 – 1.78)	**0.006**	1.81 (1.31 – 2.32)	MU
Pos	643.519	8.55	1.58 (1.22 – 2.05)	**0.001**	1.75 (1.42 – 2.08)	MU
Pos	644.522	8.55	1.52 (1.16 – 2.00)	**0.003**	1.71 (1.27 – 2.14)	M
Pos	675.536	7.24	1.12 (0.98 – 1.28)	0.106	1.65 (1.13 – 2.18)	M
Pos	676.539	7.24	1.32 (1.09 – 1.61)	**0.006**	1.66 (1.14 – 2.18)	M
Pos	677.541	7.24	1.23 (1.00 – 1.52)	0.052	1.68 (1.23 – 2.13)	M
Pos	677.549	7.36	1.30 (1.07 – 1.59)	**0.009**	1.58 (1.03 – 2.13)	M
Pos	689.551	7.37	1.21 (1.01 – 1.44)	**0.035**	1.80 (1.40 – 2.20)	M
Pos	690.554	7.37	1.48 (1.16 – 1.89)	**0.002**	1.80 (1.44 – 2.16)	M
Pos	691.557	7.37	1.44 (1.12 – 1.86)	**0.005**	1.74 (1.44 – 2.04)	M
Pos	692.558	7.38	1.42 (1.12 – 1.79)	**0.003**	1.65 (1.45 – 1.85)	M
Pos	697.517	7.24	1.37 (1.07 – 1.75)	**0.014**	1.98 (1.69 – 2.28)	M
Pos	698.520	7.24	1.95 (1.37 – 2.79)	**0.000**	2.01 (1.69 – 2.33)	MU
Pos	701.551	7.29	1.14 (1.01 – 1.30)	**0.040**	1.66 (1.29 – 2.03)	M
Pos	702.554	7.29	1.13 (0.98 – 1.30)	0.107	1.65 (1.28 – 2.02)	M
Pos	703.567	7.51	1.20 (0.94 – 1.52)	0.146	2.14 (1.94 – 2.33)	M
Pos	704.570	7.51	1.31 (1.00 – 1.70)	**0.048**	2.14 (1.96 – 2.33)	M
Pos	705.572	7.51	1.56 (1.14 – 2.15)	**0.006**	2.00 (1.83 – 2.17)	M
Pos	707.320	6.80	0.97 (0.85 – 1.10)	0.582	1.50 (0.79 – 2.22)	M
Pos	711.532	7.37	2.05 (1.37 – 3.06)	**0.001**	1.95 (1.53 – 2.37)	M
Pos	712.535	7.37	1.82 (1.25 – 2.64)	**0.002**	1.94 (1.68 – 2.21)	M
Pos	715.565	7.43	1.39 (1.09 – 1.77)	**0.009**	1.77 (1.60 – 1.93)	M
Pos	716.568	7.43	1.49 (1.15 – 1.92)	**0.002**	1.74 (1.55 – 1.94)	M
Pos	723.531	7.29	1.40 (1.07 – 1.83)	**0.014**	2.01 (1.72 – 2.29)	M
Pos	724.535	7.29	1.91 (1.25 – 2.93)	**0.003**	2.05 (1.76 – 2.34)	M
Pos	725.547	7.51	1.30 (1.00 – 1.69)	**0.048**	1.59 (0.55 – 2.62)	M
Pos	726.550	7.51	1.37 (1.05 – 1.79)	**0.022**	1.67 (0.63 – 2.72)	M
Pos	727.565	7.35	1.37 (1.12 – 1.68)	**0.003**	1.51 (1.26 – 1.76)	M
Pos	728.568	7.35	1.26 (1.03 – 1.54)	**0.023**	1.51 (0.64 – 2.38)	M
Pos	729.581	7.57	1.56 (1.14 – 2.12)	**0.005**	2.04 (1.78 – 2.31)	MU
Pos	730.529	7.36	1.38 (1.13 – 1.68)	**0.002**	1.65 (1.34 – 1.97)	M
Pos	730.584	7.57	2.23 (1.54 – 3.22)	**0.000**	2.01 (1.69 – 2.33)	MU
Pos	731.532	7.36	1.25 (1.05 – 1.49)	**0.011**	1.62 (1.29 – 1.95)	M
Pos	731.596	7.83	1.49 (1.12 – 1.98)	**0.007**	1.70 (1.25 – 2.14)	MU
Pos	732.544	7.57	1.48 (1.17 – 1.87)	**0.001**	1.53 (1.01 – 2.05)	M
Pos	732.599	7.83	1.64 (1.16 – 2.31)	**0.005**	1.71 (1.40 – 2.03)	MU
Pos	733.547	7.57	1.39 (1.11 – 1.72)	**0.004**	1.53 (1.05 – 2.01)	M

Pos	733.602	7.83	1.85 (1.29 – 2.67)	**0.001**	1.66 (1.16 – 2.16)	MU
Pos	744.544	7.50	1.54 (1.17 – 2.03)	**0.002**	2.01 (1.78 – 2.23)	M
Pos	745.547	7.50	1.50 (1.14 – 1.95)	**0.003**	1.93 (1.70 – 2.15)	M
Pos	746.559	7.73	1.65 (1.22 – 2.23)	**0.001**	1.59 (1.25 – 1.93)	M
Pos	747.563	7.73	1.61 (1.17 – 2.22)	**0.004**	1.65 (1.36 – 1.93)	M
Pos	752.511	7.35	1.64 (1.22 – 2.21)	**0.001**	1.52 (1.38 – 1.67)	M
Pos	756.544	7.45	1.43 (1.12 – 1.83)	**0.004**	1.86 (1.41 – 2.30)	M
Pos	757.547	7.45	1.41 (1.07 – 1.85)	**0.014**	1.82 (1.34 – 2.30)	M
Pos	757.611	7.88	1.91 (1.34 – 2.72)	**0.000**	1.73 (1.16 – 2.31)	MU
Pos	758.561	7.66	1.07 (0.92 – 1.24)	0.381	2.23 (1.91 – 2.54)	MU
Pos	759.563	7.66	1.82 (1.27 – 2.60)	**0.001**	2.12 (1.68 – 2.56)	MU
Pos	760.576	7.89	2.27 (1.45 – 3.56)	**0.000**	1.86 (1.46 – 2.27)	MU
Pos	761.579	7.89	1.79 (1.22 – 2.63)	**0.003**	1.76 (1.48 – 2.04)	MU
Pos	762.581	7.89	1.98 (1.28 – 3.05)	**0.002**	1.96 (1.54 – 2.38)	M
Pos	763.584	7.89	2.68 (1.64 – 4.37)	**0.000**	2.03 (1.65 – 2.40)	MU
Pos	768.543	7.46	1.32 (1.03 – 1.69)	**0.031**	1.66 (1.20 – 2.12)	M
Pos	770.558	7.57	1.87 (1.27 – 2.75)	**0.002**	1.90 (1.41 – 2.38)	M
Pos	770.955	6.41	1.18 (0.92 – 1.49)	0.190	1.99 (1.76 – 2.22)	MU
Pos	772.066	7.89	1.30 (1.07 – 1.58)	**0.009**	1.61 (0.91 – 2.31)	M
Pos	772.574	7.80	1.49 (1.12 – 1.99)	**0.006**	1.79 (1.43 – 2.15)	M
Pos	773.577	7.80	1.48 (1.09 – 2.01)	**0.013**	1.62 (1.34 – 1.90)	M
Pos	774.645	8.41	1.64 (1.28 – 2.11)	**0.000**	1.53 (1.09 – 1.96)	M
Pos	782.559	7.60	1.34 (1.07 – 1.68)	**0.012**	1.56 (1.20 – 1.91)	M
Pos	783.562	7.59	1.33 (0.98 – 1.79)	0.064	1.73 (1.56 – 1.91)	M
Pos	784.575	7.73	1.63 (1.07 – 2.49)	**0.023**	2.13 (1.83 – 2.43)	MU
Pos	785.578	7.73	1.65 (1.15 – 2.37)	**0.007**	2.19 (1.93 – 2.45)	MU
Pos	786.591	7.98	1.33 (1.00 – 1.76)	**0.048**	1.90 (1.51 – 2.29)	M
Pos	787.594	7.98	1.68 (1.19 – 2.39)	**0.004**	1.83 (1.53 – 2.14)	M
Pos	788.596	7.98	1.63 (1.09 – 2.45)	**0.018**	1.56 (1.21 – 1.90)	M
Pos	799.657	8.47	1.39 (1.14 – 1.70)	**0.001**	1.57 (0.82 – 2.32)	M
Pos	800.605	8.16	1.34 (1.04 – 1.73)	**0.025**	1.53 (1.11 – 1.95)	M
Pos	811.592	7.92	1.47 (1.15 – 1.88)	**0.002**	1.60 (1.20 – 2.00)	M
Pos	821.638	8.47	2.14 (1.51 – 3.02)	**0.000**	1.68 (1.15 – 2.20)	M
Pos	829.561	4.86	0.93 (0.78 – 1.09)	0.365	1.55 (0.47 – 2.63)	M
Pos	829.686	4.87	0.91 (0.75 – 1.10)	0.324	1.57 (0.51 – 2.62)	M
Pos	829.811	4.86	0.91 (0.76 – 1.09)	0.316	1.55 (0.50 – 2.59)	M
Pos	829.937	4.86	0.88 (0.73 – 1.05)	0.161	1.59 (0.46 – 2.71)	M
Pos	833.638	8.32	1.46 (1.05 – 2.02)	**0.026**	1.51 (0.92 – 2.10)	M
Pos	947.781	4.86	0.93 (0.77 – 1.11)	0.414	1.59 (0.74 – 2.44)	M
Pos	947.925	4.87	0.93 (0.77 – 1.12)	0.418	1.53 (0.47 – 2.60)	M
Pos	948.067	4.87	0.89 (0.73 – 1.07)	0.218	1.54 (0.38 – 2.70)	M
Pos	948.354	4.86	1.01 (0.86 – 1.19)	0.922	1.54 (0.46 – 2.62)	M
Pos	948.497	4.86	0.91 (0.75 – 1.10)	0.341	1.53 (0.55 – 2.51)	M
Pos	948.640	4.86	0.86 (0.71 – 1.05)	0.145	1.52 (0.56 – 2.48)	M
Pos	987.625	6.26	0.97 (0.86 – 1.11)	0.690	1.60 (0.29 – 2.92)	M
Pos	991.657	6.41	1.15 (0.99 – 1.33)	0.065	1.63 (0.60 – 2.66)	M
Pos	992.661	6.41	1.11 (0.93 – 1.32)	0.243	1.68 (0.67 – 2.69)	M
Pos	993.663	6.41	1.11 (0.93 – 1.33)	0.230	1.60 (0.62 – 2.59)	M
Pos	994.665	6.41	1.11 (0.92 – 1.34)	0.278	1.67 (0.67 – 2.67)	M
Pos	995.667	6.41	1.13 (0.93 – 1.37)	0.216	1.52 (0.63 – 2.42)	M
Pos	1005.67	6.47	1.05 (0.89 – 1.23)	0.566	1.65 (0.51 – 2.79)	M
Pos	1006.67	6.48	1.02 (0.87 – 1.20)	0.787	1.67 (0.59 – 2.74)	M
Pos	1015.65	6.39	1.10 (0.95 – 1.27)	0.189	1.61 (0.46 – 2.76)	M
Pos	1016.65	6.38	1.06 (0.91 – 1.23)	0.469	1.61 (0.45 – 2.77)	M
Pos	1017.67	6.44	1.07 (0.90 – 1.27)	0.420	1.59 (0.58 – 2.61)	M
Pos	1019.67	6.44	1.10 (0.91 – 1.32)	0.321	1.63 (0.64 – 2.63)	M
Pos	1045.60	6.30	1.02 (0.84 – 1.23)	0.883	1.51 (0.06 – 2.96)	M
Pos	1046.61	6.30	1.05 (0.87 – 1.27)	0.601	1.51 (0.07 – 2.94)	M
Pos	1063.65	6.31	1.03 (0.88 – 1.21)	0.731	1.55 (0.62 – 2.47)	M
Pos	1064.65	6.31	1.05 (0.90 – 1.22)	0.543	1.54 (0.64 – 2.45)	M
Pos	1066.33	7.51	1.12 (0.94 – 1.34)	0.217	1.69 (0.77 – 2.62)	M

Pos	1069.60	6.29	1.03 (0.86 – 1.22)	0.789	1.59 (0.92 – 2.27)	M
Pos	1087.65	6.30	1.09 (0.94 – 1.26)	0.266	1.54 (0.37 – 2.70)	M
Pos	1088.65	6.30	1.06 (0.91 – 1.23)	0.441	1.53 (0.32 – 2.74)	M
Pos	1089.66	6.32	0.97 (0.81 – 1.16)	0.720	1.50 (0.18 – 2.82)	M
Pos	1090.66	6.32	1.00 (0.83 – 1.20)	0.977	1.51 (0.59 – 2.43)	M
Pos	1105.57	4.87	0.93 (0.78 – 1.12)	0.456	1.54 (0.32 – 2.75)	M

M, multivariate statistics, orthogonal projections to latent structures-effect projections (OPLS-EP) model with cross-validated analysis of variance (CV-ANOVA), $p<0.05$, VIP ≥1.5, and VIP 95%CI >0; U, univariate statistics, two-sided Wilcoxon signed-rank test with Bonferroni correction, $p<0.05$. CLR, conditional logistic regression; OR, odds ratio by doubling; RT, retention time; VIP, variable importance on projection.

Supplementary Table 3. *Association between discriminating features identified by Wilcoxon signed-rank tests and prostate cancer risk*

Feature			Overall prostate cancer (752 pairs)			
**Polarity**	***m/z***	**RT (min)**	**p-value[a]**	**OR (95% CI)**	**CLR p-**	**Statistical**
Neg	368.2656	6.53	8.58×10^{-8}	0.78 (0.62 –	**0.044**	MU
Pos	189.0715	2.24	1.67×10^{-5}	1.63 (1.16 –	**0.005**	U
Pos	226.0424	1.16	4.17×10^{-6}	1.68 (1.26 –	**0.001**	U
Pos	227.0764	2.24	1.70×10^{-6}	1.50 (1.11 –	**0.008**	U
Pos	229.2139	6.50	5.11×10^{-6}	1.31 (1.09 –	**0.003**	MU
Pos	257.1486	6.11	8.71×10^{-12}	1.42 (1.11 –	**0.005**	MU
Pos	257.2450	6.69	2.25×10^{-8}	1.39 (1.12 –	**0.003**	MU
Pos	258.2480	6.69	2.10×10^{-6}	1.35 (1.09 –	**0.007**	MU
Pos	289.1510	5.92	4.26×10^{-5}	1.23 (1.02 –	**0.029**	U
Pos	326.2420	5.92	2.20×10^{-5}	1.24 (1.02 –	**0.032**	U
Pos	369.3478	7.70	4.19×10^{-6}	1.28 (1.02 –	**0.031**	MU
Pos	434.6860	0.74	2.73×10^{-5}	1.38 (1.05 –	**0.020**	U
Pos	697.5171	7.24	1.54×10^{-6}	1.27 (1.04 –	**0.018**	MU
Pos	698.5202	7.24	2.24×10^{-7}	1.53 (1.17 –	**0.002**	MU
Pos	703.5678	7.51	4.50×10^{-6}	1.11 (0.92 –	0.287	MU
Pos	704.5703	7.51	8.78×10^{-6}	1.14 (0.96 –	0.127	MU
Pos	705.5726	7.51	1.70×10^{-5}	1.37 (1.07 –	**0.014**	MU
Pos	716.5688	7.43	1.47×10^{-5}	1.34 (1.10 –	**0.003**	MU
Pos	727.5653	7.35	4.22×10^{-5}	1.39 (1.17 –	**0.000**	MU
Pos	729.5813	7.57	3.05×10^{-7}	1.35 (1.09 –	**0.007**	MU
Pos	730.5291	7.36	3.32×10^{-6}	1.32 (1.13 –	**0.001**	MU
Pos	730.5843	7.57	2.93×10^{-7}	1.62 (1.24 –	**0.000**	MU
Pos	731.5320	7.36	3.86×10^{-6}	1.24 (1.07 –	**0.004**	MU
Pos	732.5447	7.57	5.41×10^{-6}	1.35 (1.13 –	**0.001**	MU
Pos	733.5478	7.57	3.54×10^{-6}	1.33 (1.12 –	**0.001**	MU
Pos	744.5441	7.50	2.24×10^{-5}	1.39 (1.13 –	**0.001**	MU
Pos	745.5479	7.50	8.39×10^{-7}	1.39 (1.14 –	**0.001**	MU
Pos	752.5110	7.35	7.68×10^{-6}	1.44 (1.17 –	**0.001**	MU
Pos	756.5441	7.45	2.50×10^{-6}	1.33 (1.11 –	**0.002**	MU
Pos	757.5473	7.45	1.53×10^{-7}	1.31 (1.08 –	**0.006**	MU
Pos	757.6113	7.88	1.39×10^{-5}	1.29 (1.02 –	**0.033**	MU
Pos	758.5509	7.44	3.73×10^{-6}	1.14 (1.00 –	0.051	U
Pos	758.5611	7.66	2.66×10^{-7}	1.02 (0.91 –	0.790	MU
Pos	759.5638	7.66	2.21×10^{-8}	1.30 (1.10 –	**0.002**	MU
Pos	760.5662	7.65	2.93×10^{-5}	1.17 (0.96 –	0.115	U
Pos	760.5764	7.65	2.90×10^{-5}	1.24 (0.96 –	0.100	MU
Pos	761.5687	7.65	8.00×10^{-6}	1.21 (1.00 –	**0.046**	U
Pos	761.5791	7.89	1.05×10^{-8}	1.38 (1.07 –	**0.013**	MU
Pos	763.5842	7.89	6.37×10^{-6}	1.44 (1.07 –	**0.018**	MU
Pos	768.5431	7.46	2.42×10^{-6}	1.32 (1.09 –	**0.005**	MU
Pos	769.5480	7.46	1.03×10^{-5}	1.33 (1.11 –	**0.003**	U
Pos	770.5586	7.57	1.22×10^{-6}	1.41 (1.11 –	**0.005**	MU
Pos	785.5783	7.73	2.79×10^{-6}	1.29 (1.06 –	**0.009**	MU
Pos	786.5914	7.98	1.66×10^{-5}	1.17 (0.98 –	0.078	MU
Pos	788.5965	7.98	3.29×10^{-6}	1.35 (1.05 –	**0.019**	U
Pos	832.5727	7.59	3.21×10^{-5}	1.34 (1.10 –	**0.004**	U

Feature			Non-aggressive prostate cancer (587 pairs)			
**Polarity**	***m/z***	**RT (min)**	**p-value[a]**	**OR (95% CI)**	**CLR p-**	**Statistical**
Neg	368.2656	6.53	2.78×10^{-6}	0.84 (0.64 –	0.184	MU
Pos	226.9491	0.64	1.23×10^{-5}	2.17 (1.21 –	0.096	U
Pos	257.1486	6.11	1.06×10^{-8}	1.33 (1.01 –	**0.044**	MU
Pos	257.2450	6.69	4.02×10^{-7}	1.37 (1.07 –	**0.011**	MU
Pos	258.2480	6.69	9.66×10^{-6}	1.33 (1.04 –	**0.021**	MU
Pos	697.5171	7.24	4.13×10^{-5}	1.23 (0.98 –	0.079	MU
Pos	698.5202	7.24	7.18×10^{-6}	1.48 (1.09 –	**0.011**	MU
Pos	703.5678	7.51	1.68×10^{-6}	1.19 (0.98 –	0.088	MU
Pos	704.5703	7.51	5.94×10^{-6}	1.13 (0.95 –	0.167	MU

Pos	705.5726	7.51	2.35×10^{-6}	1.46 (1.08 –	**0.013**	MU
Pos	729.5813	7.57	1.45×10^{-6}	1.40 (1.08 –	**0.012**	MU
Pos	730.5843	7.57	7.54×10^{-6}	1.61 (1.19 –	**0.002**	MU
Pos	745.5479	7.50	4.59×10^{-6}	1.40 (1.12 –	**0.003**	MU
Pos	757.5473	7.45	3.29×10^{-5}	1.31 (1.06 –	**0.012**	MU
Pos	759.5638	7.66	1.60×10^{-5}	1.25 (1.05 –	**0.013**	MU
Pos	761.5791	7.89	2.98×10^{-5}	1.53 (1.12 –	**0.008**	MU
Pos	770.5586	7.57	2.76×10^{-5}	1.49 (1.13 –	**0.004**	MU
**Feature**			**Aggressive prostate cancer (165 pairs)**			
**Polarity**	***m/z***	**RT (min)**	**p-value[a]**	**OR (95% CI)**	**CLR p-**	**Statistical**
Pos	188.0685	2.24	1.53×10^{-5}	2.05 (0.88 –	0.097	U
Pos	189.0715	2.24	2.60×10^{-5}	2.44 (1.00 –	**0.050**	U
Pos	205.0950	2.24	1.09×10^{-5}	2.46 (1.03 –	**0.043**	U
Pos	206.0978	2.24	2.49×10^{-5}	2.44 (1.06 –	**0.035**	U
Pos	227.0764	2.24	4.10×10^{-5}	2.13 (0.90 –	0.085	U
Pos	409.1826	2.24	8.21×10^{-6}	1.37 (0.89 –	0.158	U
**Feature**			**Younger subgroup of 40- and 50-year-olds at baseline (326 pairs)**			
**Polarity**	***m/z***	**RT (min)**	**p-value[a]**	**OR (95% CI)**	**CLR p-**	**Statistical**
Neg	857.6637	8.67	6.87×10^{-6}	0.96 (0.83 –	0.569	MU
Neg	887.6739	8.69	4.14×10^{-5}	0.83 (0.67 –	0.089	MU
Pos	216.9203	0.68	4.34×10^{-5}	5.49 (1.56 –	**0.008**	MU
Pos	226.0434	1.16	7.20×10^{-6}	1.41 (0.94 –	0.093	MU
Pos	226.9491	0.64	5.11×10^{-9}	4.16 (15.0 –	**0.006**	MU
Pos	227.0764	2.24	1.69×10^{-5}	1.34 (0.89 –	0.162	MU
Pos	257.1486	6.11	5.06×10^{-6}	0.96 (0.67 –	0.836	MU
Pos	303.2265	6.61	1.83×10^{-5}	1.23 (1.00 –	**0.048**	MU
Pos	305.2423	6.73	1.82×10^{-5}	1.19 (0.95 –	0.138	MU
Pos	384.1153	0.72	3.55×10^{-5}	1.28 (0.81 –	0.286	MU
Pos	392.7913	0.68	2.10×10^{-5}	1.46 (0.79 –	0.223	MU
Pos	402.8202	0.68	1.46×10^{-5}	1.86 (1.00 –	**0.048**	MU
Pos	460.7781	0.68	3.66×10^{-5}	1.36 (0.89 –	0.151	MU
**Feature**			**Older subgroup of 60-year-olds at baseline (426 pairs)**			
**Polarity**	***m/z***	**RT (min)**	**p-value[a]**	**OR (95% CI)**	**CLR p-**	**Statistical**
Neg	290.2108	5.45	1.59×10^{-5}	1.38 (0.89 –	0.156	U
Neg	311.2002	6.16	2.89×10^{-5}	1.29 (1.10 –	**0.002**	MU
Neg	356.1849	6.23	4.08×10^{-8}	1.23 (1.10 –	**0.000**	MU
Neg	368.2656	6.53	2.66×10^{-7}	0.54 (0.37 –	**0.002**	MU
Neg	412.2472	5.95	3.67×10^{-6}	1.18 (1.04 –	**0.008**	MU
Pos	187.0554	0.80	4.51×10^{-5}	1.54 (1.19 –	**0.001**	U
Pos	257.1486	6.11	2.26×10^{-7}	1.56 (1.20 –	**0.000**	MU
Pos	257.2450	6.69	3.26×10^{-6}	2.30 (1.35 –	**0.000**	MU
Pos	522.7918	6.41	3.97×10^{-8}	1.33 (1.10 –	**0.000**	MU
Pos	638.5643	8.55	3.40×10^{-5}	1.92 (1.36 –	**0.006**	MU
Pos	643.5195	8.55	1.88×10^{-5}	1.75 (1.28 –	**0.001**	MU
Pos	698.5202	7.24	1.14×10^{-5}	1.66 (1.22 –	**0.000**	MU
Pos	729.5813	7.57	5.73×10^{-6}	1.92 (1.15 –	**0.005**	MU
Pos	730.5843	7.57	3.95×10^{-6}	2.57 (1.47 –	**0.000**	MU
Pos	731.5968	7.83	1.81×10^{-5}	1.98 (1.37 –	**0.007**	MU
Pos	732.5998	7.83	3.32×10^{-5}	1.66 (1.15 –	**0.005**	MU
Pos	733.6028	7.83	8.01×10^{-6}	1.24 (0.99 –	**0.001**	MU
Pos	757.6113	7.88	1.26×10^{-5}	1.75 (1.24 –	**0.000**	MU
Pos	758.5611	7.66	3.46×10^{-6}	1.37 (1.11 –	0.381	MU
Pos	759.5638	7.66	1.90×10^{-7}	1.63 (1.21 –	**0.001**	MU
Pos	759.6273	8.20	2.62×10^{-5}	1.35 (1.04 –	**0.024**	U
Pos	760.5764	7.89	4.34×10^{-6}	1.63 (1.03 –	**0.000**	MU
Pos	761.5687	7.65	6.26×10^{-6}	1.36 (1.10 –	**0.042**	U
Pos	761.5791	7.89	2.67×10^{-5}	1.24 (0.85 –	**0.003**	MU
Pos	763.5842	7.89	1.28×10^{-6}	1.54 (1.19 –	**0.000**	MU
Pos	770.9559	6.41	2.07×10^{-5}	1.56 (1.20 –	0.190	MU

Pos	784.5755	7.73	1.61×10^{-6}	2.30 (1.35 –	**0.023**	MU
Pos	785.5783	7.73	2.26×10^{-7}	1.33 (1.10 –	**0.007**	MU
Pos	786.5809	7.72	1.99×10^{-5}	1.92 (1.36 –	**0.041**	U

[a]Features significant by two-sided Wilcoxon signed rank test with Bonferroni correction for 1100 tests. CLR, conditional logistic regression; OR, odds ratio by doubling. RT, retention time. M, multivariate statistics, orthogonal projections to latent structures-effect projections (OPLS-EP) model with cross-validated analysis of variance (CV-ANOVA), p<0.05, variable importance on projection (VIP) ≥1.5 and VIP 95%CI >0; U, univariate statistics, two-sided Wilcoxon signed-rank test with Bonferroni correction, p<0.05.

Supplementary Table 4. *Differential associations by age group and disease aggressiveness*

Feature ID[a]	Polarity	*m/z*	RT (min)	Group	OR (95% CI)[b]	CLR p-value	N	$P_{heterogeneity}$[c]
**Stratification by disease aggressiveness (all ages at baseline)**								
m/z 257.1496	Pos	257.1496	6.11	Non-agg	1.33 (1.01 – 1.75)	0.044*	1174	0.311
				Agg	1.83 (1.05 – 3.19)	0.033*	330	
Palmitic acid	Pos	257.2477	6.69	Non-agg	1.37 (1.07 – 1.74)	0.011*	1174	0.731
				Agg	1.51 (0.92 – 2.47)	0.105	330	
SM 32:1	Pos	676.5456	7.24	Non-agg	1.26 (1.05 – 1.52)	0.015*	1174	0.775
				Agg	1.34 (0.95 – 1.88)	0.096	330	
SM 32:0	Pos	677.5557	7.36	Non-agg	1.28 (1.05 – 1.56)	0.013*	1173	0.603
				Agg	1.16 (0.84 – 1.61)	0.378	330	
SM 33:1	Pos	690.5607	7.37	Non-agg	1.33 (1.07 – 1.66)	0.012*	1174	0.825
				Agg	1.40 (0.94 – 2.09)	0.097	330	
SM 36:3	Pos	727.5725	7.35	Non-agg	1.37 (1.14 – 1.65)	0.001*	1174	0.730
				Agg	1.48 (0.99 – 2.22)	0.056	330	
SM 36:2	Pos	729.5886	7.57	Non-agg	1.40 (1.08 – 1.82)	0.012*	1172	0.586
				Agg	1.24 (0.87 – 1.76)	0.230	330	
PCaa 32:2	Pos	730.5363	7.36	Non-agg	1.34 (1.12 – 1.60)	0.002*	1174	0.719
				Agg	1.25 (0.90 – 1.73)	0.180	330	
PCaa 32:0	Pos	734.5659	7.84	Non-agg	1.49 (1.08 – 2.05)	0.015*	1174	0.296
				Agg	1.05 (0.60 – 1.85)	0.854	330	
PCaa 33:2	Pos	744.5528	7.50	Non-agg	1.38 (1.10 – 1.72)	0.005*	1173	0.910
				Agg	1.42 (0.90 – 2.24)	0.132	330	
PCaa 34:3	Pos	756.5520	7.45	Non-agg	1.31 (1.07 – 1.60)	0.008*	1174	0.762
				Agg	1.41 (0.93 – 2.13)	0.107	330	
PCaa 34:2	Pos	759.5702	7.65	Non-agg	1.25 (1.05 – 1.49)	0.013*	1174	0.319
				Agg	1.65 (0.98 – 2.76)	0.058	330	
PCaa 34:1	Pos	761.5867	7.89	Non-agg	1.53 (1.12 – 2.10)	0.008*	1173	0.141
				Agg	0.97 (0.57 – 1.64)	0.901	328	
PCaa 35:4	Pos	768.5583	7.45	Non-agg	1.34 (1.08 – 1.66)	0.008*	1173	0.767
				Agg	1.25 (0.80 – 1.94)	0.333	330	
PCaa 35:2	Pos	772.5731	7.80	Non-agg	1.28 (1.02 – 1.61)	0.036*	1174	0.995
				Agg	1.28 (0.78 – 2.11)	0.331	330	
PCaa 36:4 (A)	Pos	782.5683	7.56	Non-agg	1.21 (1.01 – 1.44)	0.039*	1174	0.802
				Agg	1.14 (0.77 – 1.70)	0.514	330	
PCaa 36:3 (A)	Pos	785.5855	7.72	Non-agg	1.24 (1.01 – 1.53)	0.040*	1171	0.536
				Agg	1.48 (0.89 – 2.48)	0.134	330	
PCaa 36:2 (B)	Pos	786.6000	7.93	Non-agg	1.25 (1.00 – 1.56)	0.047*	1168	0.202
				Agg	0.97 (0.70 – 1.34)	0.839	328	
**Stratification by baseline age (all disease aggressiveness types)**								
m/z 206.8938	Pos	206.8938	0.69	40- and 50-year-olds	3.13 (1.23 – 7.96)	0.017*	652	0.137
				60-year-olds	1.42 (0.90 – 2.24)	0.128	851	
m/z 216.9227	Pos	216.9227	0.65	40- and 50-year-olds	5.49 (1.56 – 19.3)	0.008*	652	0.056
				60-year-olds	1.50 (0.97 – 2.32)	0.069	851	
FA 18:2	Pos	303.2265	6.58	40- and 50-year-olds	1.23 (1.00 – 1.50)	0.048*	652	0.978
				60-year-olds	1.23 (1.02 – 1.50)	0.035*	852	
m/z 402.8240	Pos	402.8240	0.67	40- and 50-year-olds	1.86 (1.00 – 3.45)	0.048*	650	0.428
				60-year-olds	1.38 (0.92 – 2.07)	0.117	850	
m/z 412.8530	Pos	412.8530	0.65	40- and 50-year-olds	1.43 (1.07 – 1.92)	0.016*	651	0.790
				60-year-olds	1.34 (0.93 – 1.94)	0.117	850	
m/z 420.8840	Pos	420.8840	0.65	40- and 50-year-olds	1.39 (1.06 – 1.81)	0.017*	651	0.544
				60-year-olds	1.65 (1.00 – 2.74)	0.051	850	
SM 36:3†	Pos	727.5725	7.35	40- and 50-year-olds	1.43 (1.06 – 1.94)	0.020*	652	0.818
				60-year-olds	1.37 (1.12 – 1.68)	0.003*	852	
Glucose	Neg	179.0555	0.72	40- and 50-year-olds	1.43 (0.83 – 2.47)	0.202	650	0.012*
				60-year-olds	0.54 (0.32 – 0.91)	0.022*	848	
m/z 187.0575	Pos	187.0575	0.80	40- and 50-year-olds	0.99 (0.71 – 1.37)	0.941	652	0.036*
				60-year-olds	1.54 (1.19 – 2.00)	0.001*	852	
Uric acid	Pos	191.0178	0.96	40- and 50-year-olds	0.80 (0.62 – 1.04)	0.092	652	<0.001*
				60-year-olds	1.56 (1.20 – 2.03)	<0.001*	852	

Palmitic acid	Pos	257.2477	6.69	40- and 50-year-olds	1.06 (0.77 – 1.46)	0.730	652	0.027*
				60-year-olds	1.75 (1.28 – 2.38)	<0.001*	852	
m/z 301.1418	Pos	301.1418	5.96	40- and 50-year-olds	0.48 (0.27 – 0.84)	0.010*	652	<0.001*
				60-year-olds	1.92 (1.15 – 3.21)	0.013*	852	
m/z 380.2565	Pos	380.2565	6.23	40- and 50-year-olds	0.88 (0.68 – 1.13)	0.305	652	0.007*
				60-year-olds	1.37 (1.11 – 1.67)	0.003*	852	
m/z 643.5256	Pos	643.5256	8.55	40- and 50-year-olds	0.72 (0.55 – 0.94)	0.014*	652	<0.001*
				60-year-olds	1.58 (1.22 – 2.05)	0.001*	852	
SM 32:1	Pos	676.5456	7.24	40- and 50-year-olds	1.17 (0.85 – 1.62)	0.333	652	0.536
				60-year-olds	1.32 (1.09 – 1.61)	0.006*	852	
SM 32:0	Pos	677.5557	7.36	40- and 50-year-olds	1.12 (0.81 – 1.53)	0.498	652	0.420
				60-year-olds	1.30 (1.07 – 1.59)	0.009*	851	
SM 33:1	Pos	689.5571	7.37	40- and 50-year-olds	1.01 (0.72 – 1.41)	0.974	852	0.348
				60-year-olds	1.21 (1.01 – 1.44)	0.035*	850	
SM(d18:2/16:0)	Pos	701.5567	7.29	40- and 50-year-olds	1.03 (0.76 – 1.41)	0.844	652	0.548
				60-year-olds	1.14 (1.00 – 1.30)	0.040*	852	
SM(d18:1/16:0)	Pos	704.5703	7.51	40- and 50-year-olds	0.87 (0.62 – 1.21)	0.398	652	0.059
				60-year-olds	1.31 (1.00 – 1.70)	0.048*	846	
SM 36:3†	Pos	727.5725	7.35	40- and 50-year-olds	1.43 (1.06 – 1.94)	0.020*	652	0.818
				60-year-olds	1.37 (1.12 – 1.68)	0.003*	852	
SM 36:2	Pos	729.5886	7.57	40- and 50-year-olds	1.00 (0.67 – 1.50)	0.995	652	0.089
				60-year-olds	1.56 (1.14 – 2.12)	0.005*	850	
PCaa 32:2	Pos	730.5363	7.36	40- and 50-year-olds	1.21 (0.93 – 1.56)	0.155	652	0.429
				60-year-olds	1.38 (1.13 – 1.68)	0.002*	852	
SM 36:1	Pos	731.6042	7.83	40- and 50-year-olds	0.69 (0.44 – 1.08)	0.105	652	0.005*
				60-year-olds	1.49 (1.12 – 1.98)	0.007*	851	
PCaa 32:1	Pos	732.5515	7.57	40- and 50-year-olds	1.17 (0.89 – 1.55)	0.262	652	0.211
				60-year-olds	1.48 (1.17 – 1.87)	0.001*	852	
PCaa 33:2	Pos	744.5528	7.50	40- and 50-year-olds	1.19 (0.89 – 1.59)	0.232	652	0.208
				60-year-olds	1.54 (1.17 – 2.03)	0.002*	851	
PCaa 33:1	Pos	746.5693	7.72	40- and 50-year-olds	0.91 (0.65 – 1.27)	0.573	652	0.010*
				60-year-olds	1.65 (1.22 – 2.23)	0.001*	852	
PCaa 34:3	Pos	756.5520	7.45	40- and 50-year-olds	1.18 (0.90 – 1.55)	0.223	652	0.309
				60-year-olds	1.43 (1.12 – 1.83)	0.004*	852	
PCaa 34:2	Pos	759.5702	7.65	40- and 50-year-olds	1.00 (0.80 – 1.25)	0.986	652	0.006*
				60-year-olds	1.82 (1.27 – 2.60)	0.001*	852	
SM 38:1	Pos	759.6327	8.20	40- and 50-year-olds	0.84 (0.59 – 1.20)	0.336	652	0.042*
				60-year-olds	1.30 (1.04 – 1.64)	0.024*	849	
PCaa 34:1	Pos	760.5812	7.89	40- and 50-year-olds	0.75 (0.50 – 1.12)	0.157	651	<0.001*
				60-year-olds	2.27 (1.45 – 3.56)	<0.001*	843	
PCaa 35:4	Pos	768.5562	7.45	40- and 50-year-olds	1.33 (0.98 – 1.81)	0.067	652	0.963
				60-year-olds	1.32 (1.03 – 1.69)	0.031*	851	
PCaa 35:2	Pos	772.5831	7.80	40- and 50-year-olds	1.02 (0.74 – 1.39)	0.919	652	0.076
				60-year-olds	1.49 (1.12 – 2.00)	0.007*	852	
PCaa 36:4 (B)	Pos	782.5655	7.60	40- and 50-year-olds	0.97 (0.73 – 1.28)	0.817	652	0.079
				60-year-olds	1.34 (1.07 – 1.68)	0.012*	852	

PCaa 36:3 (B)	Pos	784.5808	7.73	40- and 50-year-olds	0.93 (0.75 – 1.16)	0.532	652	0.021*
				60-year-olds	1.63 (1.07 – 2.49)	0.023*	842	
PCaa 36:2 (A)	Pos	787.6013	7.98	40- and 50-year-olds	1.03 (0.77 – 1.37)	0.831	652	0.035*
				60-year-olds	1.68 (1.19 – 2.39)	0.004*	852	
SM 41:2	Pos	799.6635	8.47	40- and 50-year-olds	0.94 (0.70 – 1.27)	0.698	652	0.034*
				60-year-olds	1.39 (1.14 – 1.70)	0.001*	851	
PCaa 38:4	Pos	810.5960	7.92	40- and 50-year-olds	0.93 (0.72 – 1.19)	0.548	651	0.011*
				60-year-olds	1.47 (1.15 – 1.88)	0.002*	851	

[a]Listed features found to be significant in either subgroup after stratification. The detected features were subjected to multistep filtering and analysis by orthogonal projection of latent structures-effect projections (OPLS-EP). For each subgroup three models were constructed, *i.e.*, a model containing all cases, a model excluding the extreme 1% of cases based on Hotelling's T^2 range, and a model excluding the extreme 5% of cases. Model significance was determined by cross-validated analysis of variance (CV-ANOVA), with $p<0.05$ considered significant. Features in at least one of the three models with variable importance on projection (VIP) ≥1.5 and VIP 95%CI >0 were considered to be significant for discrimination between cases and controls. A univariate approach with two-sided Wilcoxon signed-rank test with Bonferroni correction for 1100 tests was also applied to find discriminating features. Features in common between the statistical methods, with conditional logistic regression (CLR) p-value >0.05 or affected by covariate inclusion in the model, were filtered. Isotope and adduct filtering was done by assessing whether features with the same retention time differed in *m/z* corresponding to ^{13}C isotope differences ($\Delta=n\times1.0033$), Na^+-adducts ($\Delta=21.9819$), or in-source H_2O ($\Delta=18.0153$) or NH_3 ($\Delta=17.0266$)-loss, retaining (by decreasing priority) the $[M+H]^+$ adduct, the lowest *m/z* isotopologue, or an in-source fragment rather than Na^+-adduct. [b]Odds ratio (OR) per signal doubling. [c]Differential associations by age group or disease type were compared by Wald test, i.e. comparing regression coefficients between the respective subgroups (Baseline age: 40 and 50 years, or 60 years, Disease type: Non-aggressive or aggressive), with p-values <0.05 considered significant (indicated with an asterisk). †Features in common between age stratifications. RT, retention time. N, number of samples in which the feature was detected.

Supplementary Table 5. *MS^2 data and annotation of features associated with prostate cancer risk*

Feature ID[a]	Fatty acid chain ID	Polarity	*m/z*	RT (min)	*m/z* error (ppm)	MS^2 pos fragments	MS^2 neg fragments	MS^2 comments
**Features associated with prostate cancer risk (all 752 pairs)**								
Tryptophan	-	Pos	189.0731	2.24	3.70	118.0642, 146.0588, 170.0580	No Signal	Is M-NH_3 of 205.0943. Trp HMDB MS^2 match. Is Trp-NH_3+H. Probable uptake of H^+ by NH_2-group, which is then neutrally lost, and second hydrogen adduct formation on the indole nitrogen.
m/z 201.1847	-	Pos	201.1847	6.25	-	90.9475, 106.9431, 142.1576, 169.0806, 181.1127	No Signal	Unknown. Lauric acid at RT 5.7 in neg mode with HMDB MS^2 fit. Potential in-source fragment of lauric acid-containing species.
Tyrosine	-	Pos	226.0445	1.16	2.65	95.0845, 109.0636, 123.0787, 142.0844, 207.0525	No Signal	M+Na of 204.0616. 123 & 95 fragments in common with tyrosine. Co-elutes with tyrosine with METLIN MS^2 fit. M+2Na-H of tyrosine.
Myristic acid	-	Pos	229.2157	6.50	2.33	84.9584, 95.09846, 107.0847	227.2004	Neg MS^2 gives 227, which is the whole fatty acid. Loss of 12 Th in Pos mode. Potential in-source fragment.
m/z 257.1497	-	Pos	257.1497	6.11	-	95.0854, 107.0483, 109.0994, 123.0776, 147.1142, 159.0824, 191.1413, 244.1545, 259.1676, 261.1313	No signal	Unknown
Palmitic acid	-	Pos	257.2477	6.69	0.78	89.06, 95.0841, 103.0751	237.2224, 255.2342	Fits with METLIN MS^2. Potential in-source fragment.
m/z 289.1517	-	Pos	289.1517	5.95	-	90.9751, 106.0834, 211.0996, 226.2135, 229.1080, 247.1203, 265.1297, 277.1331	No signal	Unknown
Cholesterol	-	Pos	369.3522	7.69	1.62	189.1614, 203.1757, 215.1785, 229.1915, 243.2075, 257.2261, 287.2713	No signal	Fit with METLIN cholesterol MS^2. M-H_2O+H adduct
SM 32:1	-	Pos	676.5456	7.24	1.86	PC frag*, 226.9509, 228.9221	No signal	No LysoSM. Is M+1 of 675.5435. Accurate mass (Odd M+H) gives SM.

SM 32:0	-	Pos	677.5557	7.36	5.11	PC frag*, 288.9186, 226.9498	No signal	Accurate mass (Odd M+H) gives SM. Partial overlap with M+2 of 675.5419, SM 32:1, increases error.
SM 33:1	-	Pos	689.5571	7.37	3.05	PC frag*, 226.9498, 288.9193, 418.8929	No signal	No LysoSM. Accurate mass (Odd M+H) gives SM.
SM(d18:2/16:0)	d18:2 & 16:0	Pos	701.5567	7.29	3.57	PC frag*, 262.2531, 288.9199, 515.4629, 639.4528	No signal	Accurate mass (Odd M+H) gives SM. 262 indicates d18:2 fragment. Thus the other lipid is 16:0.
SM(d18:1/16:0)	d18:1 & 16:0	Pos	705.5784	7.51	3.97	PC frag*, 264.2639, 685.5641	No signal	M+2 of 703.5748, 264 is d18:1 fragment. Thus the other lipid is 16:0. Accurate mass (Odd M+H) gives
SM 34:1	-	Pos	715.5681	7.51	1.40	PC frag*, 502.5058, 520.5127, 542.4964, 666.4914, 703.5804, 722.5735, 725.5717	No signal	PC fragments. Is M+1 of 714.5693. Doubly charged species in cluster. 703.58 is also base peak in MS^1. Could be 2M+H+Na adduct of 703.58, SM(d18:1/16:0).
SM 36:3	-	Pos	727.5725	7.35	3.18	PC frag*, 226.9495, 288.9204, 468.3108, 541.4801, 665.4709	No signal	PC fragments, M+3 formation fits with other SM spectra in HMDB. 541 & 665 are 2 Th lighter than observed for SM 36:2.
SM 36:2	-	Pos	729.5886	7.57	3.40	PC frag*, 543.4940, 667.4875, 732.5543	No signal	PC fragments. M+3 formation. Accurate mass (Odd M+H) gives SM.
PCaa 32:2	14:0 & 18:2	Pos	730.5363	7.36	3.29	PC frag*, 226.9482, 288.9173, low 450.2983, low 468.2983, low 502.3371	No signal	PC fragments. 468 is LPC 14:0 and 450 is LPC 14:0-H_2O. 502 is LPC 18:2-H_2O (stronger). Probably PCaa(14:0/18:2).
SM 36:1	-	Pos	731.6042	7.83	3.47	PC frag*, 734.5675	No signal	PC fragments. No LPC fragments. M+3 visible and odd M+H.
PCaa 32:1	16:0 & 16:1	Pos	732.5515	7.57	3.05	PC frag*, 476.3266	No signal	PC fragments. 476 is LPC16:1-H_2O, thus other lipid should be 16:0. Probably PCaa(16:0/16:1).
PCaa 33:2	17:2 & 16:0	Pos	744.5528	7.50	1.31	PC frag*, 478.3366, 288.9195, 162.9540, 146.9802	No signal	PC fragments. 478 is LPC16:0-H_2O, thus the other lipid should be 17:2. Probably PCaa(17:2/16:0).
PCaa 33:1	-	Pos	746.5693	7.72	0.97	PC frag*, 364.2601, 385.2726	No signal	PC fragments. No good LPC signals. Accurate mass gives PCaa 33:1.

PCaa 34:3	18:3 & 16:0	Pos	756.5520	7.45	3.02	PC frag*, 478.3255 (low), 496.3404 (low)	No signal	PC fragments. 496 is LPC16:0, 478 is LPC16:0-H_2O, thus the other lipid should be 18:3. Probably PCaa(18:3/16:0).
SM 38:2	-	Pos	757.6194	7.89	3.18	PC frag*, 288.9184, 496.3349, 760.5895	No signal	M+3 fragment visible. Accurate mass (Odd M+H) gives SM.
PCaa 34:2	18:2 & 16:0	Pos	759.5702	7.65	3.48	PC frag*, 496.3363 (low)	No signal	PC fragments. 496 is LPC16:0, thus the other lipid should be 18:2. M+1 of 758.5694. Probably PCaa(18:2/16:0).
PCaa 34:1	18:1 & 16:0	Pos	761.5867	7.89	2.23	PC frag*, 478.53287, 496.2422, 504.3426	255.2332, 281.2524	M+1 of 760.585. 496 is LPC16:0. 478 is LPC16:0-H_2O. 504 is LPC18:1-H_2O. C16 signals stronger. Negative 255 and 281 are the fatty acids C16:0 and C18:1. Probably PCaa(18:1/16:0).
PCaa 35:4	-	Pos	768.5563	7.45	2.51	PC frag*, 226.9478, 288.9170	No signal	PC fragments. No LPC signals visible. Accurate mass indicates PCaa.
PCaa 35:2	19:2 & 16:0	Pos	772.5831	7.80	3.23	PC frag*, 288.9236. 478.3272	No Signal	PC fragments. 478 is LPC16:0-H_2O, thus the other lipid should be 19:2. Probably PCaa(19:2/16:0).
PCaa 36:3 (A)	18:1 & 18:2	Pos	785.5855	7.73	3.76	PC frag*, 319.1954, 337.2018, 478.2600, 502.3289, 522.3516, 598.4924	279.2304, 281.2488	PC fragments. M+1 of 784.5851. Neg loss of fatty acids C18:2 and C18:1. 279 stronger. 522 is LPC18:1. 502 is LPC18:2-H_2O. Probably PCaa(18:1/18:2).
PCaa 36:2 (A)	18:0 & 18:2	Pos	787.6013	7.98	3.56	PC frag*, 502.3285, 524.3755	279.2266	502 is LPC18:2-H_2O, 279 is loss C18:2. Other chain should thus be C18:0. Probably PCaa(18:0/18:2).
PCaa 40:7	24:7 & 16:0	Pos	832.5809	7.59	5.02	PC frag*, 478.3294, 647.499, 745.6886, 771.6725, 804.5533	253.2140, 255.2315, 277.2158, 279.2307, 291.2296, 417.2384, 669.4467, 695.4648, 739.5161, 754.5392	478 is LPC16:0-H_2O. Neg loss of C16:0, C16:1, C18:2, C18:3, and C19:3 visible. Structure not clear. Presence of 478 indicates PCaa(24:7/16:0).

Feature ID[a]	Fatty acid chain ID	Polarity	*m/z*	RT (min)	*m/z* error (ppm)	MS^2 pos fragments	MS^2 neg fragments	MS^2 comments
**Features associated with prostate cancer risk upon stratification by disease aggressiveness (587 non-aggressive pairs only)**								
m/z 257.1496	-	Pos	257.1496	6.11	-	95.0854, 107.0483, 109.0994, 123.0776, 147.1142, 159.0824, 191.1413, 244.1545, 259.1676, 261.1313	No signal	Unknown
Palmitic acid	-	Pos	257.2477	6.69	0.78	89.0600, 95.0841, 103.0751	237.2224, 255.2342	Fits with METLIN MS^2. Potential in-source fragment.
SM 32:1	-	Pos	676.5456	7.24	1.86	PC frag*, 226.9509, 228.9221	No signal	No LysoSM. Is M+1 of 675.5435. Accurate mass (Odd M+H) gives SM.
SM 32:0	-	Pos	677.5557	7.36	5.11	PC frag*, 226.9498, 288.9186	No signal	Accurate mass (Odd M+H) gives SM. Partial overlap with M+2 of 675.5419, SM 32:1, increases error.
SM 33:1	-	Pos	690.5607	7.37	2.63	PC frag*, 226.9509, 228.9221	No signal	No LysoSM. Accurate mass (Odd M+H) gives SM.
SM 36:3	-	Pos	727.5725	7.35	3.18	PC frag*, 226.9495, 288.9204, 468.3108, 541.4801, 665.4709	No signal	PC fragments, M+3 formation fits with other SM spectra in HMDB. 541 & 665 are 2 Th lighter than observed for SM 36:2.
SM 36:2	-	Pos	729.5886	7.57	3.40	PC frag*, 543.4940, 667.4875, 732.5543	No signal	PC fragments. M+3 formation. Accurate mass (Odd M+H) gives SM.
PCaa 32:2	14:0 & 18:2	Pos	730.5363	7.36	3.29	PC frag*, 226.9482, 288.9173, low 468.2983, low 450.2983, low 502.3371	No signal	PC fragments. 468 is LPC 14:0 and 450 is LPC 14:0-H_2O. 502 is LPC 18:2-H_2O (stronger). Probably PCaa(14:0/18:2).
PCaa 32:0	-	Pos	734.5681	7.84	2.99	No MS^2 collected	No MS^2 collected	MS^2 not collected due to stronger co-eluting compound within the 0.5 *m/z* window of the quadrupole. Two isobaric compounds visible. This elutes later and is stronger. Probable PCaa. Accurate mass and retention time points towards PCaa 32:0.
PCaa 33:2	17:2 & 16:0	Pos	744.5528	7.50	1.31	PC frag*, 146.9802, 162.9540, 288.9195, 478.3366,	No signal	PC fragments. 478 is LPC16:0-H_2O, thus the other lipid should be 17:2. Probably PCaa(17:2/16:0).
PCaa 34:3	18:3 & 16:0	Pos	756.5520	7.45	3.02	PC frag*, 478.3255 (low), 496.3404 (low)	No signal	PC fragments. 496 is LPC16:0, 478 is LPC16:0-H_2O, thus the other lipid should be 18:3. Probably PCaa(18:3/16:0).

PCaa 34:2	18:2 & 16:0	Pos	759.5702	7.65	3.48	PC frag*, 496.3363 (low)	No signal	PC fragments. 496 is LPC16:0, thus the other lipid should be 18:2. M+1 of 758.5694. Probably PCaa(18:2/16:0).
PCaa 34:1	18:1 & 16:0	Pos	761.5867	7.89	2.23	PC frag*, 478.3287, 496.2422, 504.3426	255.2332, 281.2524	M+1 of 760.585. 496 is LPC16:0. 478 is LPC16:0-H_2O. 504 is LPC18:1-H_2O. C16 signals stronger. Negative 255 and 281 are the fatty acids C16:0 and C18:1. Probably PCaa(18:1/16:0).
PCaa 35:4	-	Pos	768.5583	7.45	2.51	PC frag*, 226.9478, 288.9170	No signal	PC fragments. No LPC signals visible. Accurate mass gives PCaa.
PCaa 35:2	19:2 & 16:0	Pos	772.5831	7.80	3.23	PC frag*, 288.9236. 478.3272	No Signal	PC fragments. 478 is LPC16:0-H_2O, thus the other lipid should be 19:2. Probably PCaa(19:2/16:0).
PCaa 36:4 (A)	20:4 & 16:0	Pos	782.5683	7.56	2.43	PC frag*, 478.3272, 496.3522, 526.3271, 544.3472 (weak)	255.2313	PC fragments. 496 is LPC16:0. 478 is LPC16:0-H_2O. 544&526 are LPC20:4 and LPC20:4-H_2O. 496 and 478 stronger. Probably PCaa(20:4/16:0).
PCaa 36:3 (A)	18:1 & 18:2	Pos	785.5855	7.72	3.76	PC frag*, 319.1954, 337.2018, 478.260, 502.3289, 522.3516, 598.4924	279.2304, 281.2488	PC fragments. M+1 of 784.5851. Neg loss of fatty acids C18:2 and C18:1. 279 stronger. 522 is LPC18:1. 502 is LPC18:2-H_2O. Probably PCaa(18:1/18:2).
PCaa 36:2 (B)	20:2 & 16:0	Pos	786.6000	7.93	0.89	PC frag*, 478.3402, 502.3285, 504.3468, 524.3755	No Signal	PC fragments. 478 is LPC16:0-H_2O. Thus the other lipid should be 20:2. Probably PCaa(20:2/16:0).
**Feature ID[a]**	**Fatty acid chain ID**	**Polarity**	***m/z***	**RT (min)**	***m/z* error (ppm)**	**MS^2 pos fragments**	**MS^2 neg fragments**	**MS^2 comments**
**Features associated with prostate cancer risk upon stratification by baseline age (younger subgroup of 326 pairs with 40- and 50-year-olds at baseline)**								
m/z 206.8938	-	Pos	206.8938	0.68	-	80.9485, 138.9067, 146.9798, 150.0138	No signal	Unknown
m/z 216.9227	-	Pos	216.9227	0.65	-	80.9485, 82.9451, 90.9763, 148.9329, 159.0477, 177.0587	No signal	Unknown
Fatty acid 18:2	-	Pos	303.2297	6.58	0.82	198.9675, 210.9678, 216.9778, 228.9790, 244.0245, 258.0422	79.9572	M+Na of Fatty acid 18:2. Characteristic pattern with loss of 14 and 12 Th. Potential in-source fragment.
m/z 402.8240	-	Pos	402.8240	0.67	-	90.9765, 138.9064, 158.9638, 196.8659, 206.8937, 216.9223	No signal	Unknown
m/z 412.8530	-	Pos	412.8530	0.65	-	90.9768, 158.9634, 216.9224, 280.0942	No signal	Unknown

Feature ID[a]	Fatty acid chain ID	Polarity	*m/z*	RT (min)	*m/z* error (ppm)	MS² pos fragments	MS² neg fragments	MS² comments
m/z 420.8840	-	Pos	420.8840	0.65	-	90.9769, 158.9641, 216.9221, 226.9518	No signal	Unknown
SM 36:3	-	Pos	727.5725	7.35	3.18	PC frag*, 226.9495, 288.9204, 468.3108, 541.4801, 665.4709	No signal	PC fragments, M+3 formation fits with other SM spectra in HMDB. 541 & 665 are 2 Th lighter than observed for SM 36:2.
**Feature ID[a]**	**Fatty acid chain ID**	**Polarity**	***m/z***	**RT (min)**	***m/z* error (ppm)**	**MS² pos fragments**	**MS² neg fragments**	**MS² comments**
**Features associated with prostate cancer risk upon stratification by baseline age (older subgroup of 426 pairs with 60-year-olds at baseline)**								
Glucose	-	Neg	179.0555	0.72	3.35	No signal	89.0246, 122.0327	89 ion validates hexose. Glucose validation from other hexoses not possible by MS², but glucose is the most abundant hexose in plasma.
m/z 187.0575	-	Pos	187.0575	0.80	-	84.0800, 109.0635, 114.0904, 123.0776, 130.0842, 140.1395, 144.1104	122.9659	Unknown
Uric acid	-	Pos	191.0178	0.96	1.25	M+H fragments: 98.0343, 126.0277, 141.0385, 152.0067	No signal	M+Na of Uric acid. HMDB MS² fit.
Palmitic acid	-	Pos	257.2477	6.69	0.78	89.06, 95.0841, 103.0751	237.2224, 255.2342	Fits with METLIN MS². Potential in-source fragment.
m/z 301.1418	-	Pos	301.1418	5.95	-	95.0840, 104.1065, 184.0718, 214.1108, 240.0980, 245.0812, 259.1201, 265.1329, 282.2797, 299.1802	No signal	184 and 104 suggest phosphorylcholine substructure. Unknown.
m/z 380.2565	-	Pos	380.2565	6.23	-	No MS² collected	No signal	MS² not collected due to low signal intensity. Accurate mass and retention time point towards sphingosine-1-phosphate.
m/z 643.5256	-	Pos	643.5256	8.55	-	90.9759, 104.1060, 158.9635, 220.9290, 226.9478, 262.2478, 288.9193, 300.9151	No signal	Accurate mass suggests diacylglycerol 38:5. No reference hit on MS² of diacylglycerols found. Unknown.
SM 32:1	-	Pos	676.5456	7.24	1.86	PC frag*, 226.9509, 228.9221	No signal	No LysoSM. Is M+1 of 675.5435. Accurate mass (Odd M+H) gives SM.
SM 32:0	-	Pos	677.5557	7.36	5.11	PC frag*. 226.9498, 288.9186	No signal	Accurate mass (Odd M+H) gives SM. Partial overlap with M+2 of 675.5419, SM 32:1, increases error.

SM 33:1	-	Pos	689.5571	7.37	3.05	PC frag*, 226.9498, 288.9193, 418.8929	No signal	No LysoSM. Accurate mass (Odd M+H) gives SM.
SM(d18:2/16:0)	d18:2 & 16:0	Pos	701.5567	7.29	3.57	PC frag*, 262.2531, 288.9199, 515.4629, 639.4528	No signal	Accurate mass (Odd M+H) gives SM. 262 indicates d18:2 fragment. Thus the other lipid is 16:0.
SM(d18:1/16:0)	d18:1 & 16:0	Pos	704.5764	7.51	2.51	PC frag*, 264.2639, 685.5641	No signal	M+2 of 703.5748, 264 is d18:1 fragment. Thus the other lipid is 16:0. Accurate mass (Odd M+H) gives SM.
SM 36:3	-	Pos	727.5725	7.35	3.18	PC frag*, 226.9495, 288.9204, 468.3108, 541.4801, 665.4709	No signal	PC fragments, M+3 formation fits with other SM spectra in HMDB. 541 & 665 are 2 Th lighter than observed for SM 36:2.
SM 36:2	-	Pos	729.5886	7.57	3.40	PC frag*, 543.4940, 667.4875, 732.5543	No signal	PC fragments. M+3 formation. Accurate mass (Odd M+H) gives SM.
PCaa 32:2	14:0 & 18:2	Pos	730.5363	7.36	3.29	PC frag*, 226.9482, 288.9173, low 450.2983, low 468.2983, low 502.3371	No signal	PC fragments. 468 is LPC 14:0 and 450 is LPC 14:0-H_2O. 502 is LPC 18:2-H_2O (stronger). Probably PCaa(14:0/18:2).
SM 36:1	-	Pos	731.6042	7.83	3.47	PC frag*, 734.5675	No signal	PC fragments. No LPC fragments. M+3 visible and odd M+H.
PCaa 32:1	16:0 & 16:1	Pos	732.5515	7.57	3.05	PC frag*, 476.3266	No signal	PC fragments. 476 is LPC16:1-H_2O, thus other lipid should be 16:0. Probably PCaa(16:0/16:1).
PCaa 33:2	17:2 & 16:0	Pos	744.5528	7.50	1.31	PC frag*, 288.9195, 478.3366	No signal	PC fragments. 478 is LPC16:0-H_2O, thus the other lipid should be 17:2. Probably PCaa(17:2/16:0).
PCaa 33:1	-	Pos	746.5693	7.72	0.97	PC frag*, 364.2601, 385.2726	No signal	PC fragments. No good LPC signals. Accurate mass gives PCaa 33:1.
PCaa 34:3	18:3 & 16:0	Pos	756.5520	7.45	3.02	PC frag*, 478.3255 (low), 496.3404 (low)	No signal	PC fragments. 496 is LPC16:0, 478 is LPC16:0-H_2O, thus the other lipid should be 18:3. Probably PCaa(18:3/16:0).
PCaa 34:2	18:2 & 16:0	Pos	759.5702	7.65	3.48	PC frag*, 496.3363 (low)	No signal	PC fragments. 496 is LPC16:0, thus the other lipid should be 18:2. M+1 of 758.5694. Probably PCaa(18:2/16:0).
SM 38:1	-	Pos	759.6338	8.20	1.45	PC frag*, 226.9506, 288.9227, 350.8963, 356.9118, 418.8801, 424.8975, 486.8691	No signal	PC fragments. M+3 formation. Accurate mass (Odd M+H) gives SM.

PCaa 34:1	18:1 & 16:0	Pos	760.5825	7.89	1.71	PC frag*, 478.3282, 496.3326. 504.3414, 522.3487	255.2221, 281.2488	M+1 of 760.585. 496 is LPC16:0. 478 is LPC16:0-H2O. 504 is LPC18:1-H2O. C16 signals stronger. Negative 255 and 281 are the fatty acids C16:0 and C18:1. Probably PCaa(18:1/16:0).
PCaa 35:4	-	Pos	768.5563	7.45	2.51	PC frag*, 226.9478, 288.9170	No signal	PC fragments. No LPC signals visible. Accurate mass gives PCaa.
PCaa 35:2	19:2 & 16:0	Pos	772.5831	7.80	3.23	PC frag*, 288.9236. 478.3272	No Signal	PC fragments. 478 is LPC16:0-H_2O, thus the other lipid should be 19:2. Probably PCaa(19:2/16:0).
PCaa 36:4 (B)	18:0 & 18:4	Pos	782.5668	7.60	1.66	PC frag*	285.2186, 317.2491, 616.4721, 642.4834	285 is negative loss of C18:0. Other chain thus C18:4. Position unknown since no detected LPC:s.
PCaa 36:3 (B)	20:3 & 16:0	Pos	784.5817	7.73	1.15	PC frag*, 319.1954, 337.2018, 349.1954, 398.7624, 478.326, 496.3352, 502.3289, 522.3516, 598.4843, 729.4812	255.2330, 305.2399	255 and 281 are neg loss of fatty acids C16:0 and C20:3. 255 stronger. 496 is LPC16:0, 478 is LPC16:0-H_2O. Probably PCaa(20:3/16:0).
PCaa 36:2 (A)	18:0 & 18:2	Pos	787.6013	7.98	3.56	PC frag*, 502.3285, 524.3755	279.2266	502 is LPC18:2-H_2O, 279 is loss C18:2. Other chain should thus be C18:0. Probably PCaa(18:0/18:2).
SM 41:2	-	Pos	799.6645	8.47	1.25	PC frag*, 226.9517, 288.9235, 416.8690, 418.8814, 424.8935, 486.8578	No Signal	Accurate mass (Odd M+H) gives SM.
PCaa 38:4	22:4 & 16:0	Pos	810.5971	7.92	1.36	PC frag*, 478.3316, 506.3631, 526.329, 627.5326, 649.5159, 773.5072	255.2320	255 is neg loss of fatty acid C16:0. 478 is LPC16:0-H_2O. Other chain should thus be 22:4. Probably PCaa(22:4/16:0)

[a]Listed features were found to be significant in either subgroup after stratification. *Phosphorylcholine (PC, *m/z* 184.0733) forms the following indicatory fragments: 86.097, 104.106, and 125.001. Comparisons of spectral data with databases (Human Metabolome Database (HMDB), METLIN, mzCloud, Lipid Maps, MassBank) were conducted in order to assist annotation. For annotation of phospholipid features not included in databases, annotation was assisted by previously presented guidelines (Godzien et al., 2015). When two different filtered features with the same sum of side-chain carbons and unsaturations were annotated, they were given an additional suffix ((A) or (B)) in order to distinguish them. The annotation of individual side-chains (when applicable) was determined based on the accurate masses of detected lysophosphatidylcholine and lysosphingomyelin CID fragments. The relative positions of the phospholipids side-chains were suggested based on the accurate mass of the lysophosphatidylcholine fragments, with the stronger signal corresponding to the more stable secondary carbocation formed by loss of the side-chain from the C_2 of the glycerol backbone (*sn*-2 loss). MS^2, tandem mass spectrometry with collisional induced dissociation fragmentation; LPC, lysophosphatidylcholine; PCaa, diacyl-phosphatidylcholine; RT, retention time; SM, sphingomyelin.

Supplementary Table 6. *Compilation of data for filtered features associated with prostate cancer risk*

Feature ID	FA chains[a]	Suggested position ID[a]	Polarity	*m/z*	RT (min)	*m/z* error (ppm)	OR (95% CI)	CLR p-value	VIP (95% CI)	Univar p-value	Statistical procedure	MSI ID level
**Features associated with prostate cancer risk (all 752 pairs)**												
Tryptophan	-	-	Pos	189.0731	2.24	3.70	1.63 (1.16 – 2.28)	0.005	1.27 (0.78 – 1.75)	1.67×10^{-5}	U	2
m/z 201.1847	-	-	Pos	201.1847	6.25	-	1.28 (1.07 – 1.54)	0.007	1.57 (0.93 – 2.22)	1.43×10^{-4}	M	
Tyrosine	-	-	Pos	226.0445	1.16	2.65	1.68 (1.26 – 2.25)	0.001	1.19 (0.88 – 1.50)	4.17×10^{-6}	U	2
Myristic acid	-	-	Pos	229.2157	6.50	2.33	1.31 (1.09 – 1.57)	0.003	1.63 (0.93 – 2.32)	5.11×10^{-6}	MU	2
m/z 257.1497	-	-	Pos	257.1497	6.11	-	1.42 (1.11 – 1.81)	0.005	2.26 (1.53 – 2.99)	8.71×10^{-12}	MU	4
Palmitic acid	-	-	Pos	257.2477	6.69	0.78	1.39 (1.12 – 1.73)	0.003	1.76 (1.28 – 2.24)	2.25×10^{-8}	MU	2
m/z 289.1517	-	-	Pos	289.1517	5.95	-	1.23 (1.02 – 1.48)	0.029	1.05 (0.42 – 1.69)	4.26×10^{-5}	U	4
Cholesterol	-	-	Pos	369.3522	7.69	1.62	1.28 (1.02 – 1.60)	0.031	1.56 (0.83 – 2.28)	4.19×10^{-6}	MU	2
SM 32:1	-	-	Pos	676.5456	7.24	1.86	1.28 (1.09 – 1.51)	0.003	1.76 (1.10 – 2.42)	2.90×10^{-4}	M	3
SM 32:0	-	-	Pos	677.5557	7.36	5.11	1.25 (1.06 – 1.48)	0.009	1.71 (1.09 – 2.33)	2.45×10^{-3}	M	3
SM 33:1	-	-	Pos	689.5571	7.37	3.05	1.17 (1.00 – 1.35)	0.047	1.86 (1.22 – 2.49)	1.02×10^{-4}	M	3
SM(d18:2/16:0)	d18:2 & 16:0	SM(d18:2/16:0)	Pos	701.5567	7.29	3.57	1.13 (1.00 – 1.26)	0.042	1.74 (1.05 – 2.43)	1.68×10^{-3}	M	2
SM (d18:1/16:0)	d18:1 & 16:0	SM(d18:1/16:0)	Pos	705.5784	7.51	3.97	1.37 (1.07 – 1.76)	0.014	2.04 (1.50 – 2.58)	1.70×10^{-5}	MU	2
SM 34:1	-	-	Pos	715.5681	7.51	1.40	1.35 (1.11 – 1.64)	0.003	1.85 (1.31 – 2.38)	8.79×10^{-5}	M	3
SM 36:3	-	-	Pos	727.5725	7.35	3.18	1.39 (1.17 – 1.65)	0.000	1.58 (1.03 – 2.12)	4.22×10^{-5}	MU	3
SM 36:2	-	-	Pos	729.5886	7.57	3.40	1.35 (1.09 – 1.68)	0.007	2.09 (1.71 – 2.48)	3.05×10^{-7}	MU	3
PCaa 32:2	14:0 & 18:2	PCaa(14:0/18:2)	Pos	730.5363	7.36	3.29	1.32 (1.13 – 1.54)	0.001	1.75 (1.27 – 2.23)	3.32×10^{-6}	MU	3
SM 36:1	-	-	Pos	731.6042	7.83	3.47	1.23 (1.01 – 1.50)	0.041	1.68 (1.15 – 2.21)	1.45×10^{-4}	M	3
PCaa 32:1	16:1 & 16:0	PCaa(16:0/16:1)	Pos	732.5515	7.57	3.05	1.35 (1.13 – 1.61)	0.001	1.54 (0.86 – 2.22)	5.41×10^{-6}	MU	3
PCaa 33:2	17:2 & 16:0	PCaa(17:2/16:0)	Pos	744.5528	7.50	1.31	1.39 (1.13 – 1.70)	0.001	2.14 (1.87 – 2.40)	2.24×10^{-5}	MU	3
PCaa 33:1	-	-	Pos	746.5693	7.72	0.97	1.28 (1.04 – 1.59)	0.022	1.55 (1.18 – 1.92)	1.77×10^{-3}	M	3
PCaa 34:3	18:3 & 16:0	PCaa(18:3/16:0)	Pos	756.5520	7.45	3.02	1.33 (1.11 – 1.59)	0.002	1.96 (1.57 – 2.34)	2.50×10^{-6}	MU	3
SM 38:2	-	-	Pos	757.6194	7.89	3.18	1.29 (1.02 – 1.62)	0.033	1.77 (1.53 – 2.02)	1.39×10^{-5}	MU	3

PCaa 34:2	18:2 & 16:0	PCaa(18:2/16:0)	Pos	759.5702	7.65	3.48	1.30 (1.10 – 1.54)	0.002	2.19 (1.70 – 2.68)	2.21×10^{-8}	MU	3
PCaa 34:1	18:1 & 16:0	PCaa(18:1/16:0)	Pos	761.5867	7.89	2.23	1.38 (1.07 – 1.77)	0.013	1.83 (1.29 – 2.37)	1.05×10^{-8}	MU	3
PCaa 35:4	-	-	Pos	768.5563	7.45	2.51	1.32 (1.09 – 1.61)	0.005	1.70 (1.25 – 2.16)	2.42×10^{-6}	MU	3
PCaa 35:2	19:2 & 16:0	PCaa(19:2/16:0)	Pos	772.5831	7.80	3.23	1.28 (1.04 – 1.58)	0.021	1.85 (1.56 – 2.13)	2.72×10^{-3}	M	3
PCaa 36:3 (A)	18:1 & 18:2	PCaa(18:1/18:2)	Pos	785.5855	7.73	3.76	1.29 (1.06 – 1.56)	0.009	2.22 (1.73 – 2.71)	2.79×10^{-6}	MU	3
PCaa 36:2 (A)	18:0 & 18:2	PCaa(18:0/18:2)	Pos	787.6013	7.98	3.56	1.35 (1.10 – 1.67)	0.005	1.92 (1.49 – 2.35)	1.16×10^{-3}	M	3
PCaa 40:7	16:0 & 24:7	PCaa(16:0/24:7)	Pos	832.5809	7.59	5.02	1.34 (1.10 – 1.62)	0.003	1.55 (0.95 – 2.15)	3.21×10^{-5}	U	3

**Feature ID**	**FA chains[a]**	**Suggested position ID[a]**	**Polarity**	***m/z***	**RT (min)**	***m/z* error (ppm)**	**OR (95% CI)**	**CLR p-value**	**VIP (95% CI)**	**Univar p-value**	**Statistical procedure**	**MSI ID level**
**Features associated with prostate cancer risk upon stratification by disease aggressiveness (587 non-aggressive pairs only)**												
m/z 257.1496	-	-	Pos	257.1496	6.11	-	1.33 (1.01 – 1.74)	0.044	2.26 (1.58 – 2.95)	1.06×10^{-8}	MU	4
Palmitic acid	-	-	Pos	257.2477	6.69	0.78	1.37 (1.07 – 1.74)	0.011	1.68 (1.56 – 1.80)	4.02×10^{-7}	MU	2
SM 32:1	-	-	Pos	676.5456	7.24	1.86	1.26 (1.05 – 1.52)	0.015	1.63 (1.04 – 2.21)	4.51×10^{-3}	M	3
SM 32:0	-	-	Pos	677.5557	7.36	5.11	1.28 (1.05 – 1.56)	0.013	1.59 (0.93 – 2.25)	1.33×10^{-2}	M	3
SM 33:1	-	-	Pos	690.5607	7.37	2.63	1.33 (1.07 – 1.66)	0.012	1.80 (1.05 – 2.55)	7.88×10^{-4}	M	3
SM 36:3	-	-	Pos	727.5725	7.35	3.18	1.37 (1.14 – 1.65)	0.001	1.56 (0.72 – 2.41)	5.07×10^{-4}	M	3
SM 36:2	-	-	Pos	729.5886	7.57	3.40	1.40 (1.08 – 1.82)	0.012	2.17 (1.62 – 2.72)	1.45×10^{-6}	MU	3
PCaa 32:2	14:0 & 18:2	PCaa(14:0/18:2)	Pos	730.5363	7.36	3.29	1.34 (1.12 – 1.60)	0.002	1.60 (1.15 – 2.05)	1.17×10^{-3}	M	3
PCaa 32:0	-	-	Pos	734.5681	7.84	2.99	1.49 (1.08 – 2.05)	0.015	1.53 (1.11 – 1.95)	1.67×10^{-2}	M	3
PCaa 33:2	17:2 & 16:0	PCaa(17:2/16:0)	Pos	744.5528	7.50	1.31	1.38 (1.10 – 1.72)	0.005	2.11 (1.57 – 2.66)	1.39×10^{-4}	M	3
PCaa 34:3	18:3 & 16:0	PCaa(18:3/16:0)	Pos	756.5520	7.45	3.02	1.31 (1.07 – 1.60)	0.008	1.89 (1.61 – 2.17)	2.79×10^{-4}	M	3
PCaa 34:2	18:2 & 16:0	PCaa(18:2/16:0)	Pos	759.5702	7.65	3.48	1.25 (1.05 – 1.49)	0.013	2.20 (1.83 – 2.57)	1.60×10^{-5}	MU	3
PCaa 34:1	18:1 & 16:0	PCaa(18:1/16:0)	Pos	761.5867	7.89	2.23	1.53 (1.12 – 2.10)	0.008	1.92 (1.30 – 2.53)	2.98×10^{-5}	MU	3

Feature ID	FA chains[a]	Suggested position ID[a]	Polarity	*m/z*	RT (min)	*m/z* error (ppm)	OR (95% CI)	CLR p-value	VIP (95% CI)	Univar p-value	Statistical procedure	MSI ID level
PCaa 35:4	-	-	Pos	768.5583	7.45	2.51	1.34 (1.08 – 1.66)	0.008	1.69 (1.33 – 2.04)	5.49×10^{-5}	M	3
PCaa 35:2	19:2 & 16:0	PCaa(19:2/16:0)	Pos	772.5831	7.80	3.23	1.28 (1.02 – 1.61)	0.036	1.91 (1.11 – 2.71)	3.87×10^{-3}	M	3
PCaa 36:4 (A)	20:4 & 16:0	PCaa(20:4/16:0)	Pos	782.5683	7.56	2.43	1.21 (1.01 – 1.44)	0.039	1.50 (0.82 – 2.18)	3.79×10^{-2}	M	3
PCaa 36:3 (A)	18:1 & 18:2	PCaa(18:1/18:2)	Pos	785.5855	7.72	3.76	1.24 (1.01 – 1.53)	0.040	2.21 (1.94 – 2.49)	6.50×10^{-4}	M	3
PCaa 36:2 (B)	20:2 & 16:0	PCaa(20:2/16:0)	Pos	786.6000	7.93	0.89	1.25 (1.00 – 1.56)	0.047	1.92 (1.38 – 2.47)	3.40×10^{-4}	M	3
**Feature ID**	**FA chains[a]**	**Suggested position ID[a]**	**Polarity**	***m/z***	**RT (min)**	***m/z* error (ppm)**	**OR (95% CI)**	**CLR p-value**	**VIP (95% CI)**	**Univar p-value**	**Statistical procedure**	**MSI ID level**
**Features associated with prostate cancer risk upon stratification by baseline age (younger subgroup of 326 pairs with 40- and 50-year-olds at baseline)**												
m/z 206.8938	-	-	Pos	206.8938	0.68	-	3.13 (1.23 – 7.96)	0.017	2.18 (1.37 – 3.00)	2.15×10^{-4}	M	4
m/z 216.9227	-	-	Pos	216.9227	0.65	-	5.49 (1.56 – 19.3)	0.008	2.14 (1.09 – 3.18)	4.34×10^{-5}	MU	4
Fatty acid 18:2	-	-	Pos	303.2297	6.58	0.82	1.23 (1.00 – 1.51)	0.048	2.06 (1.26 – 2.86)	1.83×10^{-5}	MU	3
m/z 402.8240	-	-	Pos	402.8240	0.67	-	1.86 (1.00 – 3.45)	0.048	2.09 (1.13 – 3.04)	1.46×10^{-5}	MU	4
m/z 412.8530	-	-	Pos	412.8530	0.65	-	1.43 (1.07 – 1.92)	0.016	1.80 (1.11 – 2.49)	6.67×10^{-5}	M	4
m/z 420.8840	-	-	Pos	420.8840	0.65	-	1.39 (1.06 – 1.81)	0.017	1.84 (1.47 – 2.20)	2.26×10^{-3}	M	4
SM 36:3	-	-	Pos	727.5725	7.35	3.18	1.43 (1.06 – 1.94)	0.020	1.75 (0.76 – 2.74)	8.04×10^{-4}	M	3
**Feature ID**	**FA chains[a]**	**Suggested position ID[a]**	**Polarity**	***m/z***	**RT (min)**	***m/z* error (ppm)**	**OR (95% CI)**	**CLR p-value**	**VIP (95% CI)**	**Univar p-value**	**Statistical procedure**	**MSI ID level**
**Features associated with prostate cancer risk upon stratification by baseline age (older subgroup of 426 pairs with 60-year-olds at baseline)**												
Glucose	-	-	Neg	179.0555	0.72	3.35	0.54 (0.32 – 0.91)	0.022	1.56 (0.69 – 2.43)	2.57×10^{-3}	M	2
m/z 187.0575	-	-	Pos	187.0575	0.80	-	1.54 (1.19 – 2.00)	0.001	1.20 (0.43 – 1.84)	4.51×10^{-5}	U	4
Uric acid	-	-	Pos	191.0178	0.96	1.25	1.56 (1.20 – 2.03)	0.001	1.54 (0.57 – 2.52)	9.05×10^{-4}	M	2
Palmitic acid	-	-	Pos	257.2477	6.69	0.78	1.75 (1.28 – 2.38)	0.000	2.29 (1.92 – 2.66)	3.26×10^{-6}	MU	2
m/z 301.1418	-	-	Pos	301.1418	5.95	-	1.92 (1.15 – 3.21)	0.013	1.74 (1.40 – 2.07)	1.34×10^{-4}	M	4
m/z 380.2565	-	-	Pos	380.2565	6.23	-	1.37 (1.11 – 1.67)	0.003	1.51 (1.01 – 2.01)	8.72×10^{-3}	M	4
m/z 643.5256	-	-	Pos	643.5256	8.55	-	1.58 (1.22 – 2.05)	0.001	1.75 (1.42 – 2.08)	1.88×10^{-5}	MU	4
SM 32:1	-	-	Pos	676.5456	7.24	1.86	1.32 (1.09 – 1.61)	0.006	1.66 (1.14 – 2.18)	1.75×10^{-3}	M	3
SM 32:0	-	-	Pos	677.5557	7.36	5.11	1.30 (1.07 – 1.59)	0.009	1.58 (1.03 – 2.13)	1.44×10^{-2}	M	3
SM 33:1	-	-	Pos	689.5571	7.37	3.05	1.21 (1.01 – 1.44)	0.035	1.80 (1.40 – 2.20)	8.28×10^{-4}	M	3

SM(d18:2/16:0)	d18:2 & 16:0	SM(d18:2/16:0)	Pos	701.5567	7.29	3.57	1.14 (1.01 – 1.30)	0.040	1.66 (1.29 – 2.03)	1.19×10^{-2}	M	2
SM(d18:1/16:0)	d18:1 & 16:0	SM(d18:1/16:0)	Pos	704.5764	7.51	2.51	1.31 (1.00 – 1.70)	0.048	2.14 (1.96 – 2.33)	6.36×10^{-5}	M	2
SM 36:3	-	-	Pos	727.5725	7.35	3.18	1.37 (1.12 – 1.68)	0.003	1.51 (1.26 – 1.76)	4.04×10^{-3}	M	3
SM 36:2	-	-	Pos	729.5886	7.57	3.40	1.56 (1.14 – 2.12)	0.005	2.04 (1.78 – 2.31)	5.73×10^{-6}	MU	3
PCaa 32:2	14:0 & 18:2	PCaa(14:0/18:2)	Pos	730.5363	7.36	3.29	1.38 (1.13 – 1.68)	0.002	2.01 (1.69 – 2.33)	4.49×10^{-4}	M	3
SM 36:1	-	-	Pos	731.6042	7.83	3.47	1.49 (1.12 – 1.98)	0.007	1.70 (1.25 – 2.14)	1.81×10^{-5}	MU	3
PCaa 32:1	16:0 & 16:1	PCaa(16:0/16:1)	Pos	732.5515	7.57	3.05	1.48 (1.17 – 1.87)	0.001	1.53 (1.01 – 2.05)	4.64×10^{-5}	M	3
PCaa 33:2	17:2 & 16:0	PCaa(17:2/16:0)	Pos	744.5528	7.50	1.31	1.54 (1.17 – 2.03)	0.002	2.01 (1.78 – 2.23)	1.06×10^{-3}	M	3
PCaa 33:1	-	-	Pos	746.5693	7.72	0.97	1.65 (1.22 – 2.23)	0.001	1.59 (1.25 – 1.93)	5.72×10^{-4}	M	3
PCaa 34:3	18:3 & 16:0	PCaa(18:3/16:0)	Pos	756.5520	7.45	3.02	1.43 (1.12 – 1.83)	0.004	1.86 (1.41 – 2.30)	4.66×10^{-4}	M	3
PCaa 34:2	18:2 & 16:0	PCaa(18:2/16:0)	Pos	759.5702	7.65	3.48	1.82 (1.27 – 2.60)	0.001	2.12 (1.68 – 2.56)	1.90×10^{-7}	MU	3
SM 38:1	-	-	Pos	759.6338	8.20	1.45	1.30 (1.04 – 1.64)	0.024	1.44 (0.99 – 1.89)	2.62×10^{-5}	U	3
PCaa 34:1	18:1 & 16:0	PCaa(18:1/16:0)	Pos	760.5825	7.89	1.71	2.27 (1.45 – 3.56)	0.000	1.86 (1.46 – 2.27)	4.34×10^{-6}	MU	3
PCaa 35:4	-	-	Pos	768.5563	7.45	2.51	1.32 (1.03 – 1.69)	0.031	1.66 (1.20 – 2.12)	7.07×10^{-4}	M	3
PCaa 35:2	19:2 & 16:0	PCaa(19:2/16:0)	Pos	772.5831	7.80	3.23	1.49 (1.12 – 1.99)	0.006	1.79 (1.43 – 2.15)	8.81×10^{-3}	M	3
PCaa 36:4 (B)	18:0 & 18:4	-	Pos	782.5668	7.60	1.66	1.34 (1.07 – 1.68)	0.012	1.56 (1.20 – 1.91)	1.19×10^{-2}	M	3
PCaa 36:3 (B)	20:3 & 16:0	PCaa(20:3/16:0)	Pos	784.5817	7.73	1.15	1.63 (1.07 – 2.49)	0.023	2.13 (1.83 – 2.43)	1.61×10^{-6}	MU	3
PCaa 36:2 (B)	18:0 & 18:2	PCaa(18:0/18:2)	Pos	787.6013	7.98	3.56	1.68 (1.19 – 2.39)	0.004	1.83 (1.53 – 2.14)	1.82×10^{-3}	M	3
SM 41:2	-	-	Pos	799.6645	8.47	1.25	1.39 (1.14 – 1.70)	0.001	1.57 (0.82 – 2.32)	2.42×10^{-3}	M	3
PCaa 38:4	22:4 & 16:0	PCaa(22:4/16:0)	Pos	810.5971	7.92	1.36	1.47 (1.15 – 1.88)	0.002	1.60 (1.20 – 2.00)	9.93×10^{-4}	M	3

[a]See Supplementary Table 5 for details of side-chain identification and position suggestion. M, multivariate statistics, orthogonal projections to latent structures-effect projections (OPLS-EP) model with cross-validated analysis of variance (CV-ANOVA), p<0.05, VIP ≥1.5, and VIP 95%CI >0; U, univariate statistics, two-sided Wilcoxon signed-rank test with Bonferroni correction, p<0.05. CLR, conditional logistic regression; FA, fatty acid; MSI, Metabolomics Standards Initiative; OR, odds ratio by doubling; PCaa, diacyl-phosphatidylcholine; RT, retention time; SM, sphingomyelin; VIP, variable importance on projection.

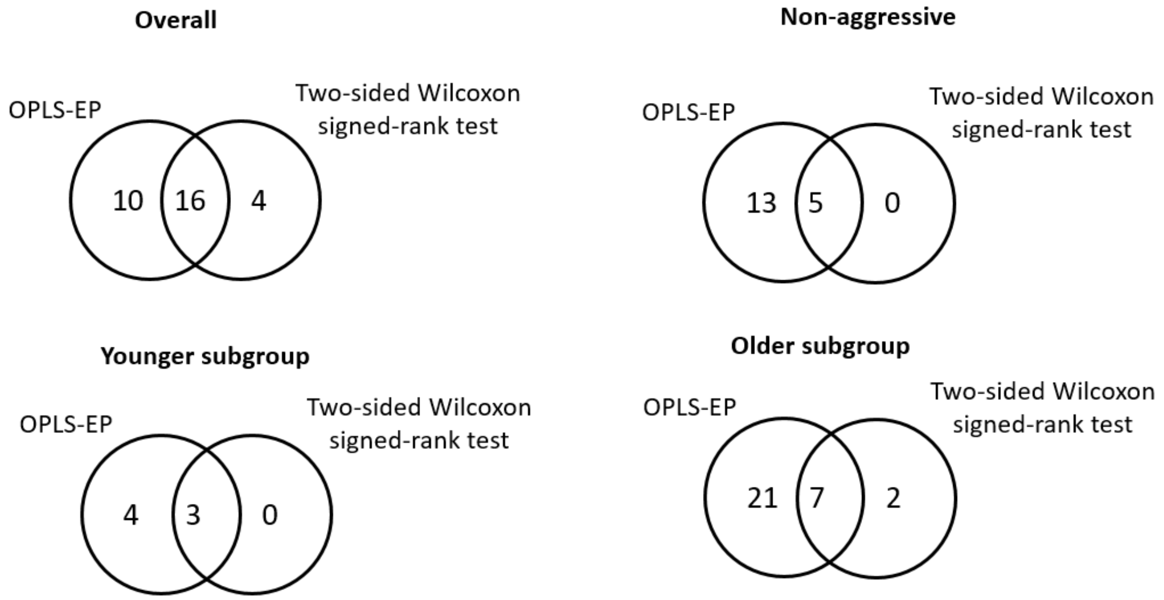

Supplementary Figure 1. The discriminating metabolite features found by multivariate statistical analyses (orthogonal projection to latent structures model-effect projections, OPLS-EP), univariate statistical analyses (two-sided Wilcoxon signed-rank test), or both. The features were then merged and subjected to a four-step filtering process (see **Figure 1**) in order to identify features significantly prospectively associated with prostate cacner risk. No discriminating metabolite feature was found for aggressive subgoup.
